# Global incidence and mortality of breast cancer: a trend analysis

**DOI:** 10.18632/aging.202502

**Published:** 2021-02-11

**Authors:** Junjie Huang, Paul SF Chan, Veeleah Lok, Xiao Chen, Hanyue Ding, Yinzi Jin, Jinqiu Yuan, Xiang-qian Lao, Zhi-Jie Zheng, Martin CS Wong

**Affiliations:** 1 The Jockey Club School of Public Health and Primary Care, Faculty of Medicine, Chinese University of Hong Kong, Hong Kong SAR, China; 2 Department of Global Health, School of Public Health, Peking University, Beijing, China; 3 Scientific Research Centre, The Seventh Affiliated Hospital, Sun Yat-sen University, Shenzhen, Guangdong, China; 4 School of Public Health, Zhengzhou University, Zhengzhou, Henan, China; 5 School of Public Health, The Chinese Academy of Medical Sciences and Peking Union Medical College, Beijing, China

**Keywords:** breast cancer, epidemiology, incidence, mortality, trend analysis

## Abstract

This study aimed to evaluate the global incidence and mortality trends of breast cancer among females by region and age in the past decade. We retrieved country-specific incidence and mortality data from the Global Cancer Observatory up to 2018 and Cancer Incidence in Five Continents volumes I-XI, the Nordic Cancer Registries, the Surveillance, Epidemiology, and End Results, and WHO mortality database up to 2016. The temporal patterns were using Average Annual Percent Change (AAPC) with the 95% confidence interval (CI) by joinpoint regression analysis. Most countries showed an increasing trend in incidence. For the older population aged ≥ 50 years, Japan (5.63, 4.90-6.36), Slovakia (3.63, 3.03-4.22), China (2.86, 2.00-3.72) reported the most prominent increase. For young females (<50 years), Japan (AAPC=3.81, 95% CI=2.71-4.93), Germany (AAPC=2.60, 95% CI=1.41-3.81) and Slovakia (1.91, 1.13-2.69) reported the most drastic rise. Similarly, 12 countries showed an incidence increase among women aged <40 years. As for mortality, the Philippines (4.36, 3.65-5.07), Thailand (4.35, 3.12-5.59), Colombia (0.75, 0.08-1.42), and Brazil (0.44, 0.19-0.68) reported a significant increase. The disease burden of breast cancer showed an increasing trend in a large number of populations. More preventive efforts are recommended for these countries. Further research should explore the underlying reasons for these epidemiological trends.

## INTRODUCTION

Worldwide, breast cancer is one of the leading causes of cancer morbidity and mortality. According to the status report on the GLOBOCAN 2018 estimates of cancer incidence and mortality, breast cancer was the second most commonly diagnosed malignancy, accounting for more than 11.6% of all female cancers [1]. It ranked as the fifth commonest cause of cancer deaths, leading to 6.6% of all cancer mortality worldwide. It induces a substantial public health burden, leading to a loss of 14.8 million Disability Adjusted Life Years (DALYs) [2–4]. The incidence of breast cancer is significantly higher in developed countries; globally, its age-standardized incidence rate was 54.5 per 100,000 female population in countries with high or very high Human Development Index (HDI) as compared to 31.3 in nations with low to medium HDI [1]. Its mortality rate remained the highest in the female population, representing the most commonly reported cause of female cancer deaths [1].

Studying the epidemiological trend of breast cancer is particularly valuable as it is highly preventable through a combination of primary or secondary preventive strategies [5, 6]. Major modifiable risk factors of breast cancer included obesity [7], physical inactivity [8], consumption of high protein diet such as red meat with exogenous hormones or carcinogenic byproducts [9], alcohol drinking [10], smoking [11], and use of oral contraceptive pills [12]. Most of these risk factors could be intervened through health education in clinical practice and public health initiatives.

Nevertheless, there is a scarcity of studies that have explored the most updated incidence and mortality of breast cancer on a global scale, as most recent evaluations are limited for certain populations [13, 14]. Whilst the Global Burden of Disease (GBD) studies [15–17] reported the global trends of breast cancer, 1) the data was based on modelling rather than using high quality cancer registries to evaluate the trend; 2) the trend results were not available for individual countries; 3) there was a lack of trend analysis by age groups. Recent studies have reported among younger populations a more drastic increase in incidence of colorectal cancer [18], which shares common risk factors with breast cancer. In some countries, preliminary findings indicated a more substantial incidence increase of breast cancer among younger individuals [13, 14, 19–21], but it is unknown whether this observation applies to the global population.

The objectives of this study were to evaluate the recent global incidence, mortality and temporal trend of breast cancer. We also examined whether younger populations show an increasing trend of incidence in different countries.

## RESULTS

### Incidence and mortality rates of breast cancer

**Figure 1:**
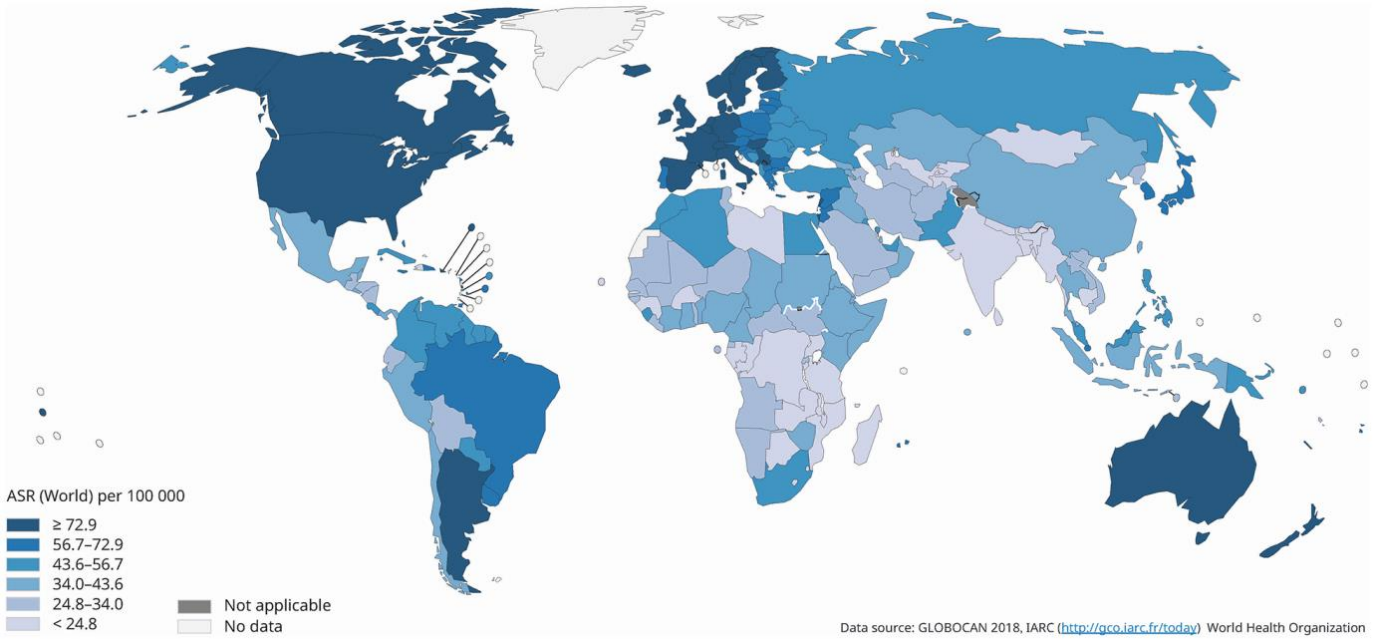
**Global estimated incidence of breast cancer in 2018, females, all ages.**

A total of 2,088,849 new cases of breast cancer and 626,679 related deaths were reported in 2018 (Supplementary Table 1). The global age-standardized rate of its incidence was 46.3 per 100,000 population and showed an almost four-fold variation worldwide (Figure 1). The highest rates were found in Australia and New Zealand (ASR (age-standardized rate)=94.2), Western Europe (ASR=92.6), Northern Europe (ASR=90.1) and North America (ASR=84.8), and the lowest were reported in South-Central Asia (ASR=25.9), Middle, Eastern and Western Africa (ASR=27.9 to 37.3), South-Eastern Asia (ASR=38.1) and Central America (ASR=38.3). The highest incidence tended to predominate in Western countries including Australia, Europe and America. The incidence of breast cancer was higher in countries with very high (ASR=75.2) or high HDI (ASR=40.1) as compared to those with low (ASR=32.8) or medium (ASR=30.7) HDI.

**Figure 2:**
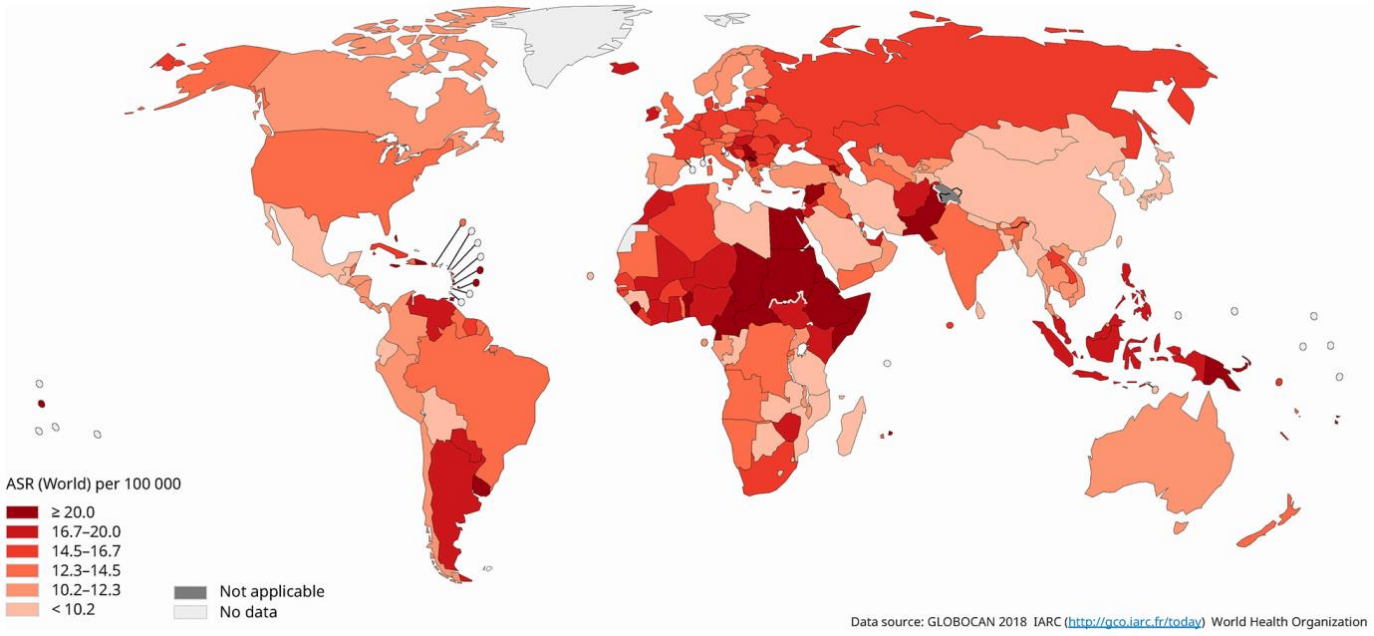
**Global estimated mortality of breast cancer in 2018, females, all ages.**

The global age-standardized rate of its mortality was 16.3 per 100,000 population and varied by three-fold in 2018 (Figure 2). The highest mortality rates were reported in Melanesia (ASR=25.5), Polynesia (ASR=21.6), Northern Africa (ASR=18.4), Caribbean (ASR=18.1), and Western Africa (ASR=17.8). The lowest estimated death rates were found in Eastern Asia (ASR=8.6), Central America (ASR=10.1), Australia and New Zealand (ASR=12.6), North America (ASR=12.6), and Southern America (ASR=13.4). The mortality of breast cancer was higher in countries with low (ASR=17.1) or medium HDI (ASR=14.3) as compared to those with very high (ASR=13.1) or high HDI (ASR=10.3).

### Temporal trends of breast cancer

**Figure 3:**
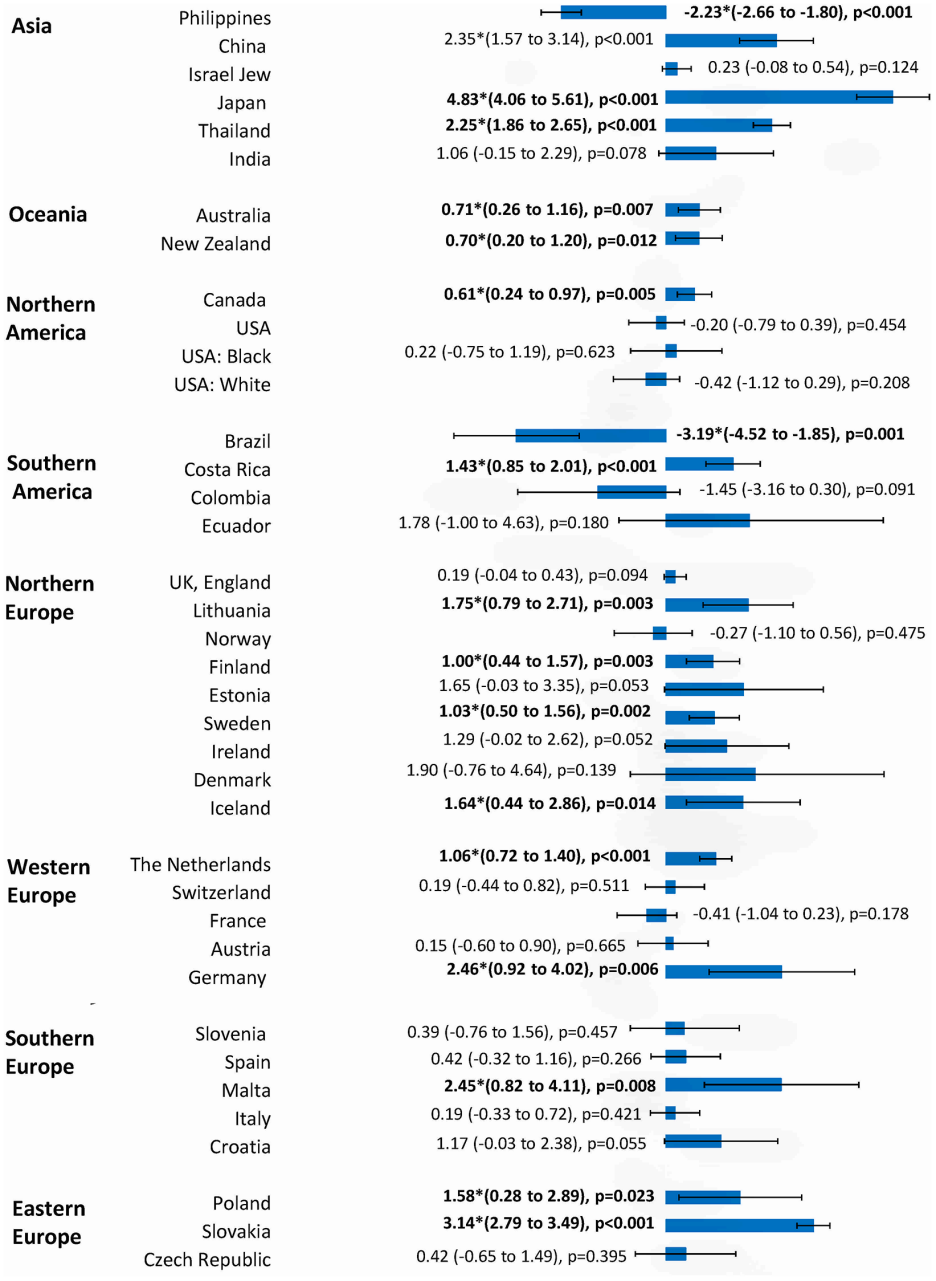
**The AAPC of the incidence of breast cancer in individuals aged 0-85+ years.**

**Figure 4:**
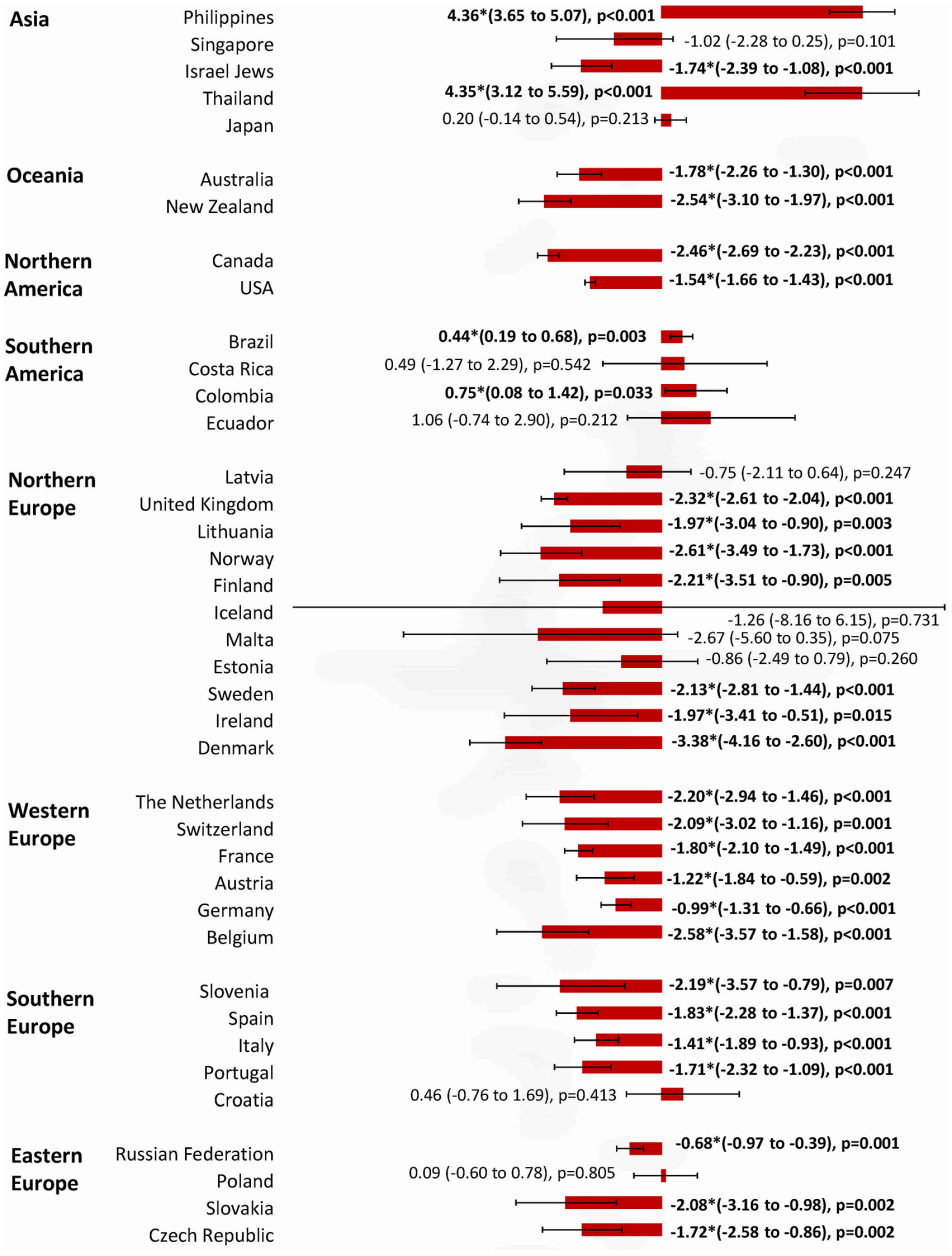
**The AAPC of the mortality of breast cancer in individuals aged 0-85+ years.**

**Figure 5:**
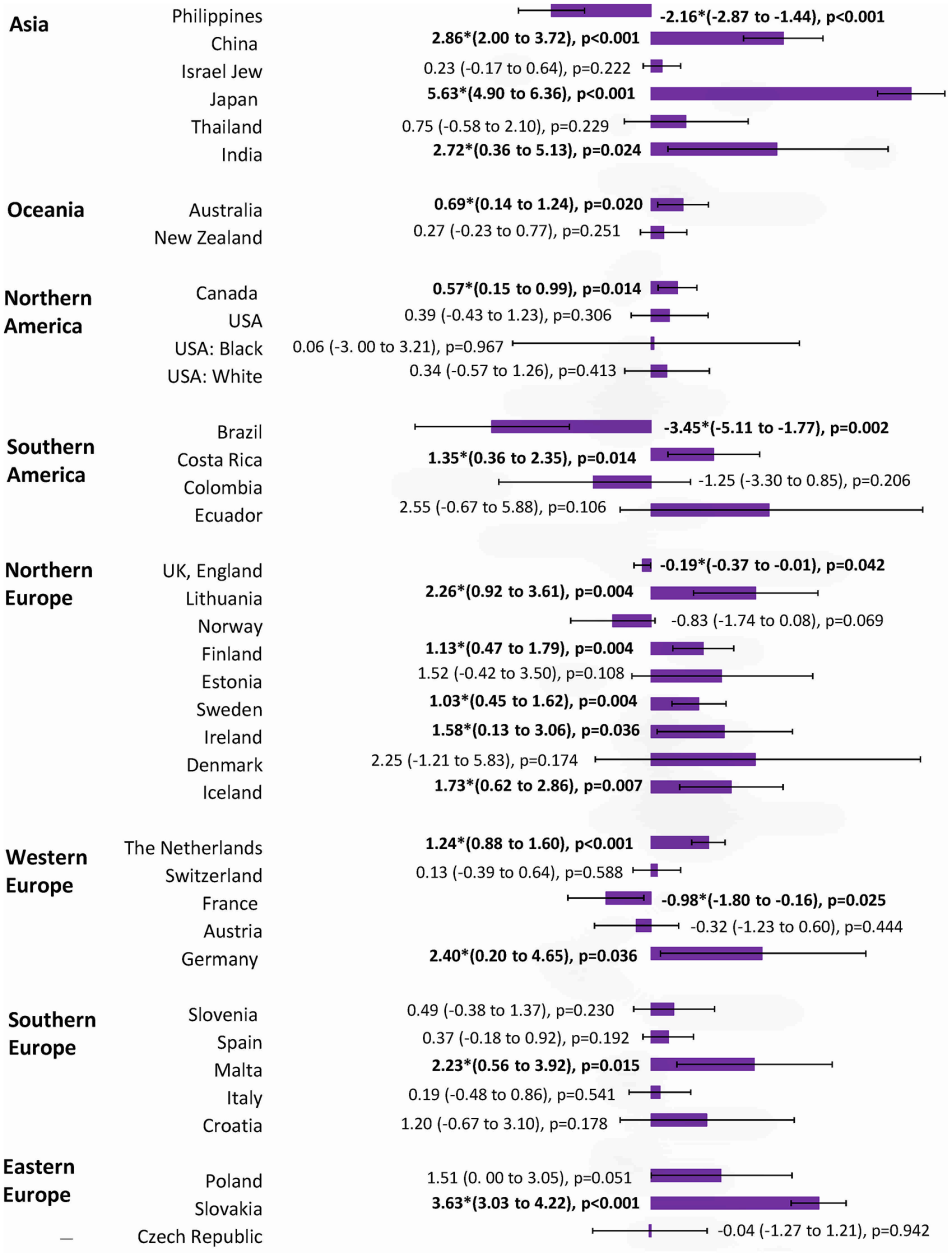
**The AAPC of the incidence of breast cancer in individuals aged ≥ 50 years.**

**Figure 6:**
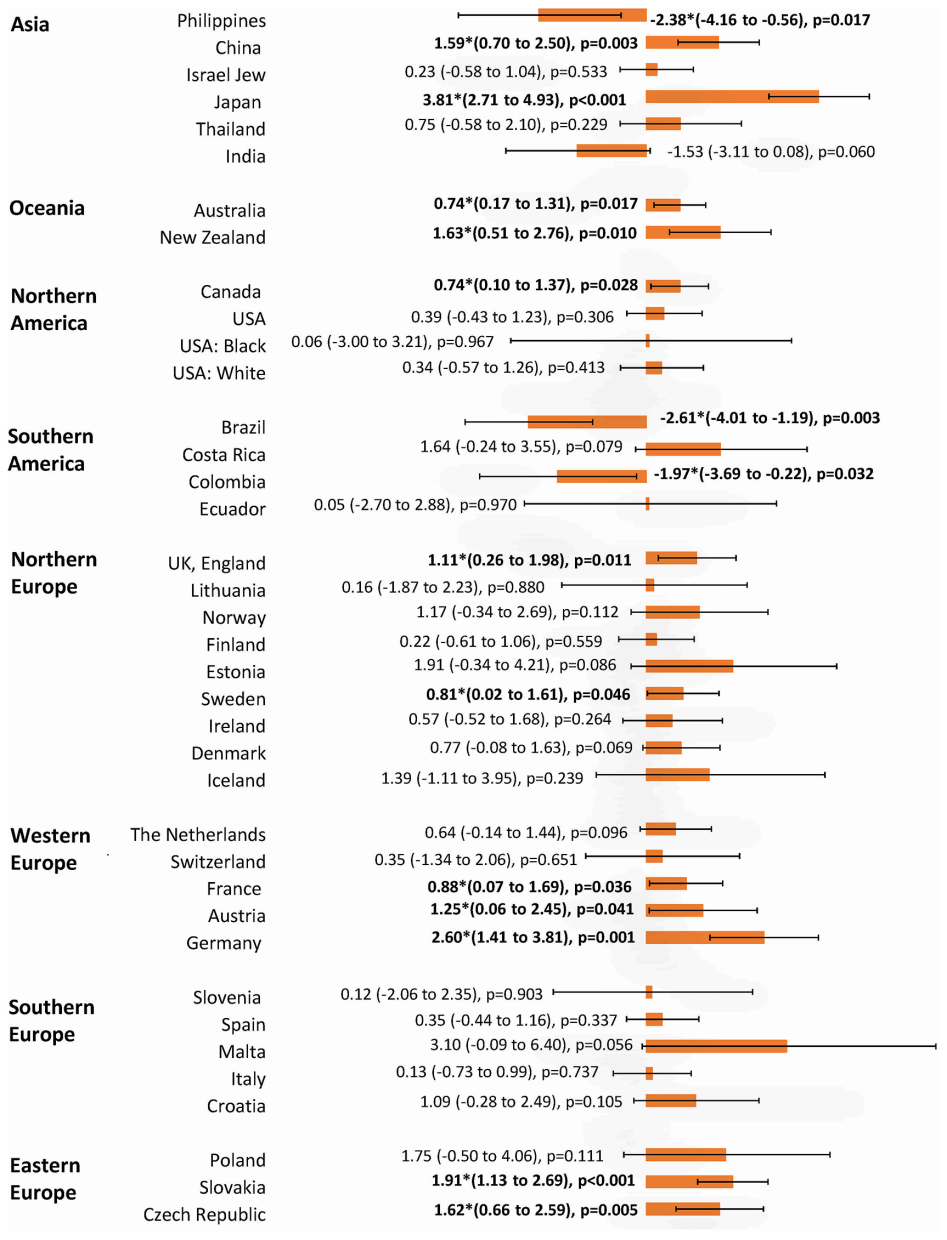
**The AAPC of the incidence of breast cancer in individuals aged < 50 years.**

**Figure 7:**
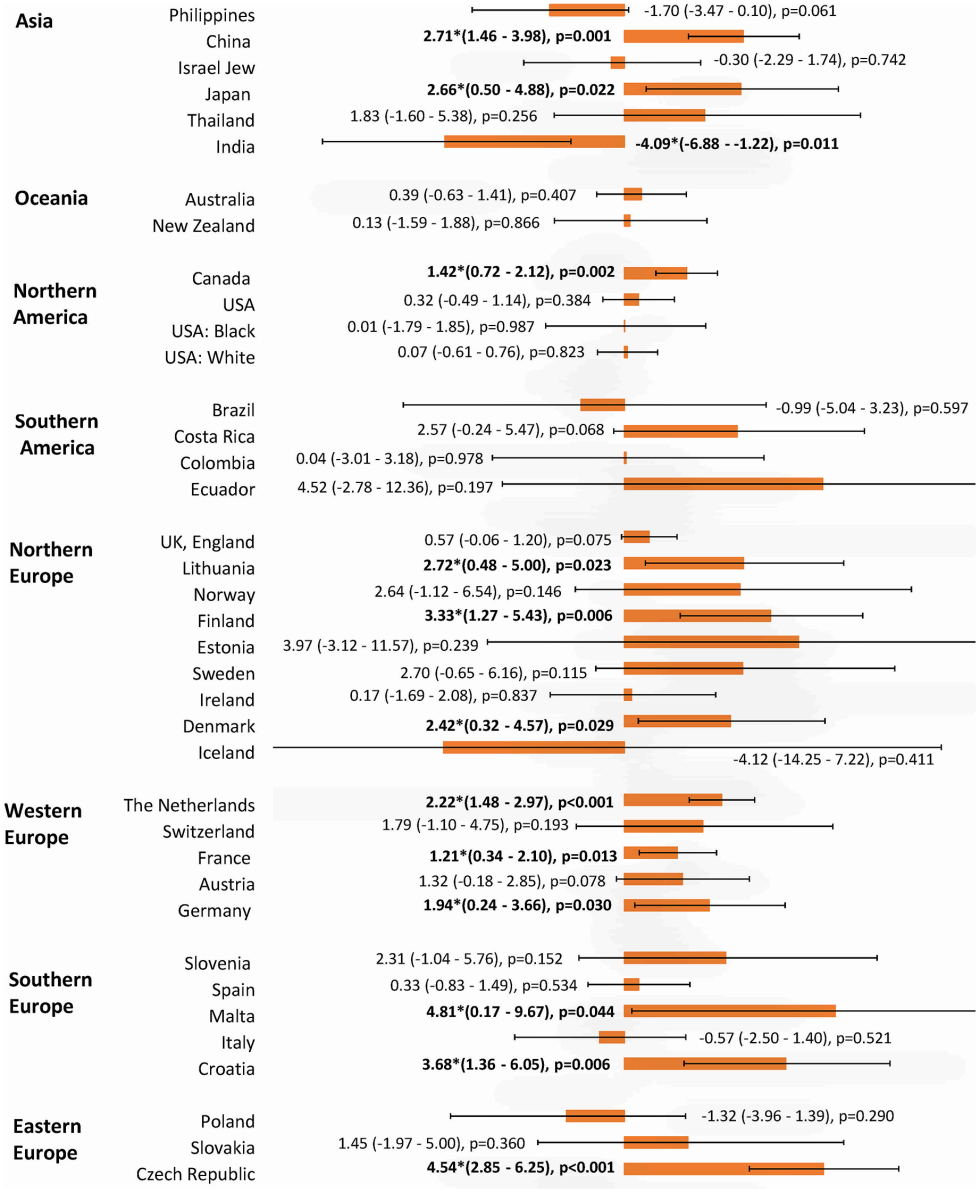
**The AAPC of the incidence of breast cancer in individuals aged < 40 years.**

The incidence and mortality trends of each country were shown in Supplementary Figure 1, and the corresponding findings from the joinpoint regression analysis were presented in Supplementary Figure 2. The changes in incidence and mortality trends were plotted in Figures 3–7.

### Incidence trend

A total of 16 countries had an increase in incidence and 20 countries reported stable trends (Figure 3). Out of all 16 countries with incidence rise, Japan (AAPC=4.83, 95% C.I 4.06, 5.61, p=0.001), Slovakia (AAPC=3.14, 95% CI 2.79, 3.49, p<0.001), Germany (AAPC=2.46, 95% CI 0.92, 4.02, p=0.006), Malta (AAPC=2.45, 95% CI 0.82, 4.11, p=0.008), and China (AAPC=2.35, 95% CI 1.57, 3.14, p<0.001) are among those with the highest incidence increase. Other countries with an increasing trend were Costa Rica, Canada, Thailand, Australia, New Zealand, Lithuania, Finland, Sweden, Iceland, the Netherlands, and Poland. Most increases in incidence trend were observed in European countries, Asia and Oceania. Only two countries, including Brazil (AAPC= -3.19, 95% CI -4.52, -1.85, p=0.001) and the Philippines (AAPC= -2.23, 95% CI -2.66, -1.80, p<0.001), showed a decreasing trend.

### Mortality trend

Among 39 countries, there were four countries which showed an increasing trend in mortality (Figure 4). These include the Philippines (AAPC=4.36, 95% CI 3.65, 5.07, p<0.001), Thailand (AAPC=4.35, 95% CI 3.12, 5.59, p<0.001), Colombia (AAPC=0.75, 95% CI 0.08, 1.42, p=0.033) and Brazil (AAPC=0.44, 95% CI 0.19, 0.68, p=0.003). Ten countries reported a stable trend in mortality (Costa Rica, Ecuador, Singapore, Japan, Latvia, Iceland, Malta, Estonia, Croatia, and Poland). A total of 25 out of 39 countries reported declining mortality rates, and 20 occurred in European countries and the Oceania. Among them, Denmark (AAPC= -3.38, 95% CI -4.16, -2.60, p<0.001), Norway (AAPC= -2.61, 95% CI -3.49, -1.73, p<0.001), Estonia (AAPC= -2.58, 95% CI -3.57, -1.58, p<0.001), Canada (AAPC= -2.46, 95% CI -2.69, -2.23, p<0.001), and the United Kingdom (AAPC= -2.32, 95% CI -2.61, -2.04, p<0.001) showed the most marked reduction in mortality rates.

### Incidence trend of breast cancer in younger vs. older individuals

The incidence of breast cancer increased in 16 out of 36 nations among individuals aged ≥50 years, with Japan (AAPC=5.63, 95% CI 4.90, 6.36, p<0.001), Slovakia (AAPC=3.63, 95% CI 3.03, 4.22, p<0.001), Thailand (AAPC=3.46, 95% CI 2.59, 4.34, p<0.001), China (AAPC=2.86, 95% CI 2.00, 3.72, p<0.001), India (AAPC=2.72, 95% CI 0.36, 5.13, p=0.024), and Germany (AAPC=2.40, 95% CI 0.20, 4.65, p=0.036) having the highest increase (Figure 5). Four countries which showed a decrease were Brazil, USA (white), the Philippines, and France. Other 16 countries showed a stable trend. As for the populations aged <50 years, the increase was observed in 12 countries, which were comparable to the older (Figure 6). These included Japan (AAPC=3.81, 95% CI 2.71, 4.93, p<0.001), Germany (AAPC=2.60, 95% CI 1.41, 3.81, p=0.001), Slovakia (AAPC=1.91, 95% CI 1.13, 2.69, p<0.001), New Zealand (AAPC=1.63, 95% CI 0.51, 2.76), p=0.010), the Czech Republic (AAPC=1.62, 95% CI 0.66, 2.59, p=0.005) and China (AAPC=1.59, 95% CI 0.70, 2.50, p=0.003). Three countries showed a decrease (Brazil, Colombia, and the Philippines). The remaining 21 countries showed a stable trend. Among those younger than 40 years, 12 countries showed an increasing incidence, including Malta (AAPC=4.81, 95% CI 0.17, 9.67, p=0.044), Czech Republic (AAPC=4.54, 95% CI 2.85, 6.25, p<0.001), Croatia (AAPC=3.68, 95% CI 1.36, 6.05, p=0.006), Finland (AAPC=3.33, 95% CI 1.27, 5.43, p=0.006), Lithuania (AAPC=2.72, 95% CI 0.48, 5.00, p=0.023) and Denmark (AAPC=2.42, 95% CI 0.32, 4.57, p=0.029) (Figure 7). India is the only country that reported a decreasing incidence of breast cancer (AAPC= -4.09, 95% CI -6.88, -1.22, p=0.011). Other 23 countries showed a stable trend.

## DISCUSSION

### Major findings

This study found that the highest incidence of breast cancer was found in Western countries and nations with high or very high HDI, whilst its mortality rate was greater in nations with low HDI. A large number of countries, namely European nations, Asia and Oceania, showed a rapidly rising incidence whilst most countries reported a decline in mortality trend. The incidence increase was also observed in younger individuals in a substantial proportion of countries.

### Relationship with literature and explanations of findings

Our study shows that 16 countries had increases in the incidence of breast cancer and those countries with higher increase in AAPC were developed countries, such as Japan and Germany, or countries in Asia and Oceania with rapid economic development in the past several decades. The higher incidence observed could be related to the genetic factors for different ethnicities in different countries. Generally, evidence shows that the incidence of breast cancer is strongly correlated with HDI [22]. The increase in incidence in these countries could be attributable to the increasing prevalence of risk factors in high HDL countries, such as obesity, physical inactivity, and alcohol consumption. The increase in incidence may also be related to a better access to healthcare service in these countries, so that breast cancer can be detected earlier. This phenomenon also explains the observation of the declining trends in mortality among most countries. Early detection and advanced treatment can greatly increase the survival rate and life expectancy of bread cancer patients. A study by Ghoncheh et al. shows that the increasing incidence of the breast was not necessarily related to increased death rate [22], especially in countries with high HDI. Patients with higher socioeconomic status are generally diagnosed earlier and have longer survival [23].

A notable observation in our study is that an incidence increase was observed in younger individuals in a substantial proportion of countries. This finding is compatible with the results of previous studies [24]. The exact reason for this phenomenon remains unknown. However, several lifestyle factors may contribute to the increase, and these may include the growing prevalence of physical inactivity, dietary consumption of high animal fat, and excess body weight among younger women. Our team have previously conducted a study of the epidemiological trends of colorectal cancer among 39 nations and observed that its incidence continued to grow in the younger populations [18]. As breast cancer and colorectal cancer shared a certain proportion of risk factors, one possible explanation of the epidemiological trend is the increasing prevalence of obesity in the younger population. A substantial number of studies have observed the association between excess body weight and the risk of breast cancer [25]. It has been postulated that excess body weight may lead to breast cancer via altered steroid metabolism which results in the elevated level of estrogen targeting breast and endometrium [25]. A meta-analysis found that excess body weight was associated with an increased risk of breast cancer (RR=1.25) for postmenopausal females [26]. Recent WHO report indicated that the prevalence of obesity in children had increased from 4% to 18% during 1975-2016 [27]. This could be partially driving the increase in obesity in the younger population. Central obesity, compared with general obesity, may be associated with a higher risk of breast. Pooled results from cohort studies suggested a 39% higher risk of breast cancer among females with central obesity [28]. Our team have recently performed a meta-analysis on the global epidemiological trend of central obesity in more than 13 million individuals. A more drastic increase was found among the younger population aged 15-40 years (16.3% to 33.9%) when compared with the older (43.6% to 57.9%) [29]. This may have shifted obesity-related cancers to younger. This trend is expected to increase further in the future provided the inevitable ageing and lifelong exposure to obesity-associated risk factors. Some reproductive factors may also play a role as part of the incidence rise could be attributed to premenopausal breast cancer. In many developed nations, young women tend to use oral contraceptives for a longer period and may have fewer children and start having kids later in life. This could bear a significant effect on female sex hormones. Moreover, younger women often tend to present with late-stage breast cancer than their older counterparts, which further explains the poorer prognosis of cancer. Thus, closer monitoring and follow-up for younger breast cancer patients are necessary.

### Strengths and limitations

We have presented and analysed the most up-to-date incidence and mortality of breast cancer, but several limitations should be addressed. First, there is a possibility of under-reporting of cancer diagnoses particularly in countries with low HDI, and this could introduce bias in the evaluation of cancer statistics [30]. In regional cancer registries, the incidence and mortality figures could be underestimated due to a lack of local facilities, less well-developed reporting infrastructure, and lower precision of data when compared with national registries. Also, in nations where figures were estimated from a single cancer registry that recorded data in more urbanized, resource-privileged areas, the rates representing the countries could be biased especially if they have a different mix of urban and rural populations. Besides, only a certain proportion of the world's countries presented high-quality incidence and mortality data. The quality of mortality data in terms of coverage, accuracy, and completeness also varies among countries [31], although the purpose of this study is not to compare mortality rates among them. Also, these epidemiological data could be confounded by detection and attribution biases, such as that introduced by inaccuracy for death certificates and differences in case ascertainment or reporting mechanisms across different countries. Finally, the latest data provided by GLOBOCAN, CI5 and the WHO mortality database were updated by 2018 despite our best efforts to analyze the most recent data - so further updates are needed to reflect the most recent temporal trend.

### Implications and conclusion

In summary, the incidence rates of breast cancer increased in most countries whilst the mortality rates declined in most nations. Breast cancer incidence was found to increase in a substantial number of countries in the younger population. With the ageing and growing population, and the increasing prevalence of many risk factors, a further substantial rise in the incidence of breast cancer could be expected. Therefore, more health care resources will be needed for the treatment of patients with breast cancer, and particular in more resource-deprived countries. In all nations, public health strategies should be formulated and implemented through health education and promotion of evidence-based screening program. However, the strategies need to be tailored by different countries according to the burden of the diseases, available medical resources, culture and acceptance of the screening tools, and related health regulations. Besides, it is also important to evaluate the cost-effectiveness of such interventions in the settings of individual countries. Future studies should explore the underlying reasons for these epidemiological trends and investigate global health actions that could effectively reduce the healthcare burden attributable to breast cancer.

## MATERIALS AND METHODS

### Source of data

The present study employed the same analysis approach of our previous studies on prostate [32], liver [33]; pancreatic [34], bladder [35], and colorectal cancer [18] [36]. Figures of breast cancer incidence and mortality in 2018 were extracted from the GLOBOCAN database [1]. Each country’s data were matched with its HDI based on the United Nations Human Development Report [37]. The classification of HDI was defined as low (≤ 0.534), medium (0.534–0.710), high (0.710–0.796) and very high (>0.796) [37]. In our time trend analysis, we retrieved figures from a various database: Cancer Incidence in Five Continents series I-X (CI5) [38], the Nordic Cancer Registries (NORDCAN) [39], and the Surveillance, Epidemiology, and End Results Program (SEER) [40]. These databases provided high-quality cancer statistics by domestic registries worldwide. In the mortality trend analysis, we retrieved the statistics from the World Health Organization (WHO) [41], which are derived from death certificates. The *CI5* consists of population-based cancer registries globally and retrieve information on incidence by recording the occurrence of each cancer case in a pre-determined period [38]. It covered approximately 15% of the worldwide population using cancer registries of high-quality. It included indexes such as the rate of cancer cases reported microscopically, the proportion of cancer deaths registered, and the percentage of cancer cases registered to assess the quality of information by period, gender, and primary cancer sites. The *NORDCAN* database was searched for the most updated incidence/mortality on breast cancer in Finland, Norway, Sweden, Denmark, Iceland, Greenland, and Faroe Islands [39]. The *SEER* database was used for the United States (all races, White, and Black), as it provided the most updated cancer data [40]. We identified breast cancer in the database search using the International Classification of Diseases and Related Health Problems-10th Revision codes (ICD-10). The WHO mortality database was retrieved to obtain the mortality figures for other countries [41]. The causes of death data were from civil registration systems across different countries. Mortality was recorded at a local civil registry with information on the cause of death. The information was collected by the health authority and reported to the WHO annually. Only medically certified mortality cases were reported. All incidence/mortality rates in the database were presented as age-standardized rates (ASRs) adjusted for the Segi–Doll world standard population. We extracted the age-standardized rate per 100,000 (ASR) in countries across the five continents from 1975-2016. Supplementary Table 2 presented detailed descriptions of data sources and timeframe of the present study. This study obtained approval by the Survey and Behavioural Research Ethics Committee of the Chinese University of Hong Kong. All methods and procedures adhered to the relevant guidelines and regulations and there was no identifying information published.

### Statistical analysis

Jointpoint regression analysis [42] was used to generate breast cancer incidence and mortality trends. The ASR trend of each country was presented by the joined straight lines [42]. In our analysis, the ASRs were logarithmically transformed and the standard errors were computed using binomial approximation. The analysis options included up to three joinpoints. To ascertain the direction and magnitude of changes in incidence and mortality, the Average Annual Percent Changes (AAPCs) with their 95% confidence interval (CI) were calculated for the most recent decade. By using joinpoint trend analysis software, the AAPC was subsequently computerized as a geometrically weighted average of the generated Annual Percent Changes (APCs). In each segment, the weights and lengths were equivalent within the specified time interval [43]. When deciding the timeframe for examining temporal trend changes, we followed the common practice in epidemiologic studies on other cancers [32, 36, 44], which is the most recent 10 years. AAPC with 95% CI greater and lower than zero indicated an increasing and decreasing trend, respectively. If the AAPC with 95% CI overlaps with zero, it would be considered as a stable trend [32, 36, 44]. We also compared the epidemiological trend of breast cancer between younger population (< 50 years) and older population (≥ 50 years) for individual countries. To further confirm the trends of breast cancer among younger population, we performed an additional analysis on the trends of breast cancer among population aged < 40 years.

## Supplementary Material

Supplementary Figures

Supplementary Tables
