# Supplementary material for: Global incidence and mortality of breast cancer: a trend analysis
Source: Aging (Albany NY). 2021 Feb 11;13(4):5748–803. doi: 10.18632/aging.202502 (PMC7950292; doi:10.18632/aging.202502)
Supplement: Supplementary Figures [file aging-13-202502-s001.pdf]

SUPPLEMENTARY FIGURES

Latin America & the Caribbean

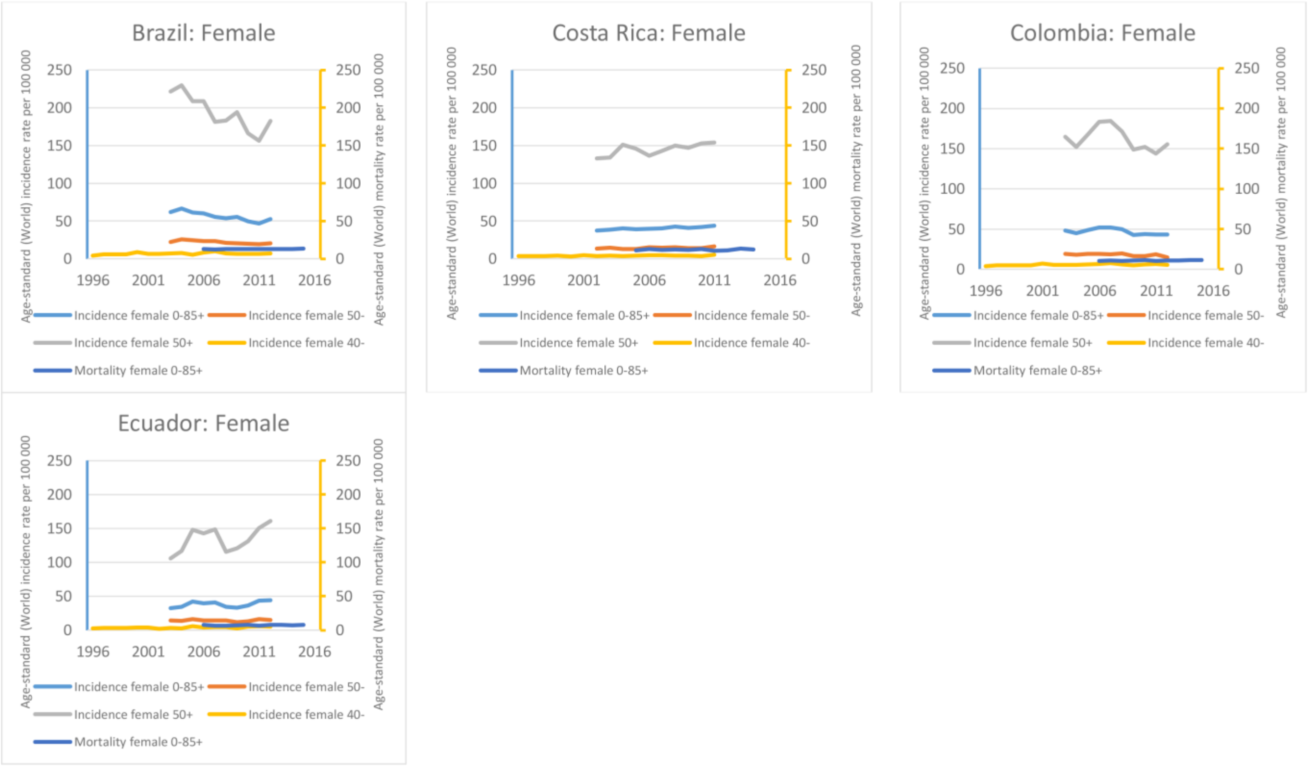

Northern America

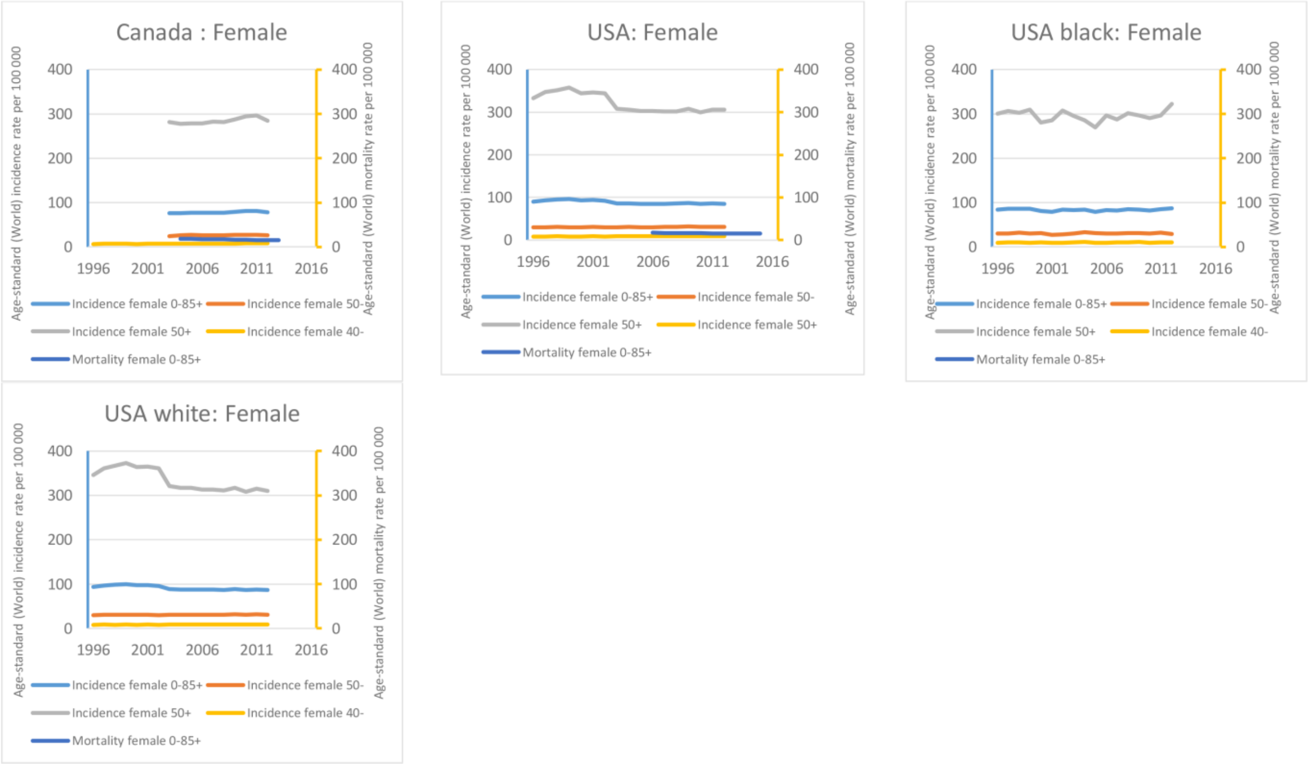

Asia

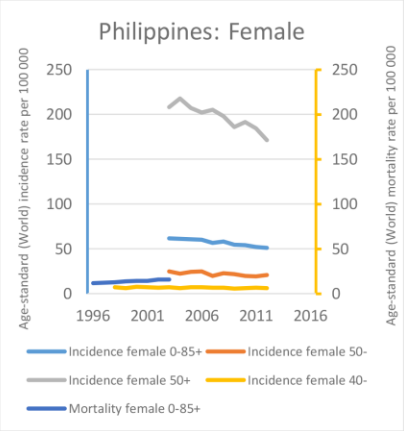

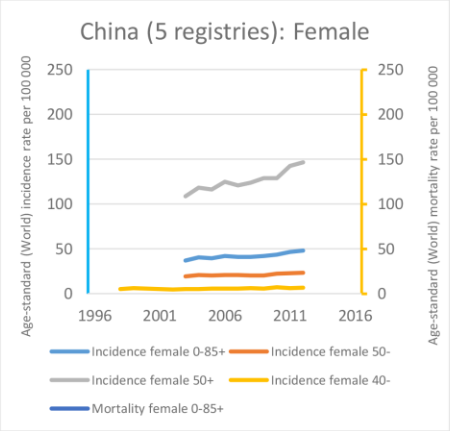

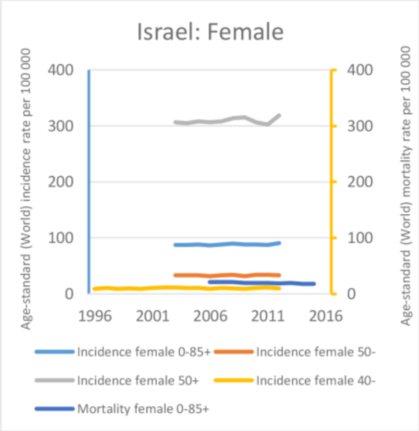

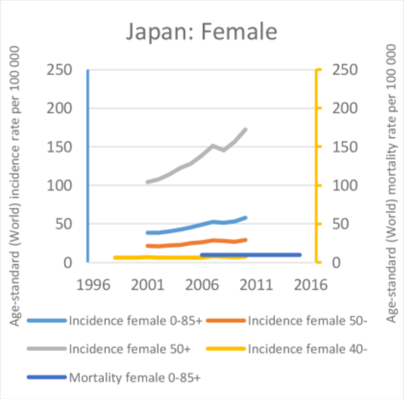

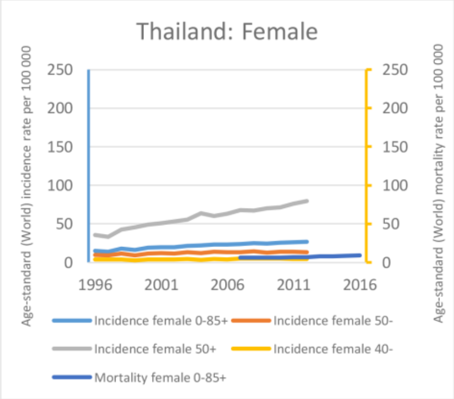

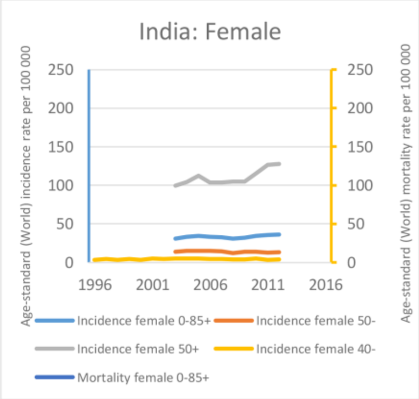

Oceania

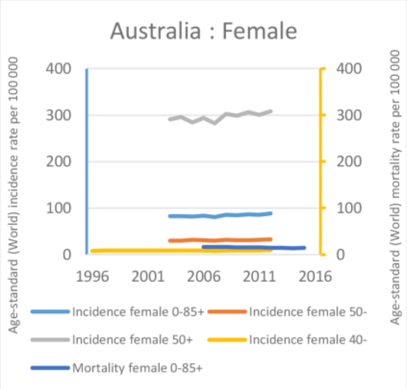

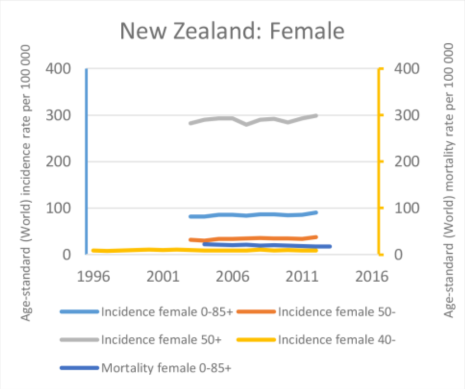

Northern Europe

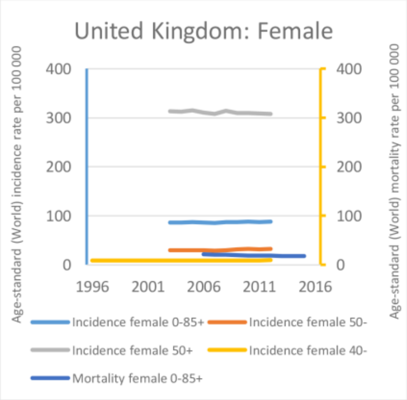

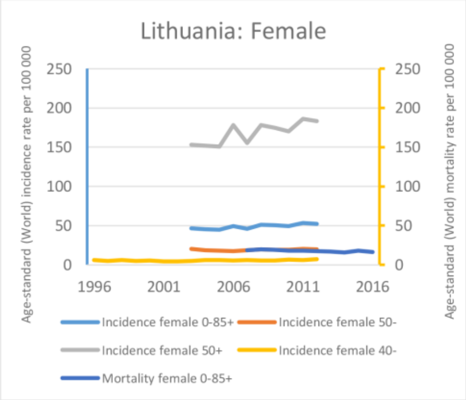

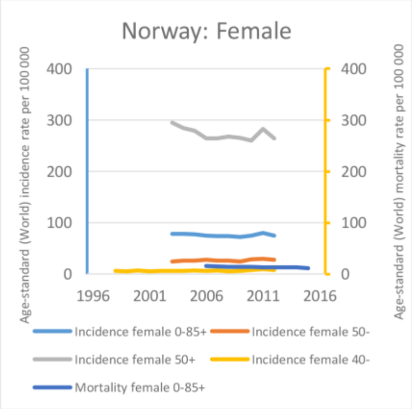

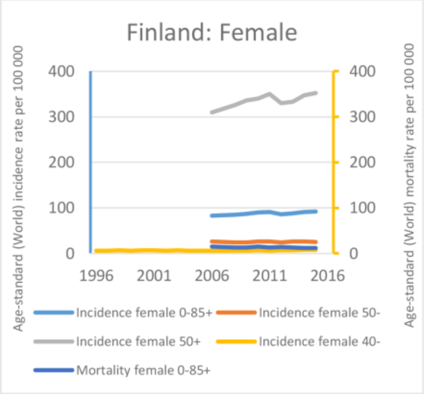

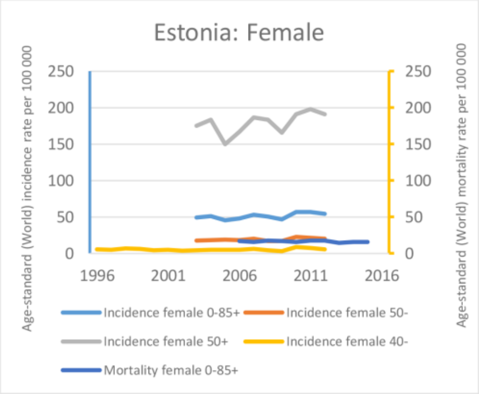

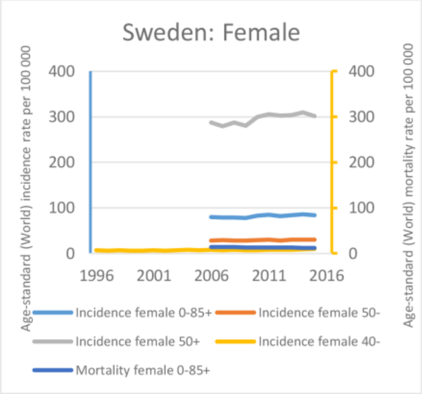

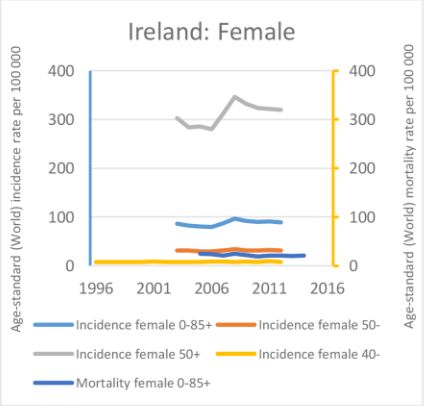

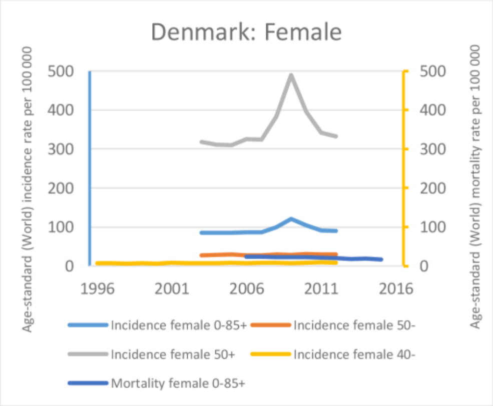

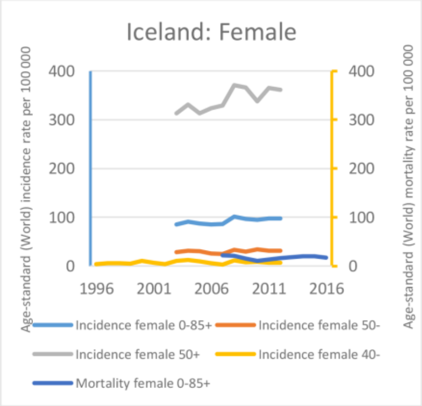

Western Europe

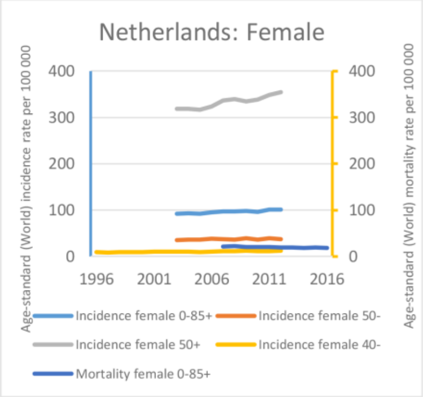

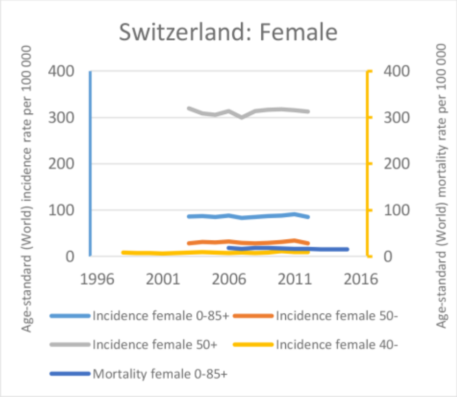

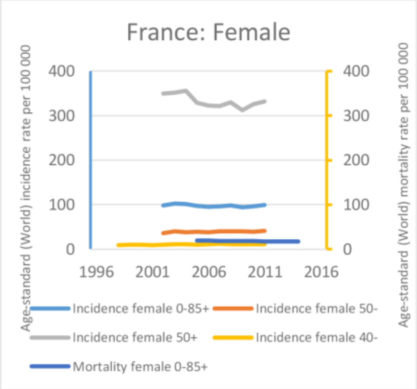

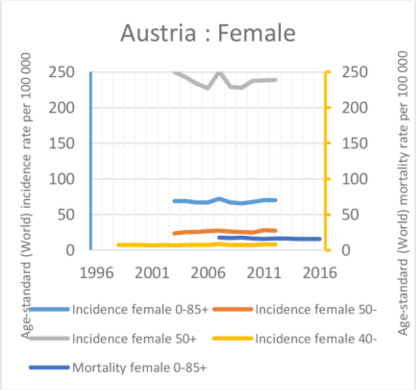

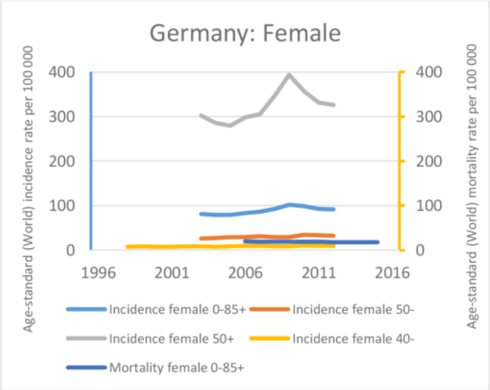

Southern Europe

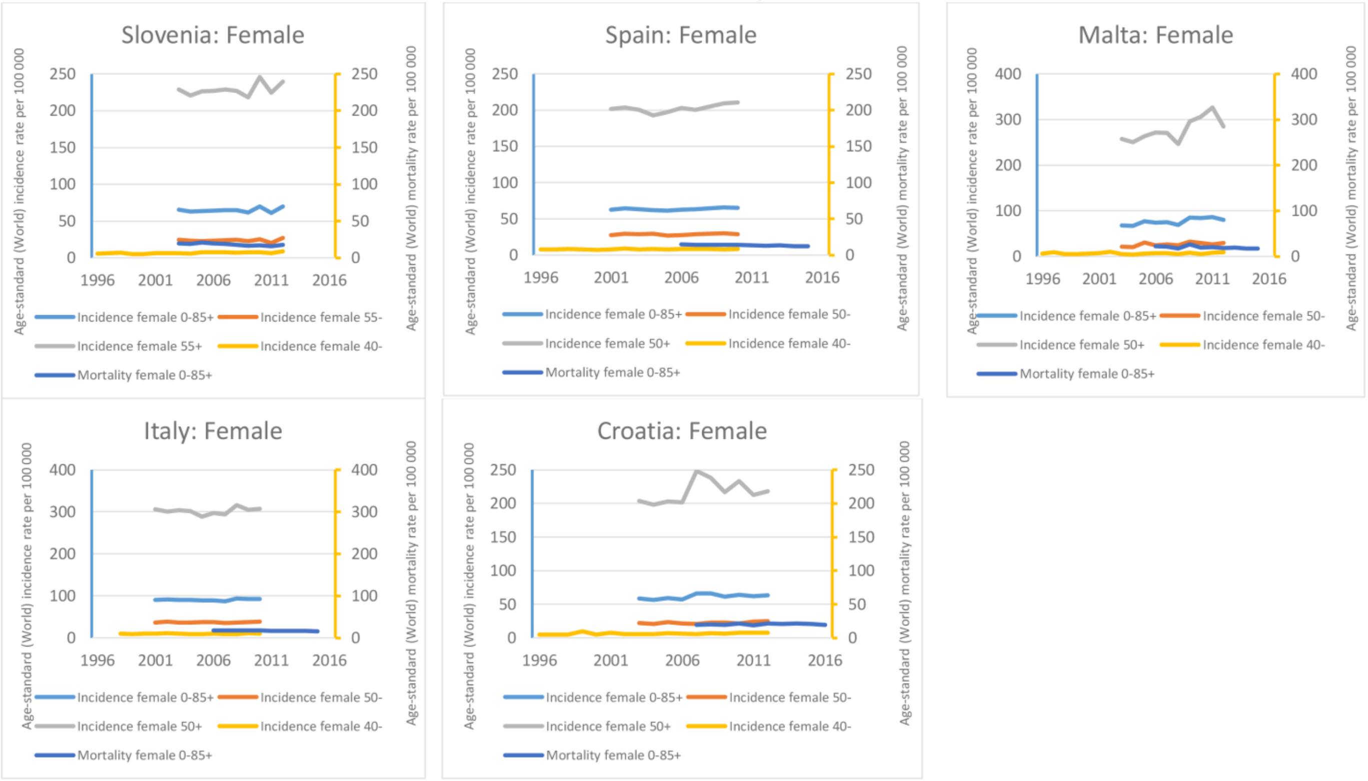

Eastern Europe

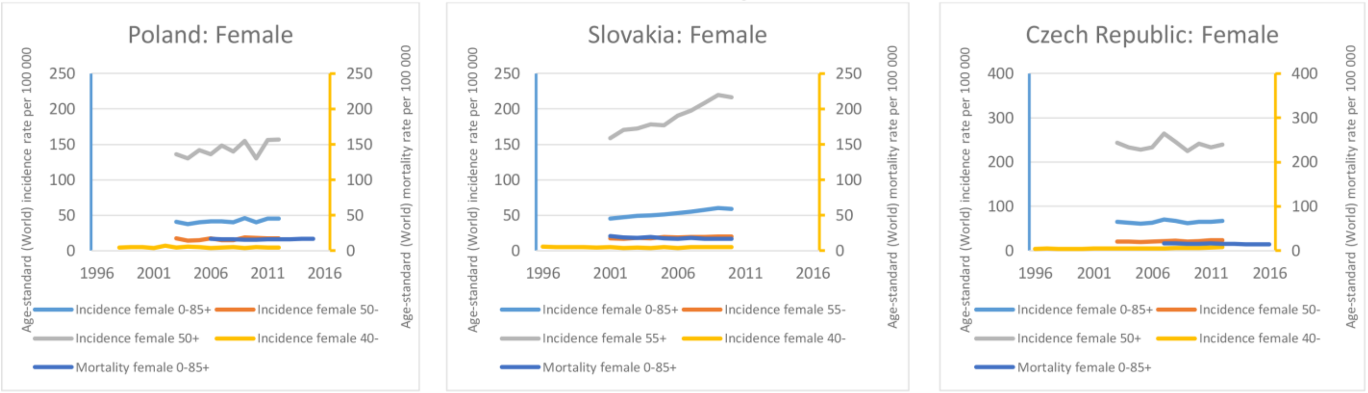

Supplementary Figure 1. The incidence/mortality trends of breast cancer by country.

Latin America & the Caribbean

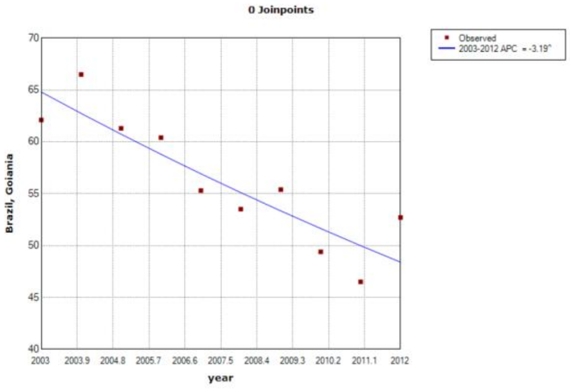

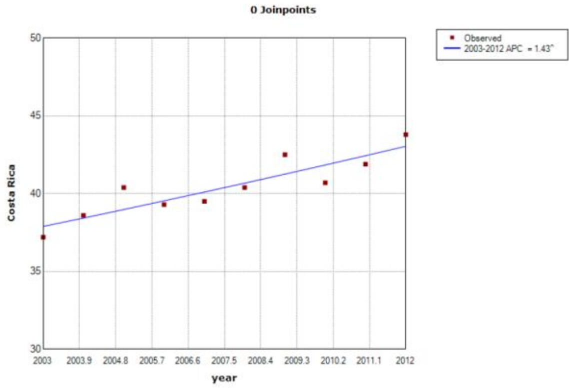

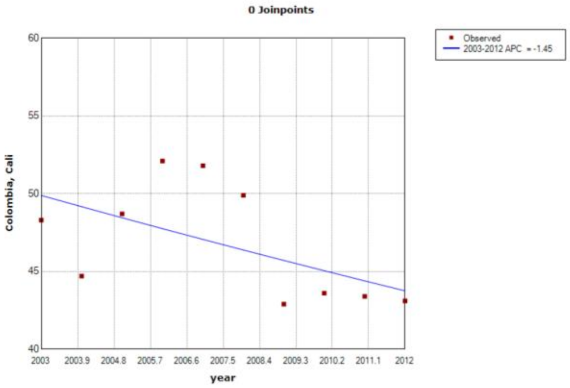

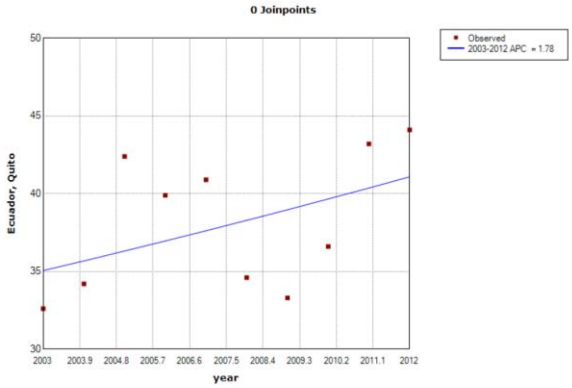

Northern America

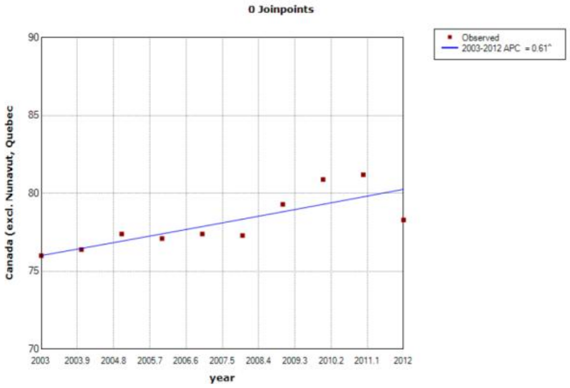

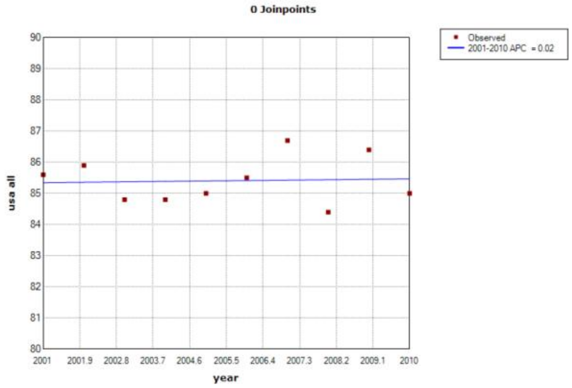

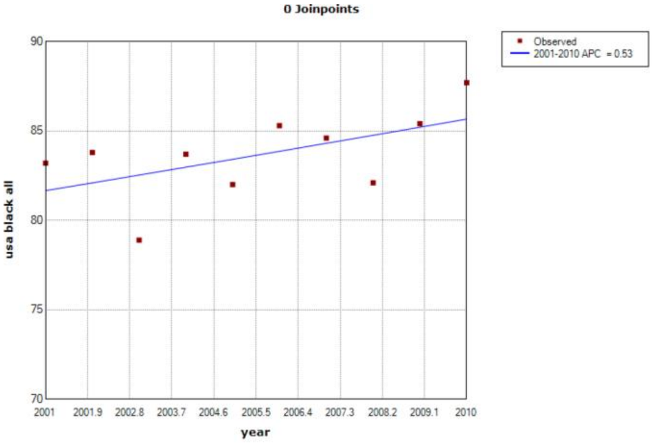
0 Joinpoints
Observed
2001-2010 APC = 0.53
usa black all
year

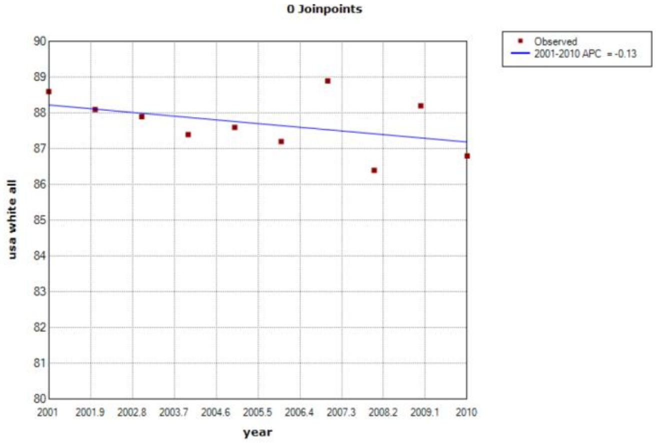
0 Joinpoints
Observed
2001-2010 APC = -0.13
usa white all
year

Asia

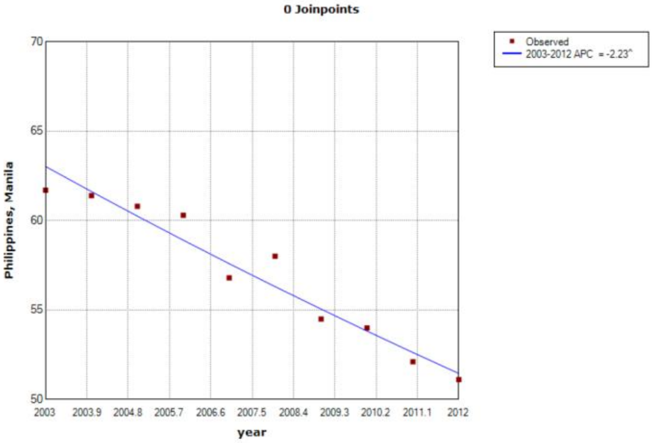
0 Joinpoints
Observed
2003-2012 APC = -2.23^
Philippines, Manila
year

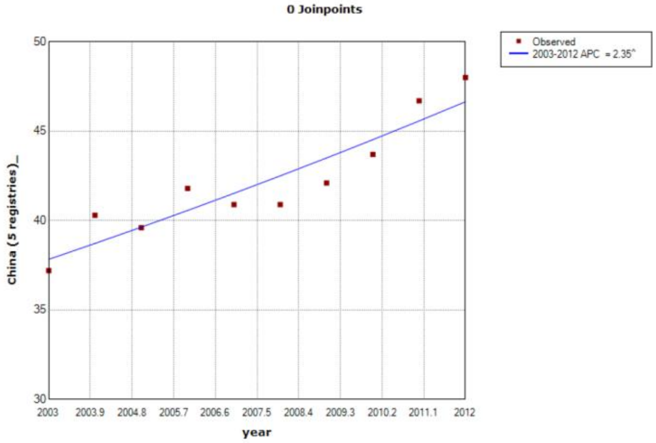
0 Joinpoints
Observed
2003-2012 APC = 2.35^
China (5 registries)_
year

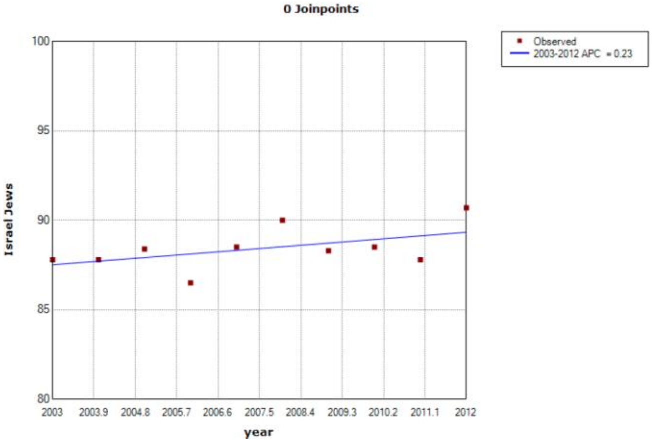
0 Joinpoints
Observed
2003-2012 APC = 0.23
Israel Jews
year

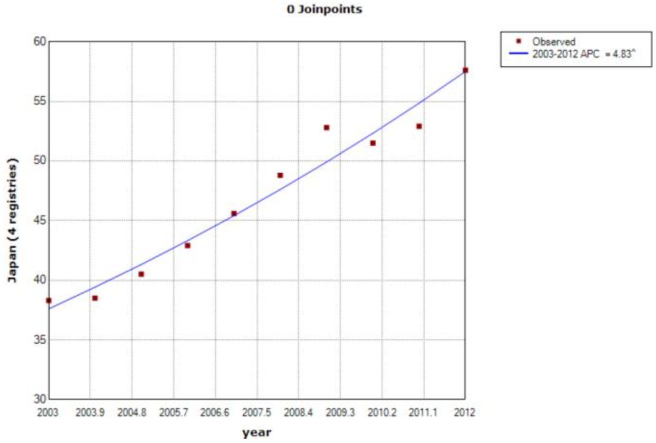
0 Joinpoints
Observed
2003-2012 APC = 4.83^
Japan (4 registries)
year

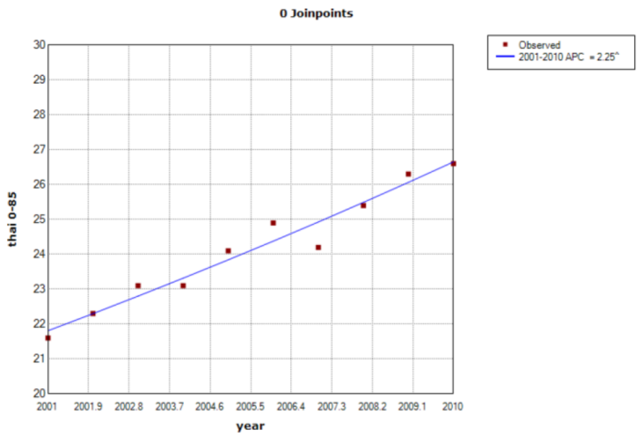

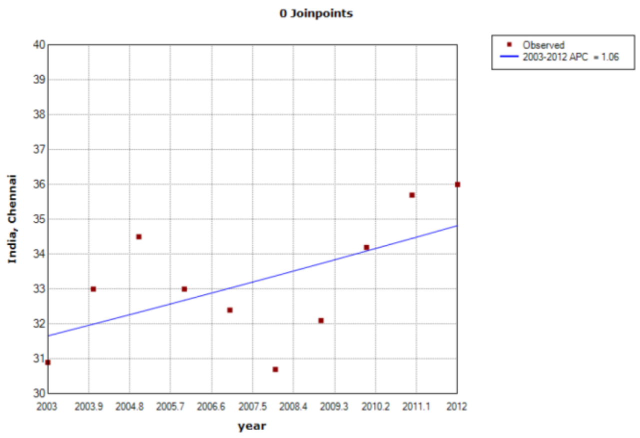

Oceania

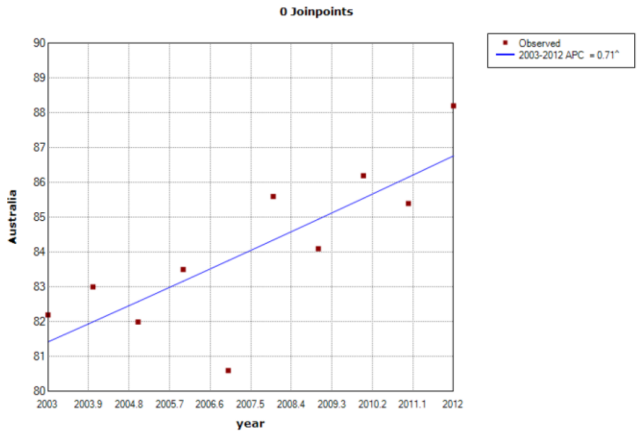

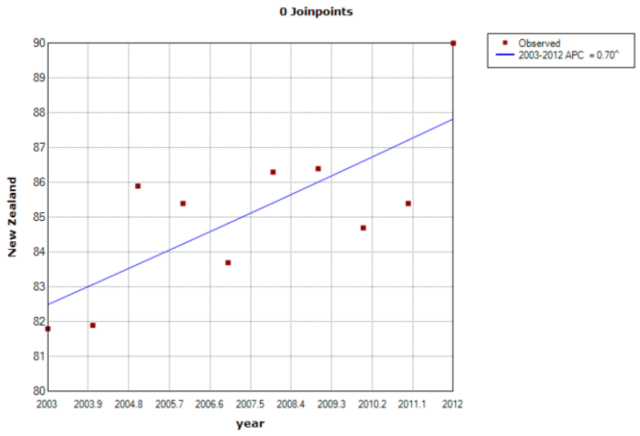

Northern Europe

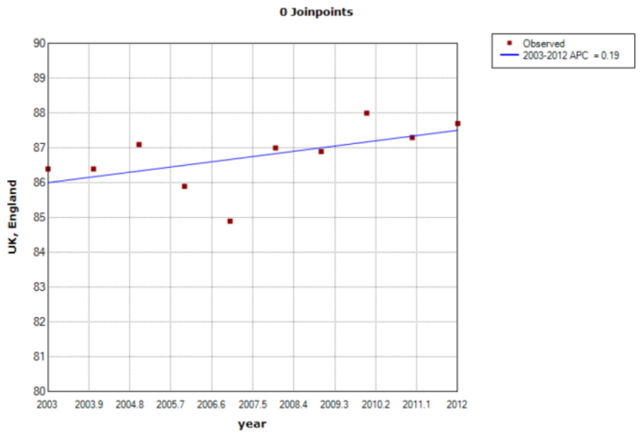

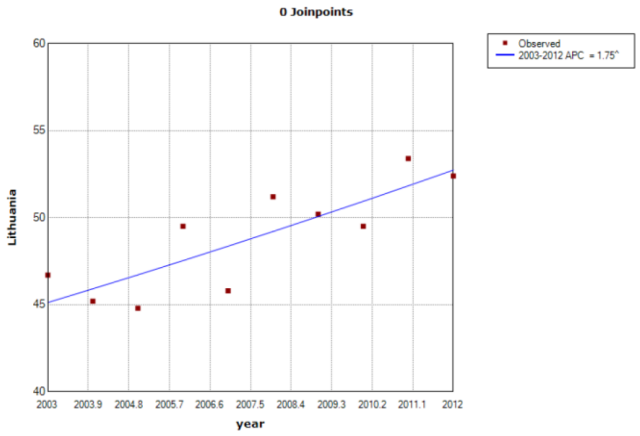

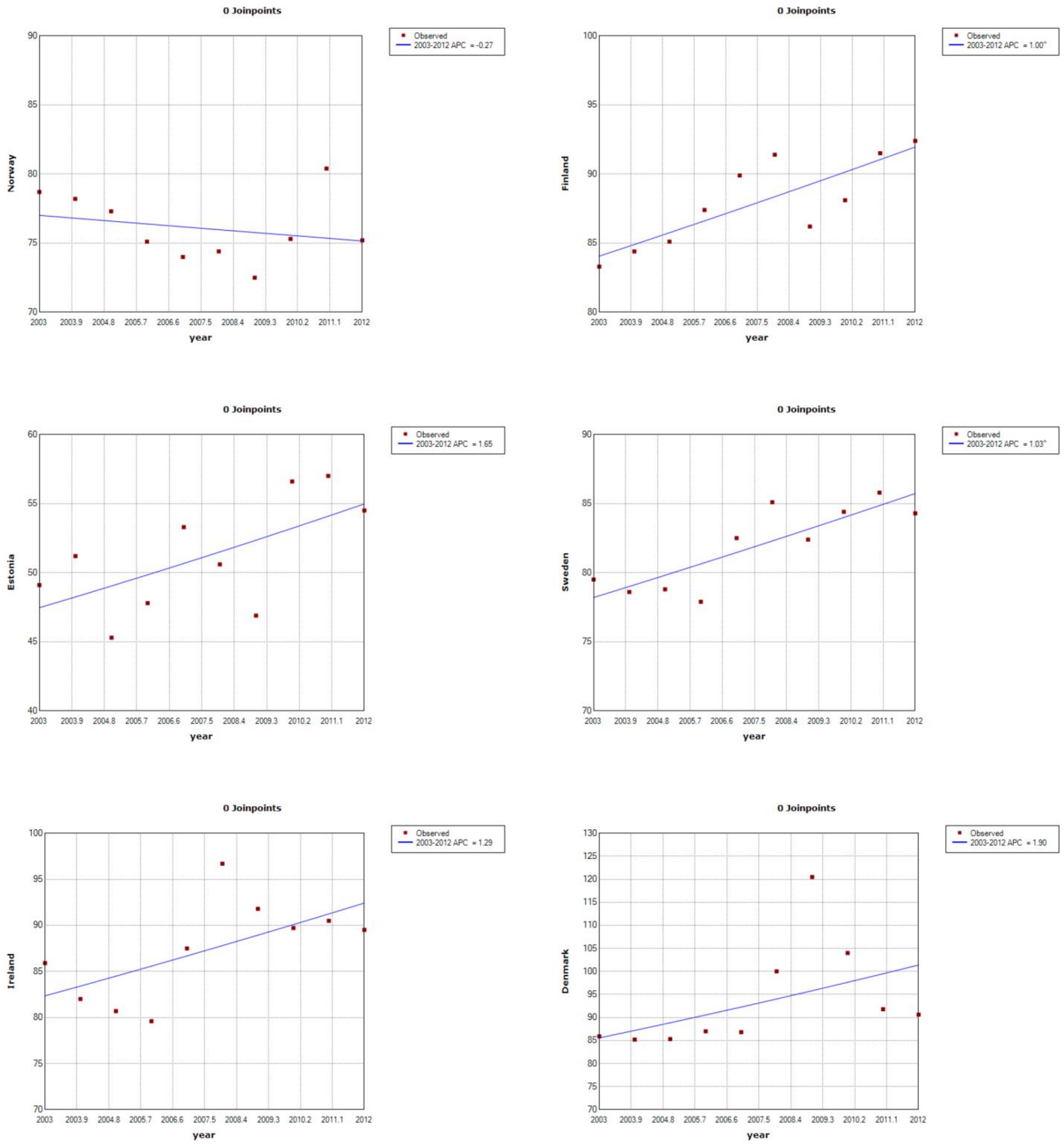
0 Joinpoints
Observed
2003-2012 APC = -0.27
Norway
year
0 Joinpoints
Observed
2003-2012 APC = 1.00^
Finland
year
0 Joinpoints
Observed
2003-2012 APC = 1.65
Estonia
year
0 Joinpoints
Observed
2003-2012 APC = 1.03^
Sweden
year
0 Joinpoints
Observed
2003-2012 APC = 1.29
Ireland
year
0 Joinpoints
Observed
2003-2012 APC = 1.90
Denmark
year

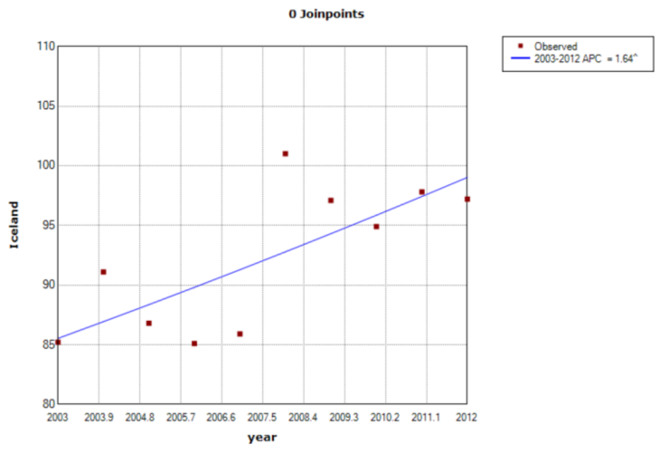

Western Europe

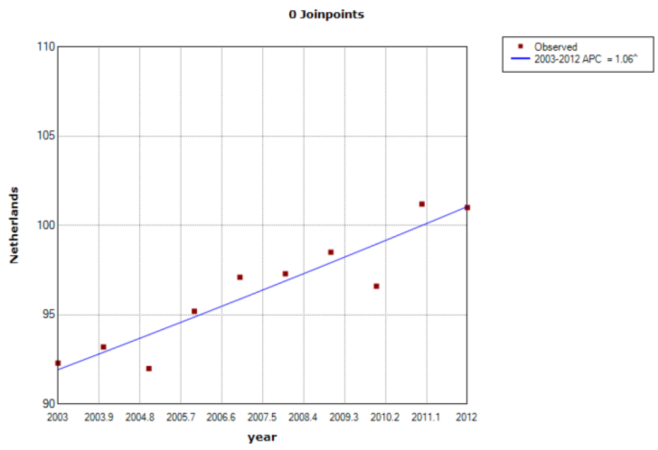

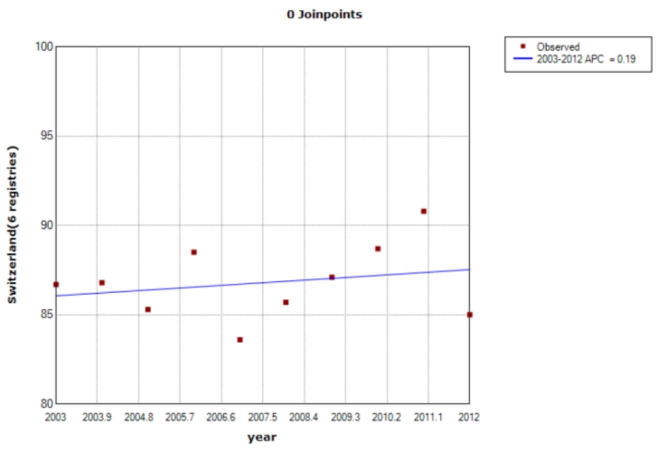

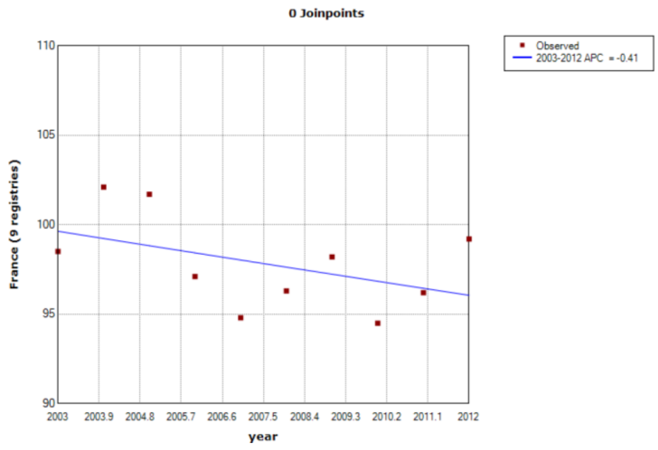

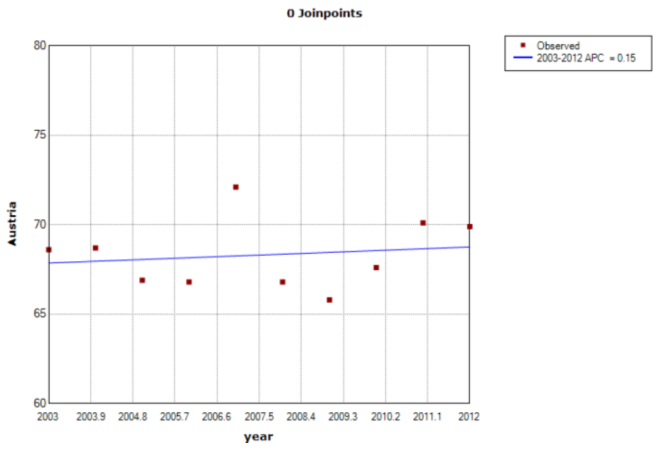

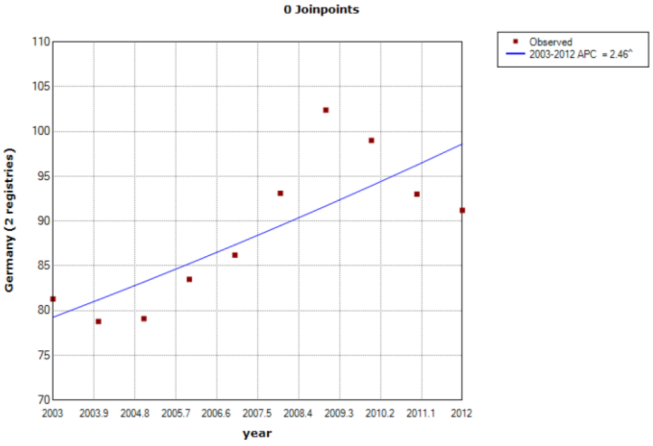

Southern Europe

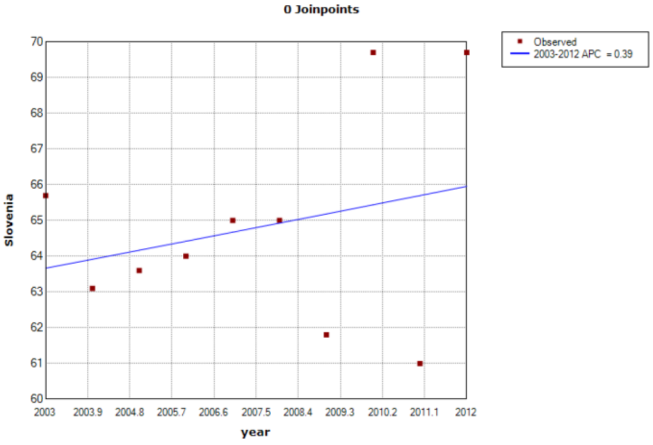

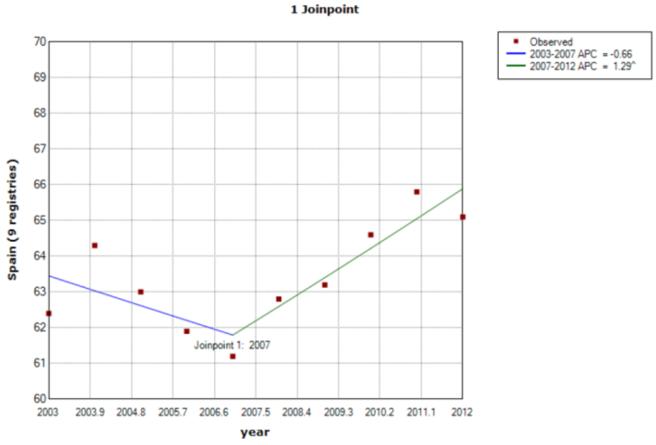

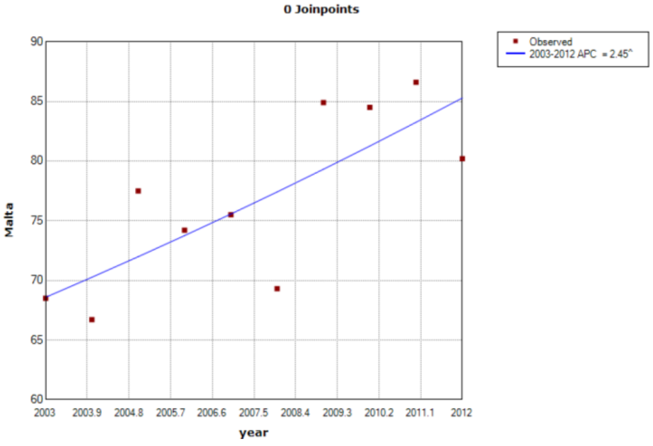

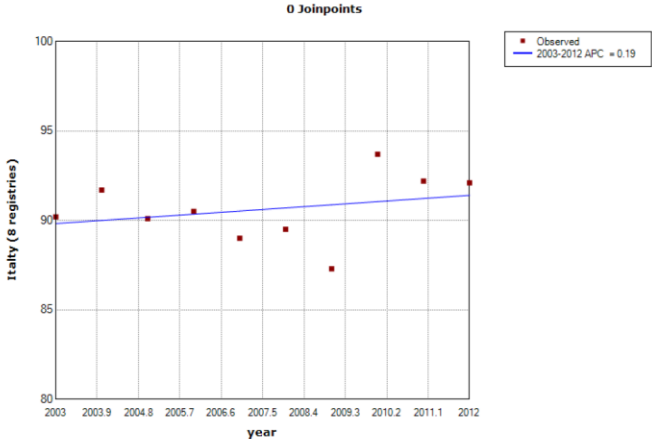

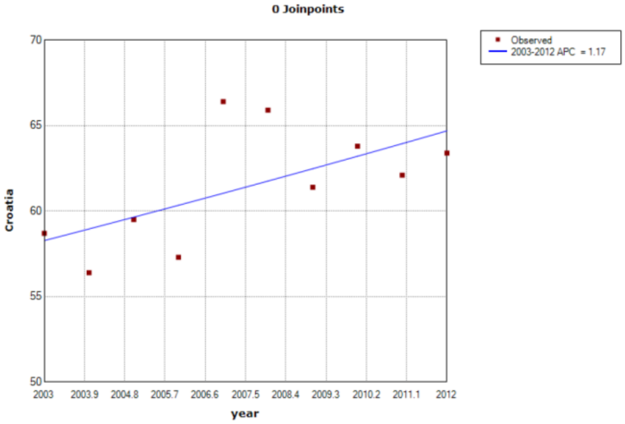

Eastern Europe

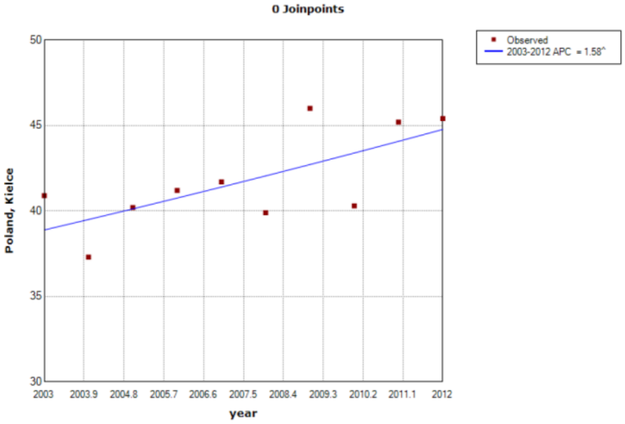

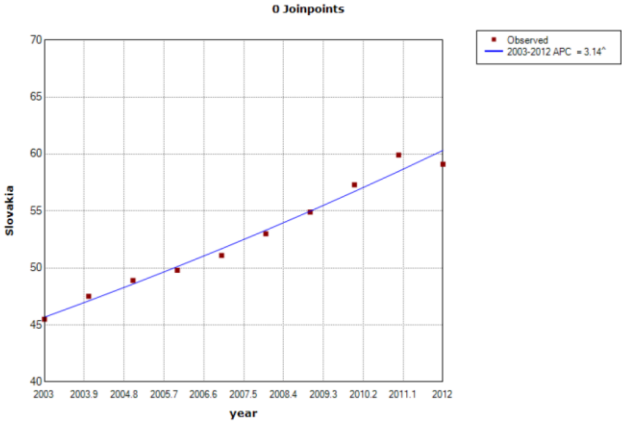

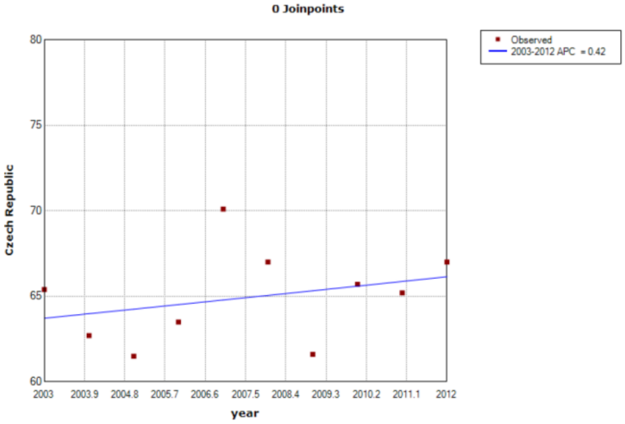

Latin America & the Caribbean

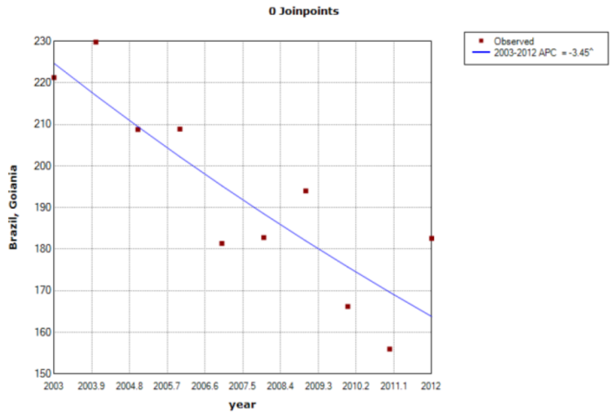

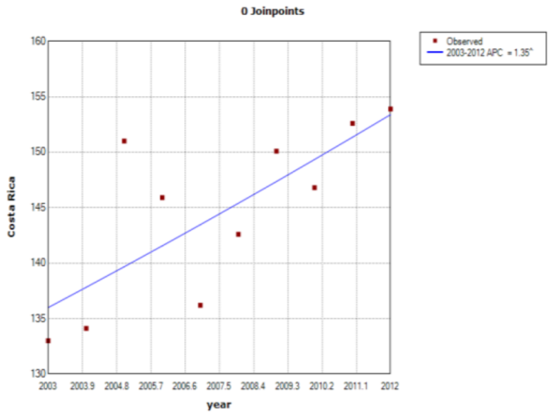

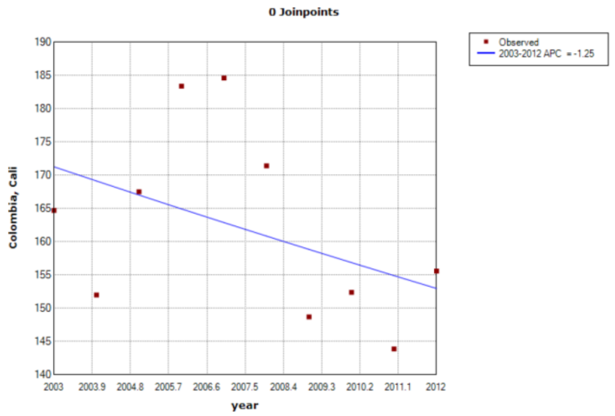

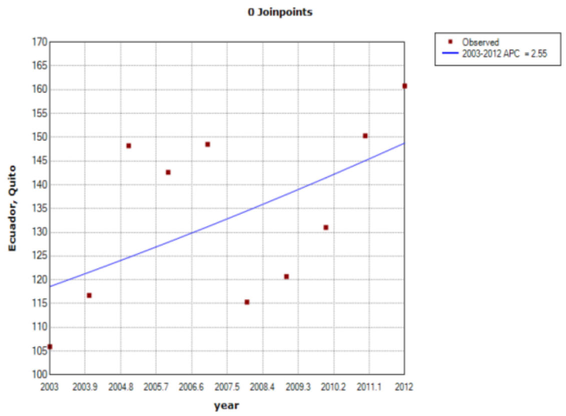

Northern America

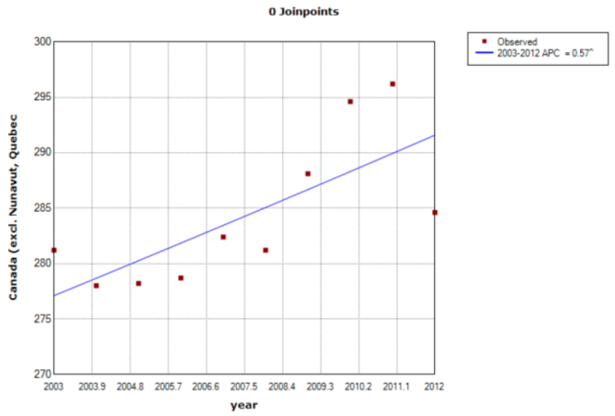

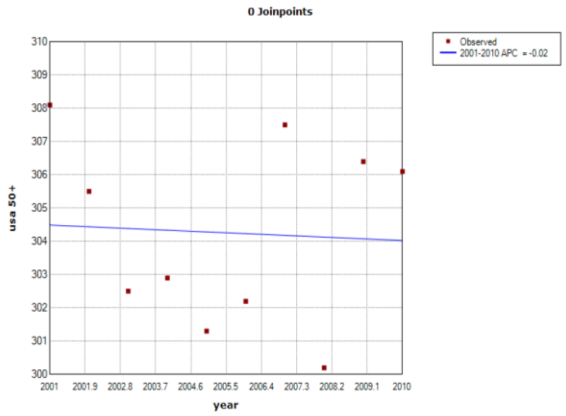

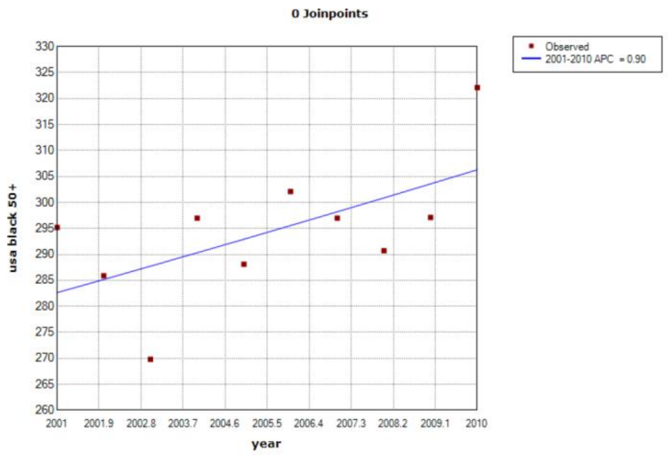
0 Joinpoints
Observed
2001-2010 APC = 0.90
usa black 50+
year

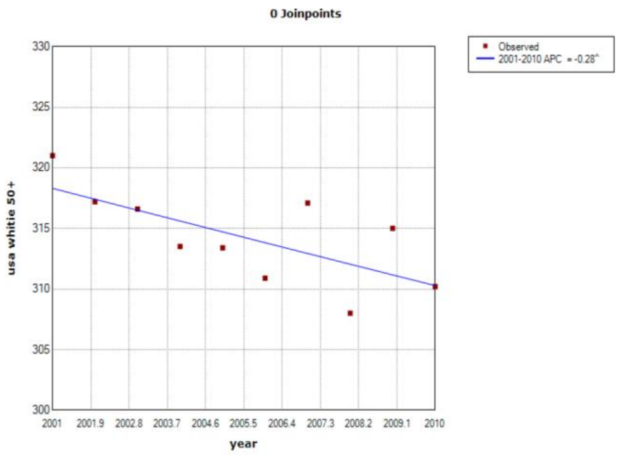
0 Joinpoints
Observed
2001-2010 APC = -0.28^
usa whitie 50+
year

Asia

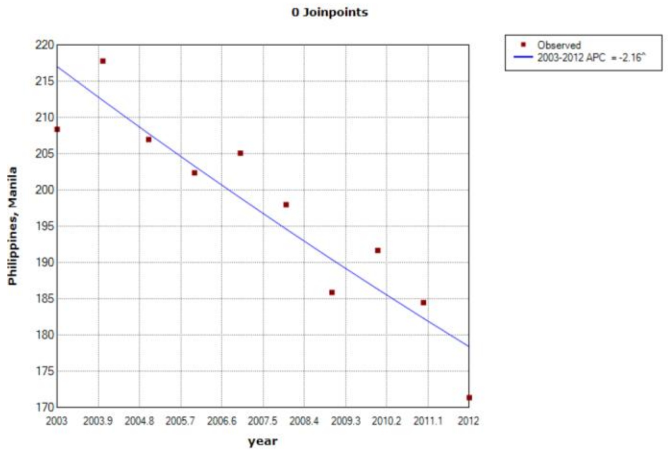
0 Joinpoints
Observed
2003-2012 APC = -2.16^
Philippines, Manila
year

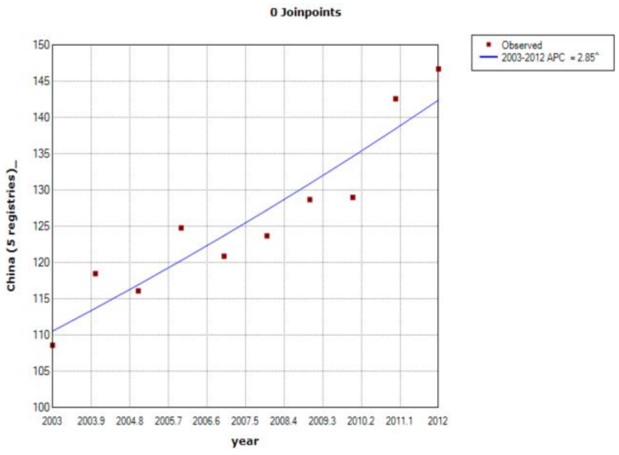
0 Joinpoints
Observed
2003-2012 APC = 2.85^
China (5 registries)_
year

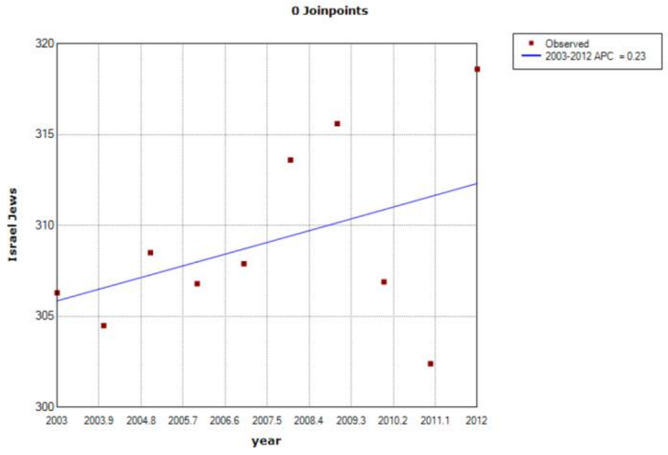
0 Joinpoints
Observed
2003-2012 APC = 0.23
Israel Jews
year

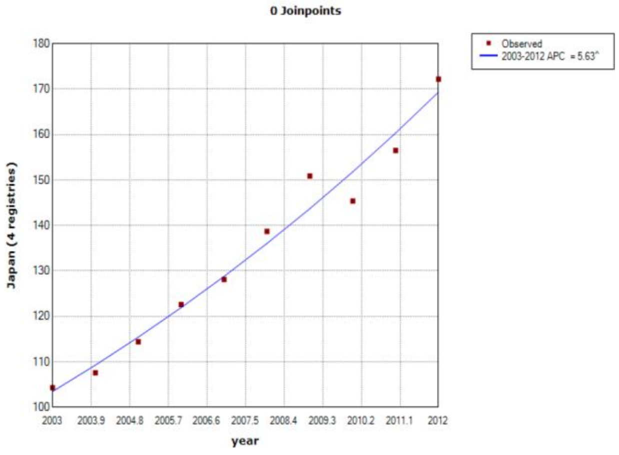
0 Joinpoints
Observed
2003-2012 APC = 5.63^
Japan (4 registries)
year

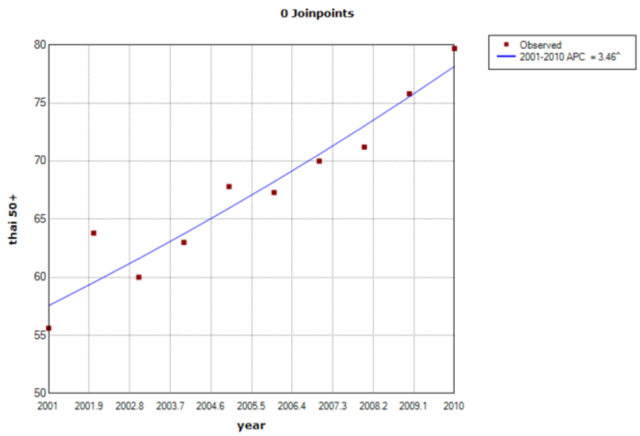

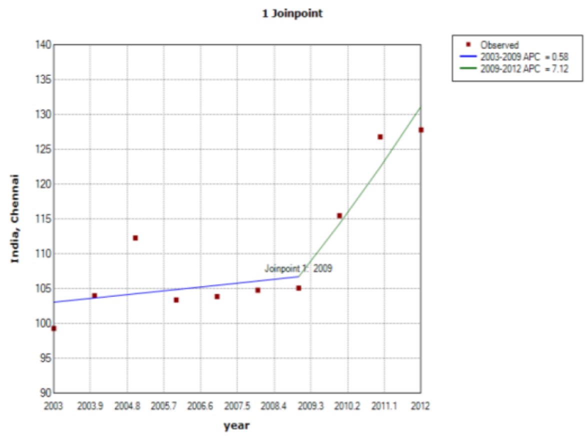

Oceania

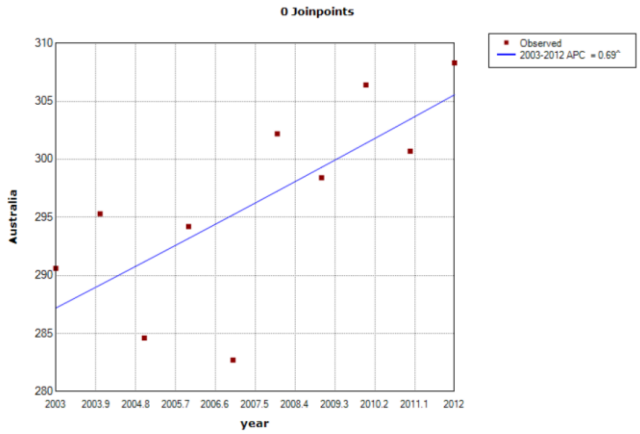

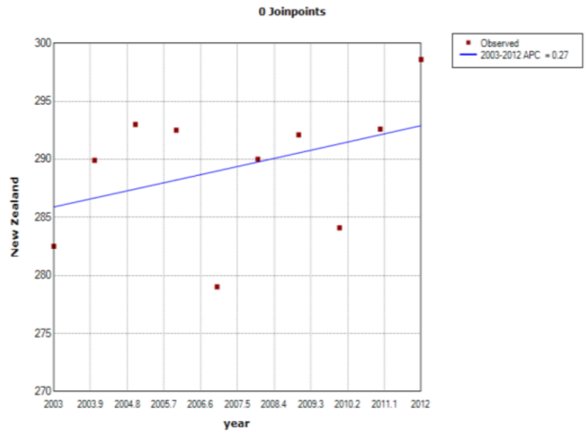

Northern Europe

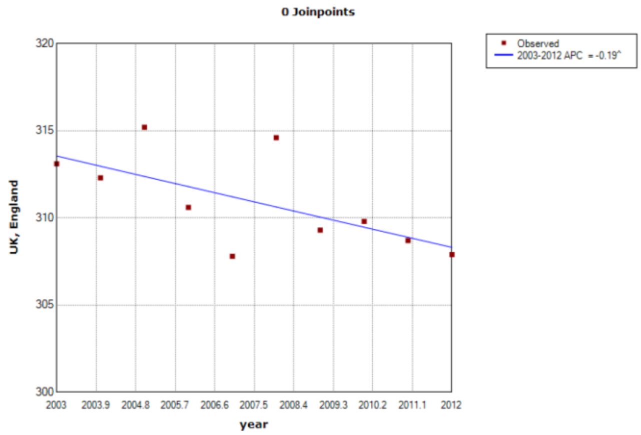

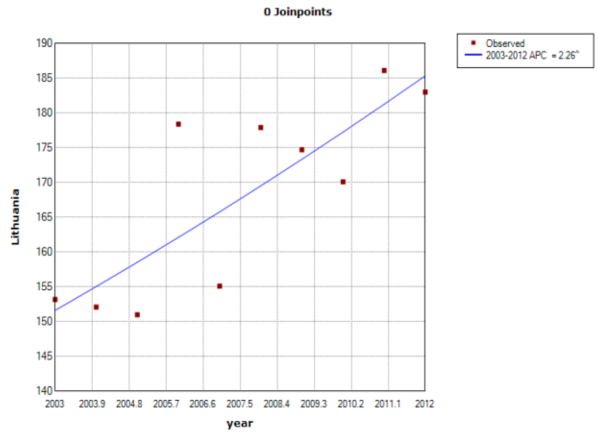

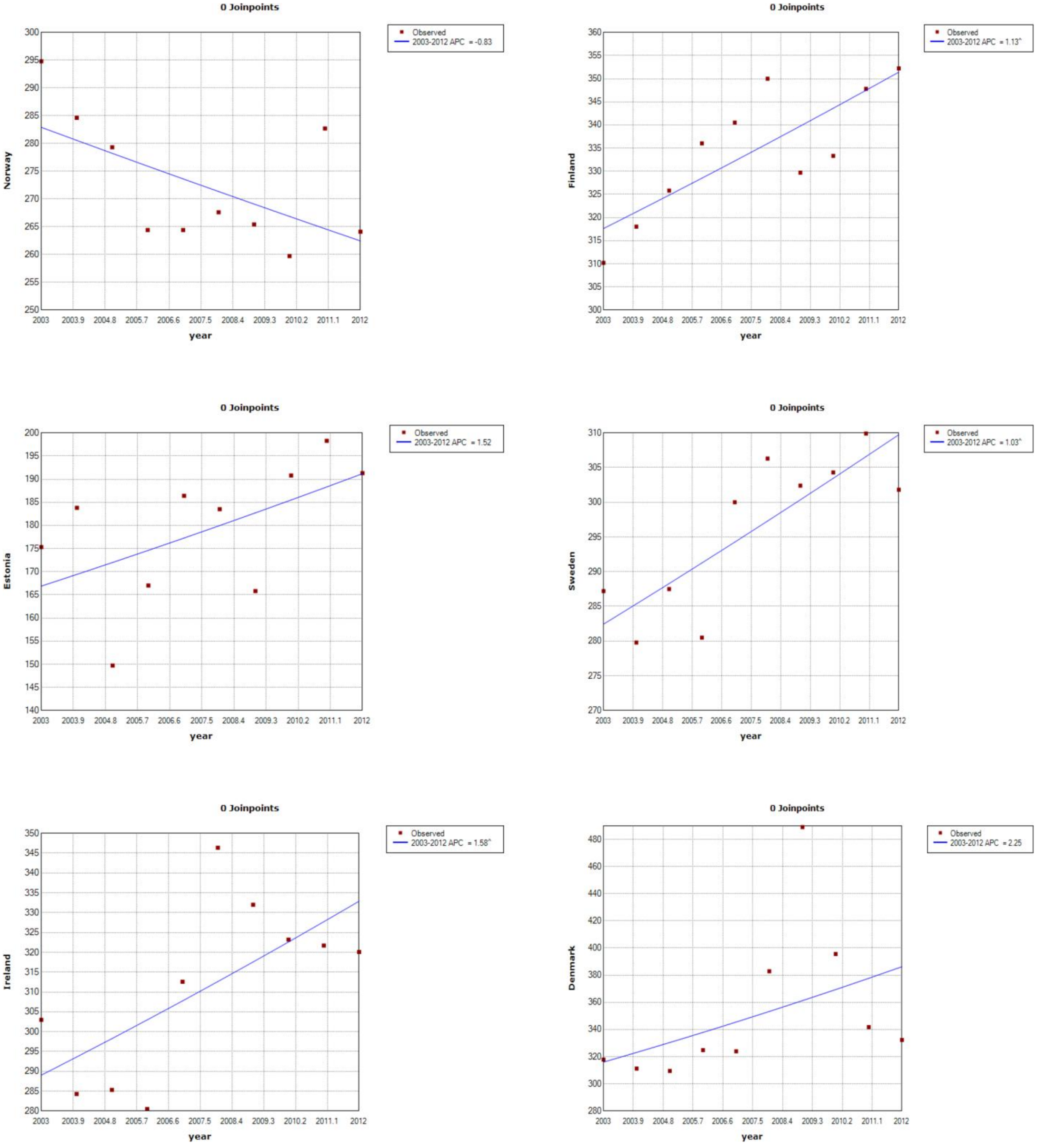
0 Joinpoints
Observed
2003-2012 APC = -0.83
Norway
year
0 Joinpoints
Observed
2003-2012 APC = 1.13^
Finland
year
0 Joinpoints
Observed
2003-2012 APC = 1.52
Estonia
year
0 Joinpoints
Observed
2003-2012 APC = 1.03^
Sweden
year
0 Joinpoints
Observed
2003-2012 APC = 1.58^
Ireland
year
0 Joinpoints
Observed
2003-2012 APC = 2.25
Denmark
year

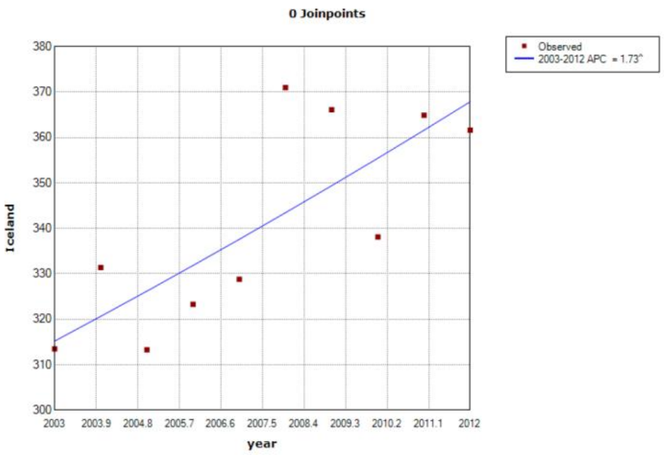

Western Europe

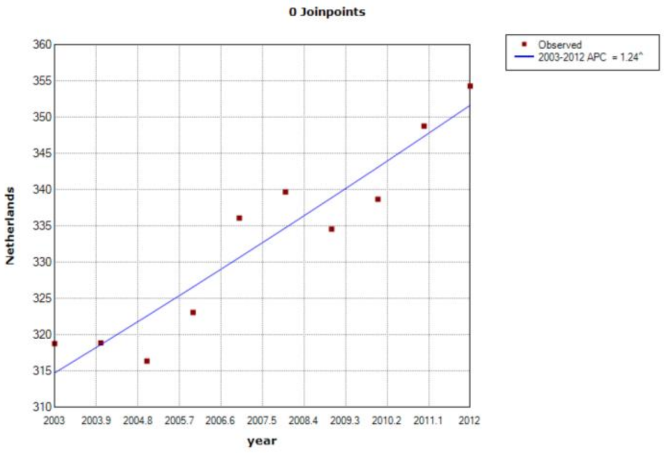

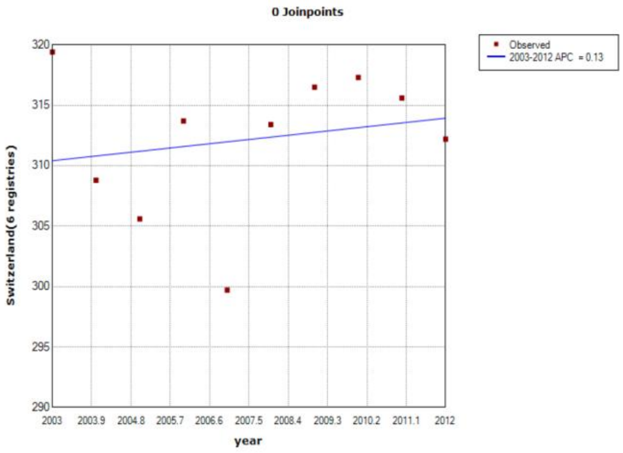

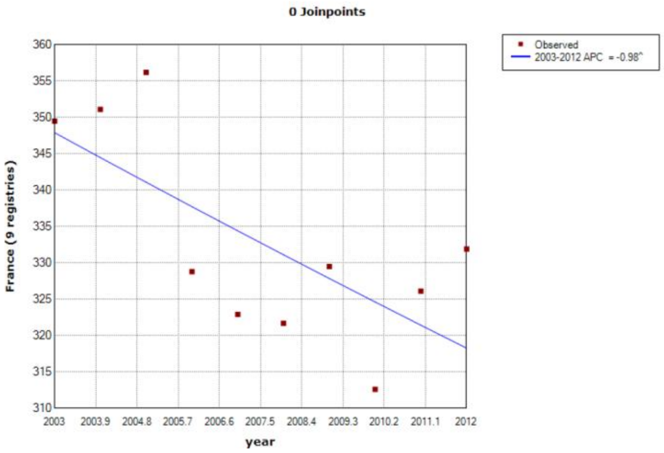

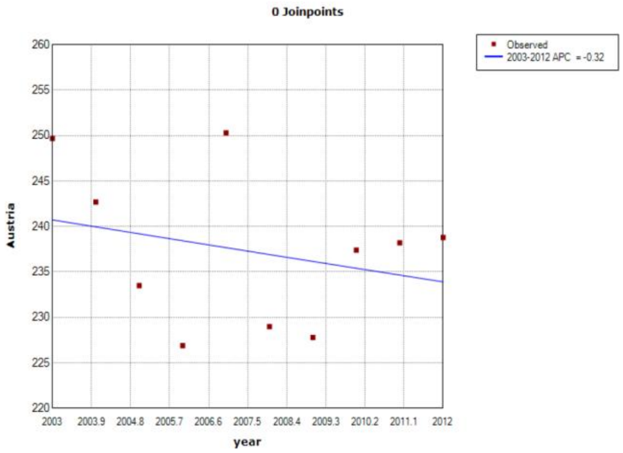

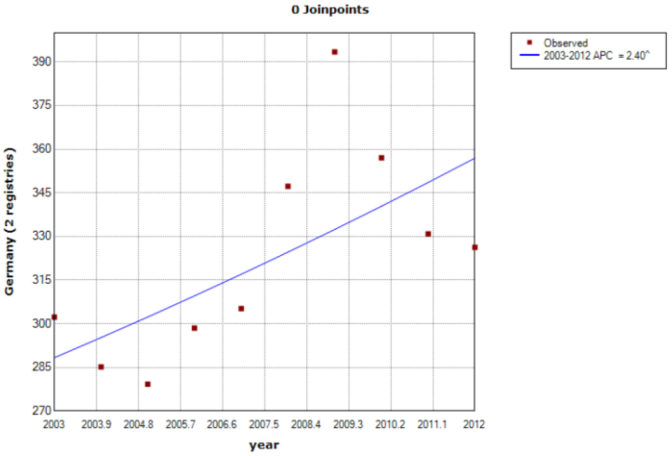

Southern Europe

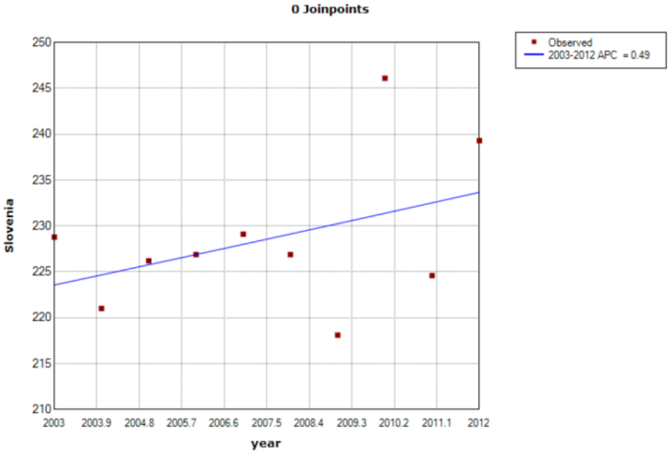

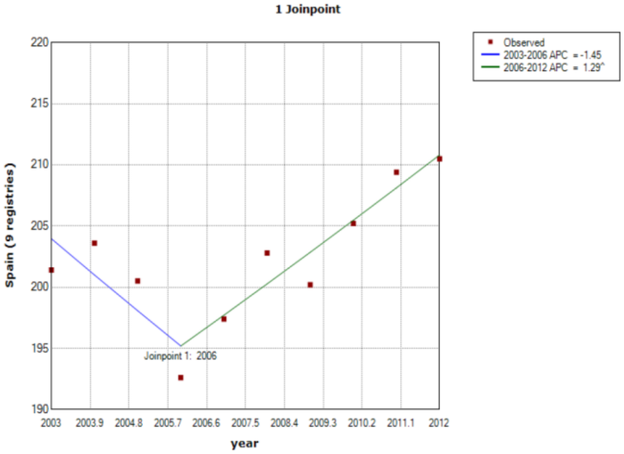

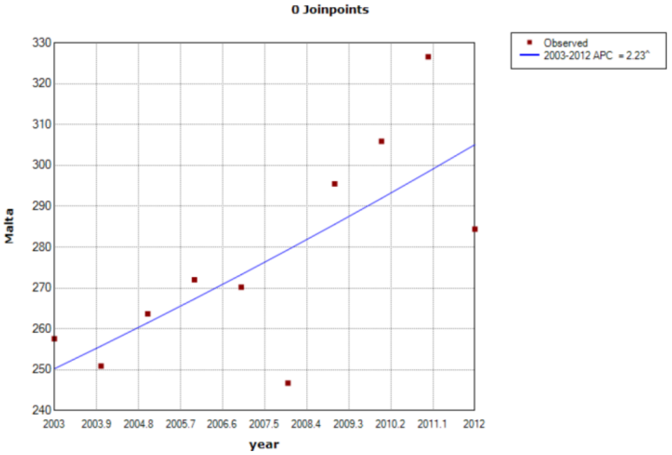

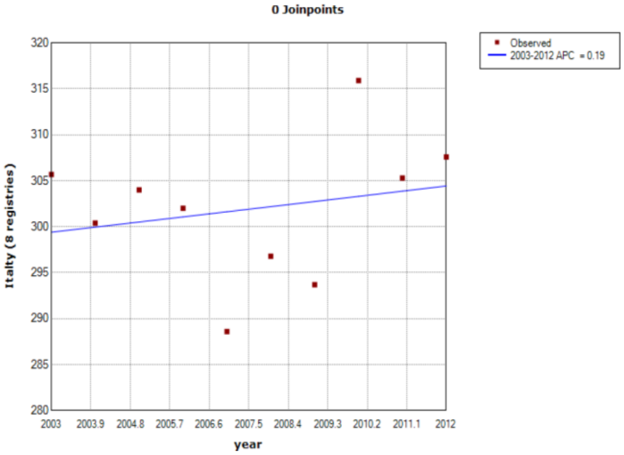

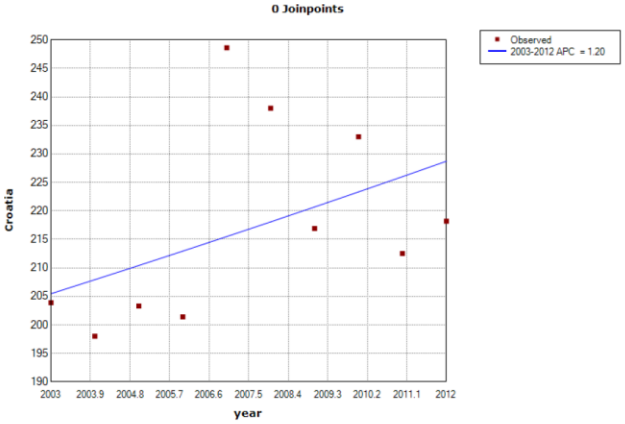

Eastern Europe

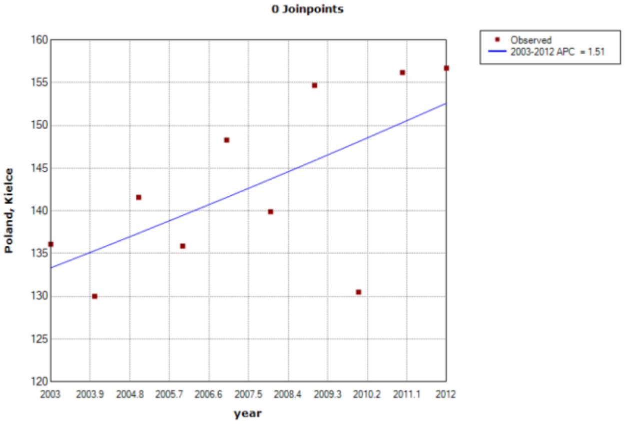

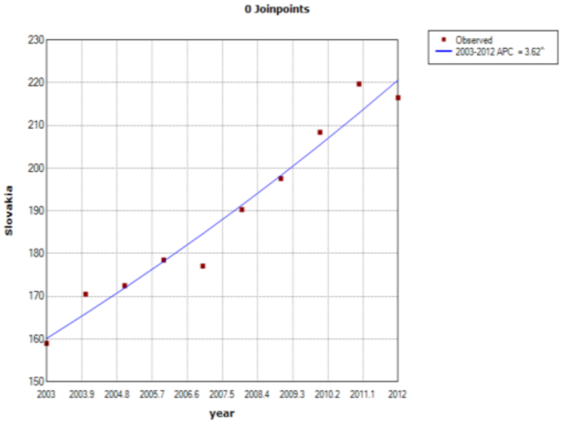

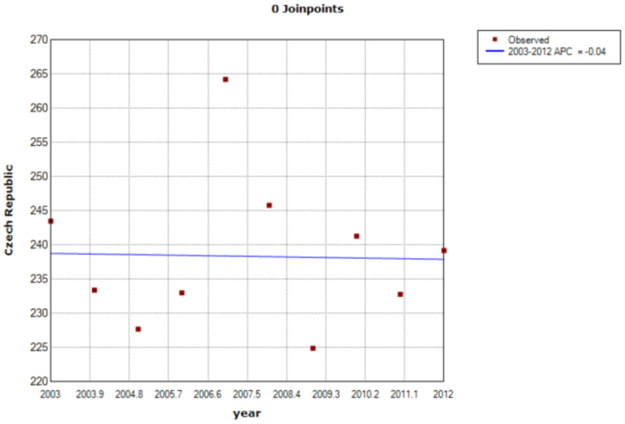

Latin America & the Caribbean

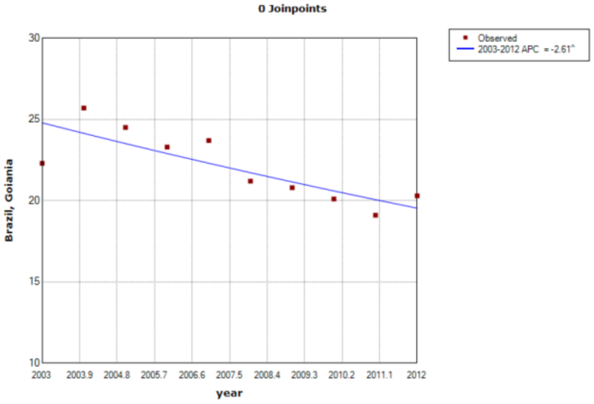

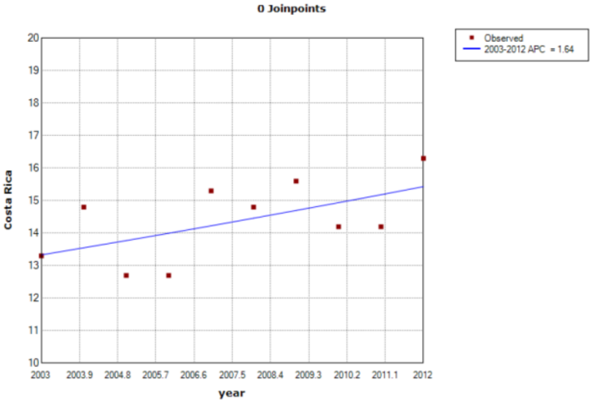

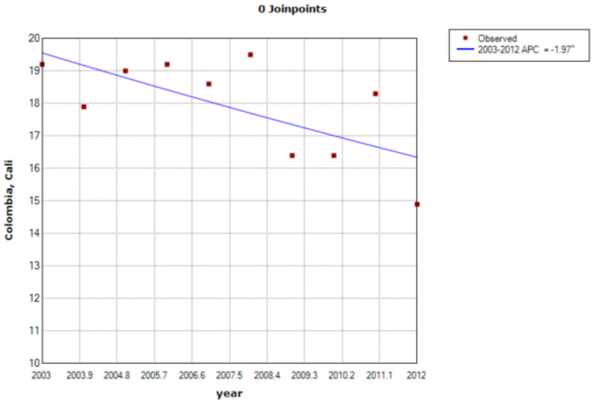

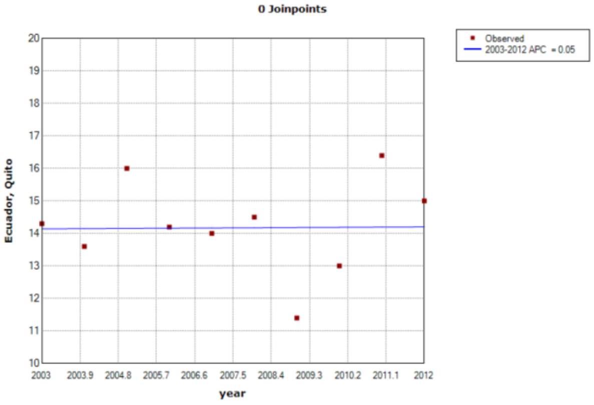

Northern America

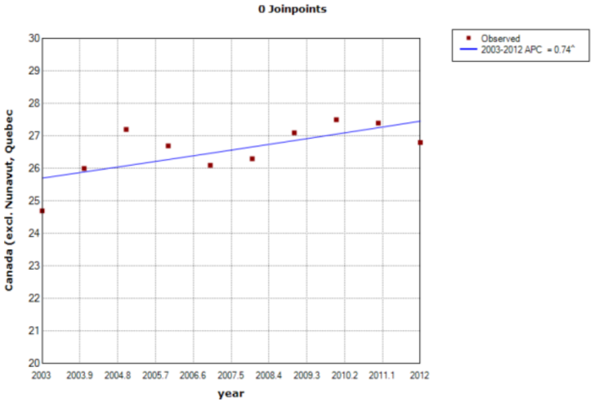

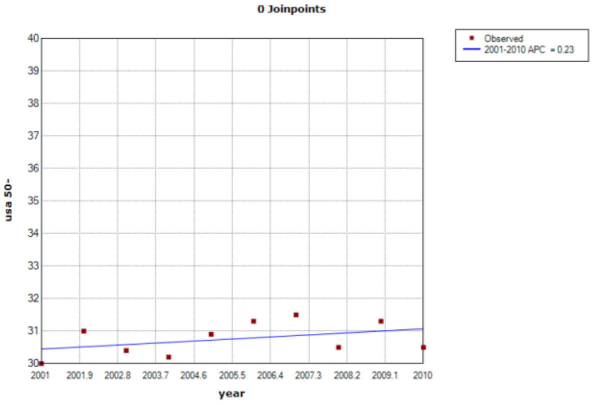

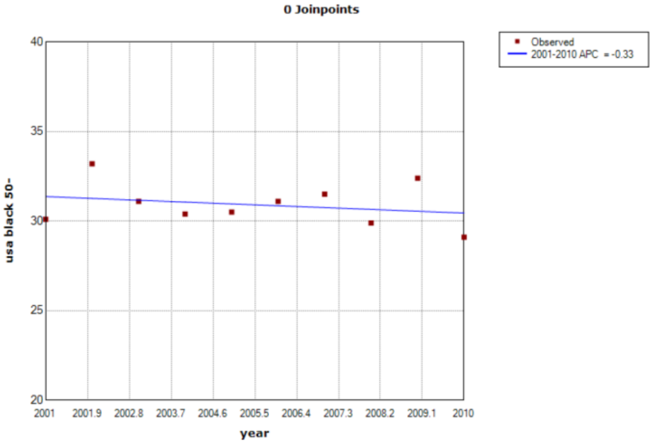
0 Joinpoints
Observed
2001-2010 APC = -0.33
usa black 50-
year

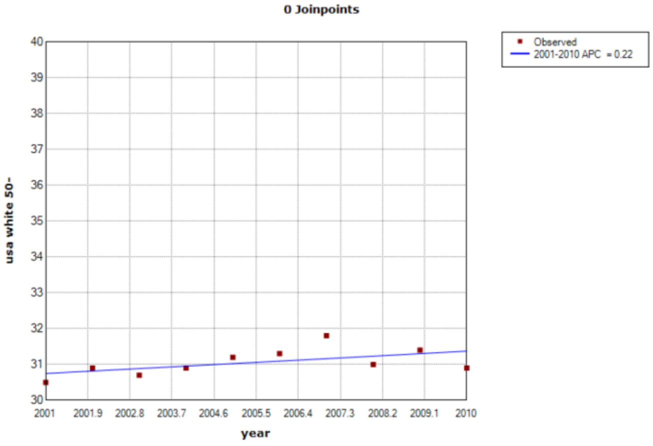
0 Joinpoints
Observed
2001-2010 APC = 0.22
usa white 50-
year

Asia

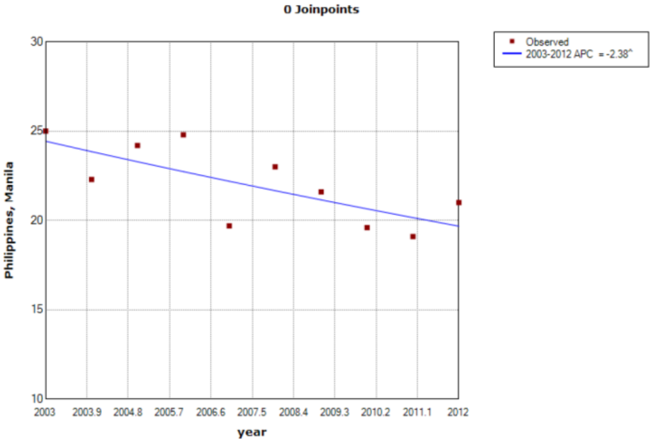
0 Joinpoints
Observed
2003-2012 APC = -2.38^
Philippines, Manila
year

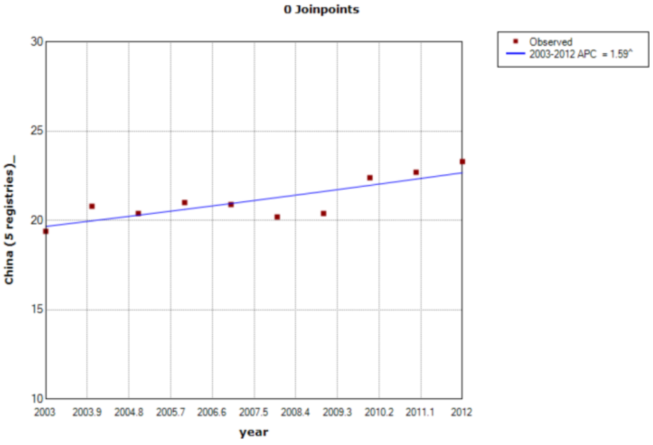
0 Joinpoints
Observed
2003-2012 APC = 1.59^
China (5 registries)_
year

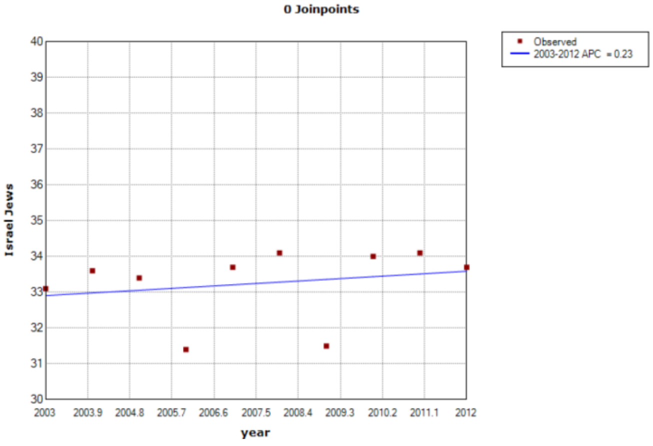
0 Joinpoints
Observed
2003-2012 APC = 0.23
Israel Jews
year

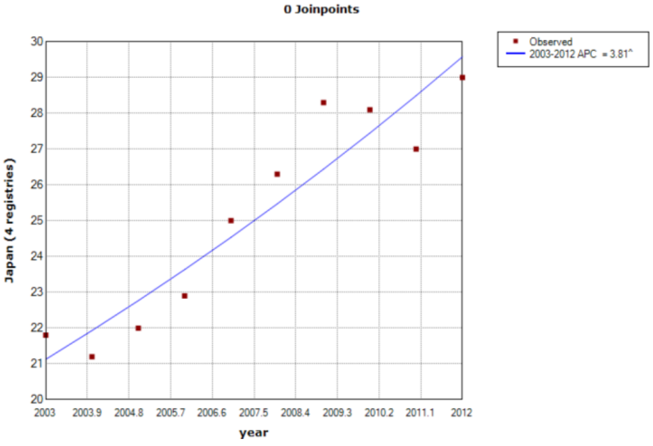
0 Joinpoints
Observed
2003-2012 APC = 3.81^
Japan (4 registries)
year

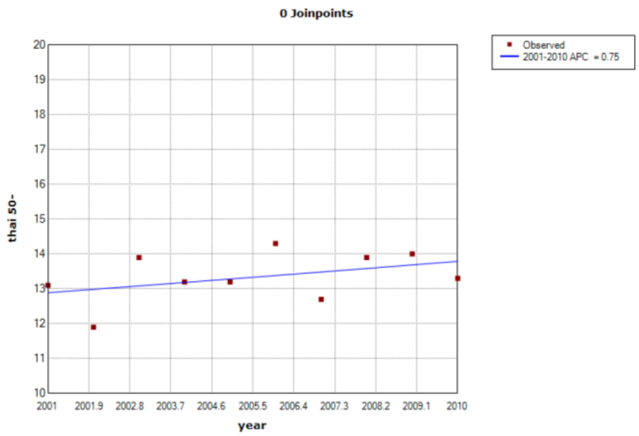

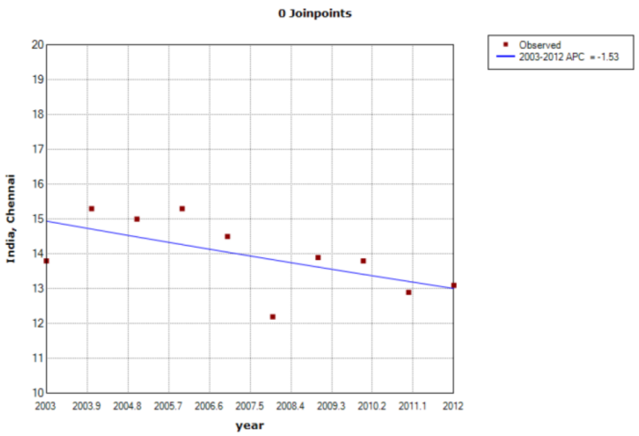

Oceania

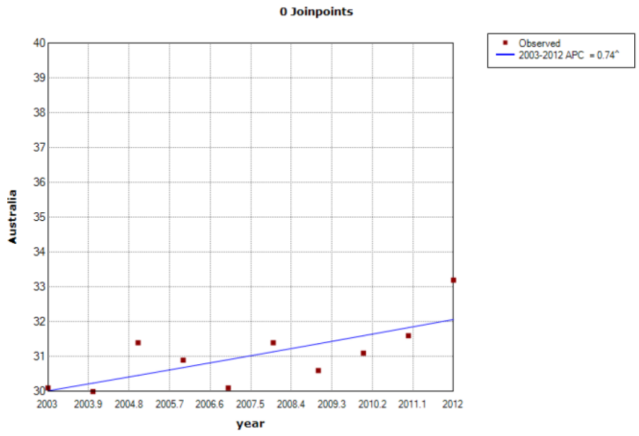

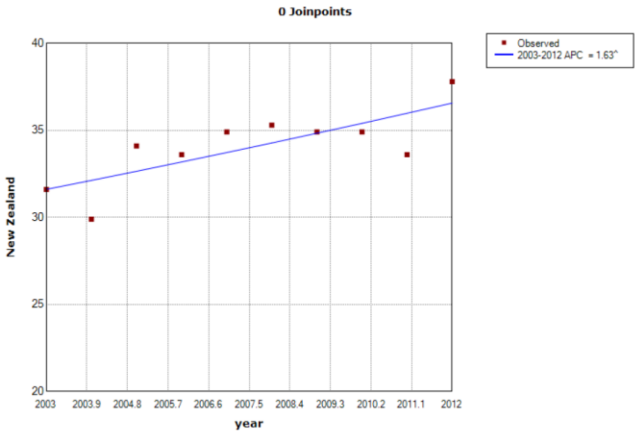

Northern Europe

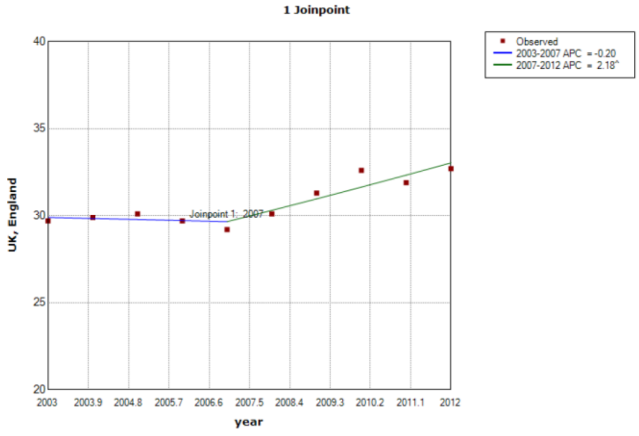

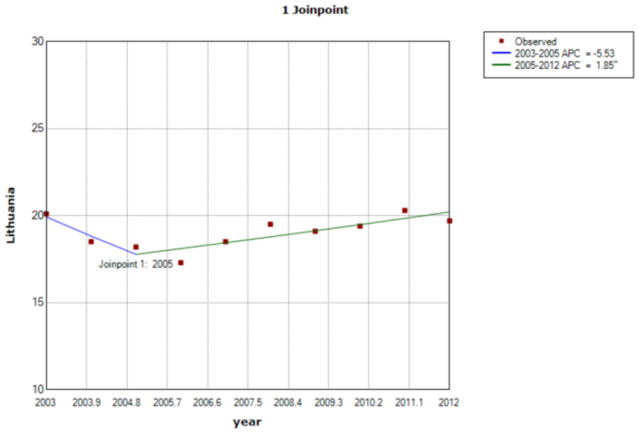

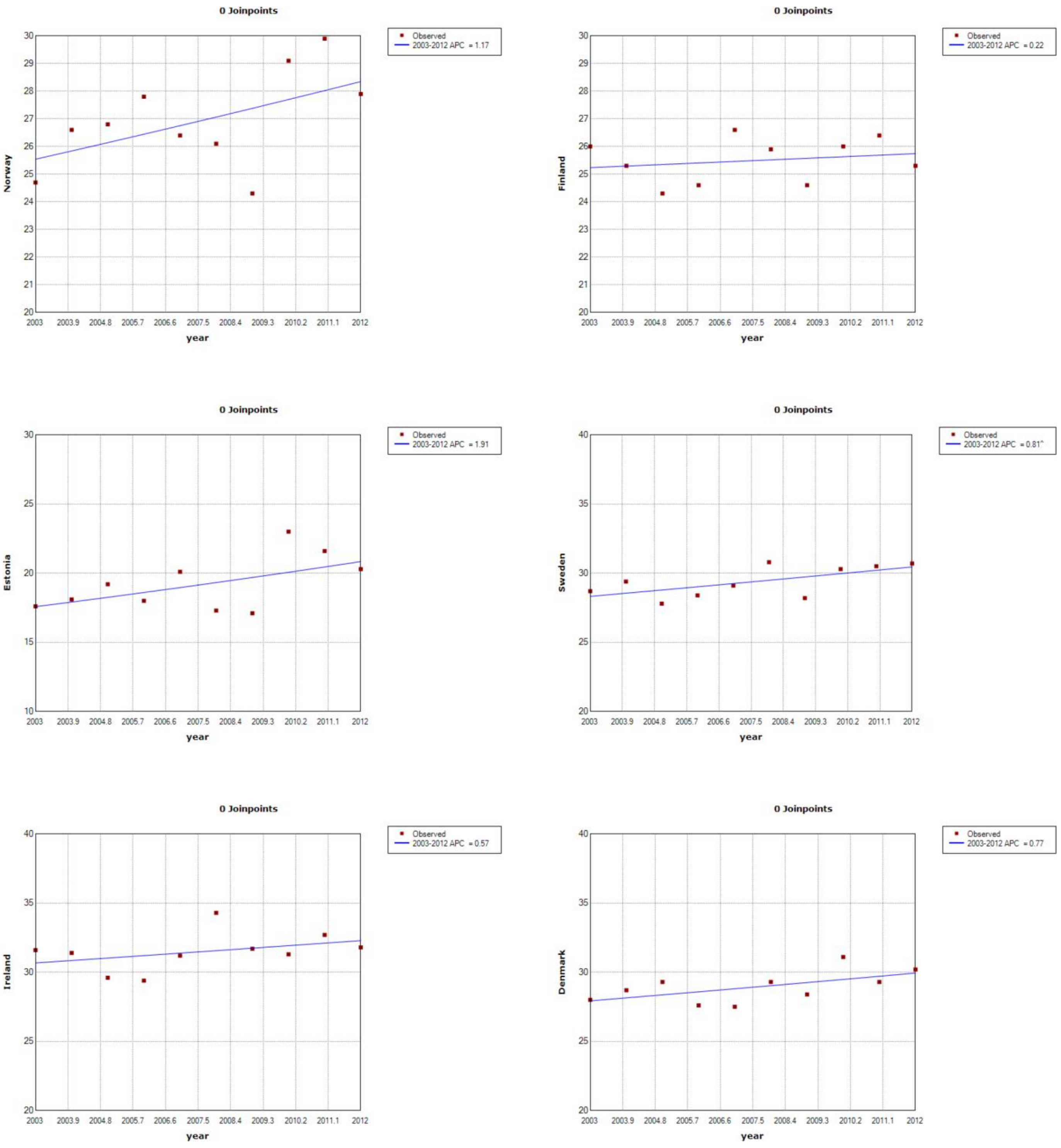
0 Joinpoints
Observed
2003-2012 APC = 1.17
Norway
year
0 Joinpoints
Observed
2003-2012 APC = 0.22
Finland
year
0 Joinpoints
Observed
2003-2012 APC = 1.91
Estonia
year
0 Joinpoints
Observed
2003-2012 APC = 0.81^
Sweden
year
0 Joinpoints
Observed
2003-2012 APC = 0.57
Ireland
year
0 Joinpoints
Observed
2003-2012 APC = 0.77
Denmark
year

V

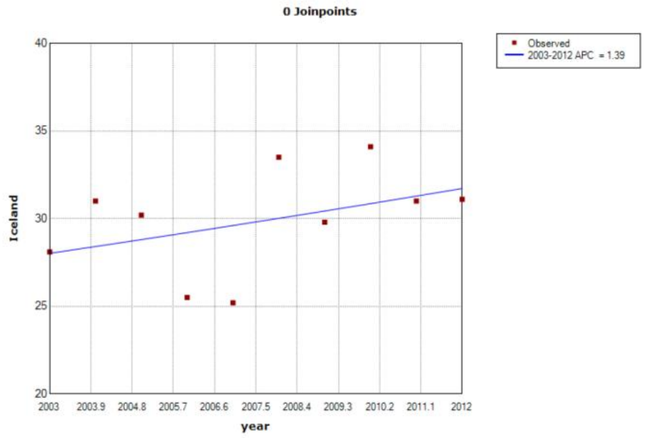

Western Europe

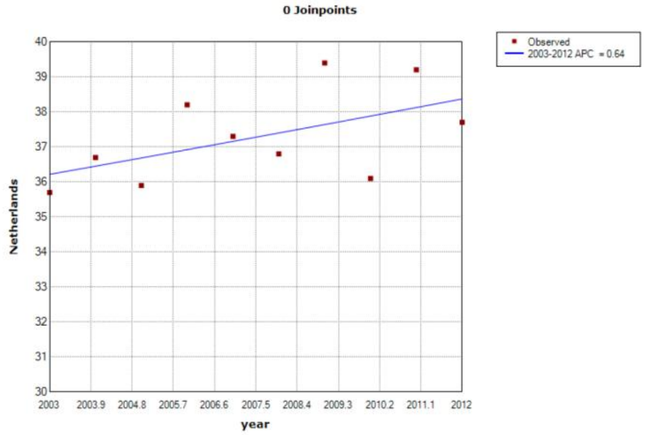

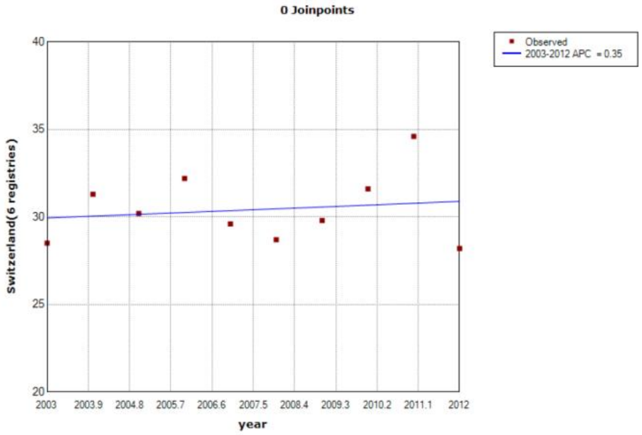

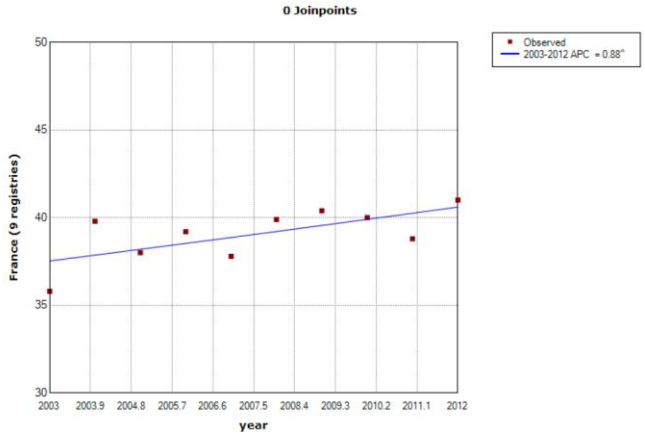

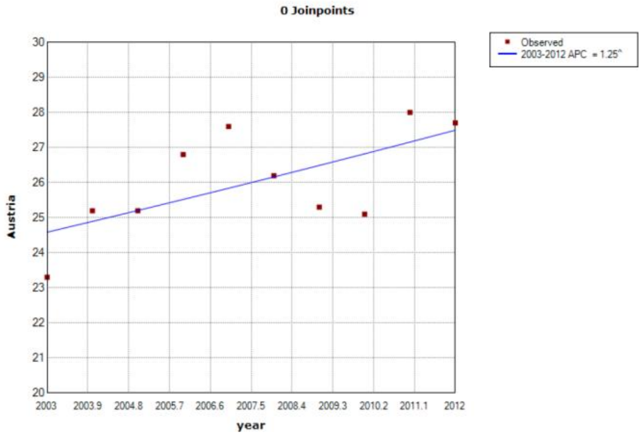

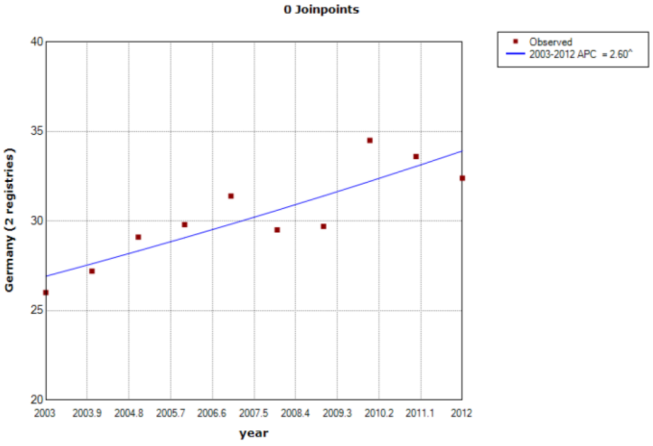

Southern Europe

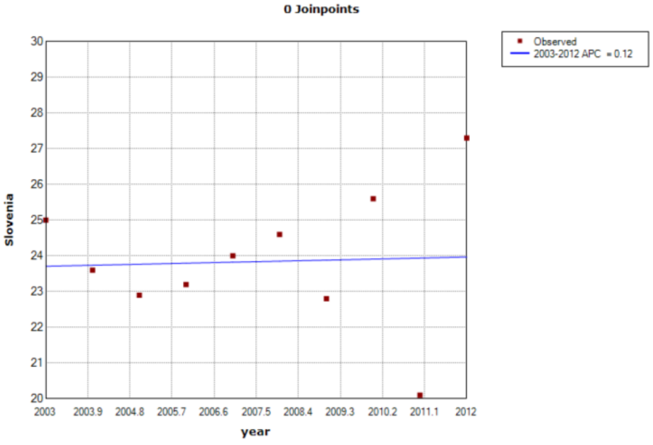

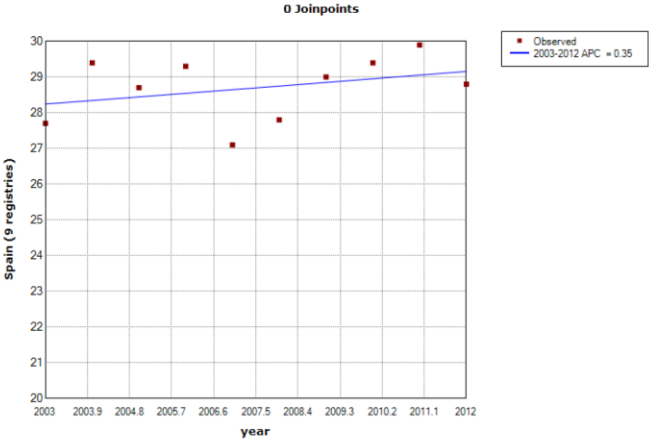

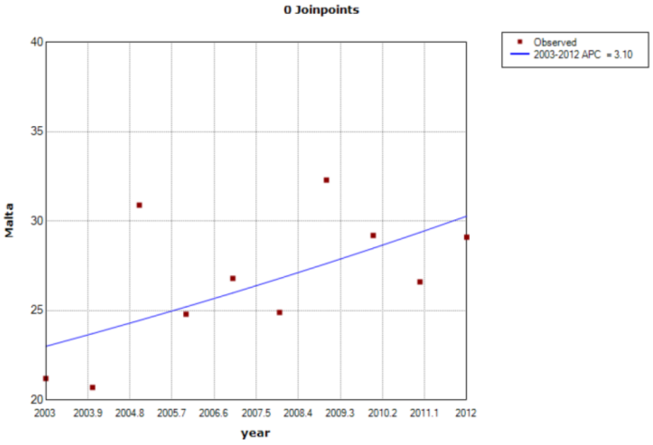

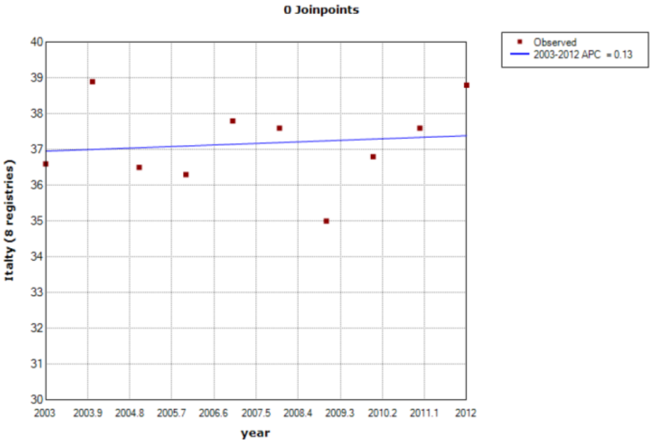

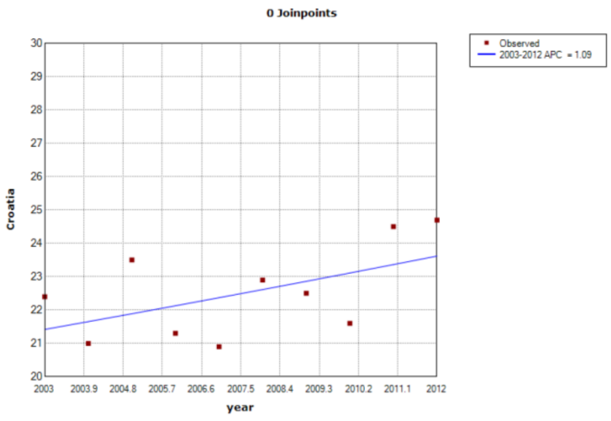

Eastern Europe

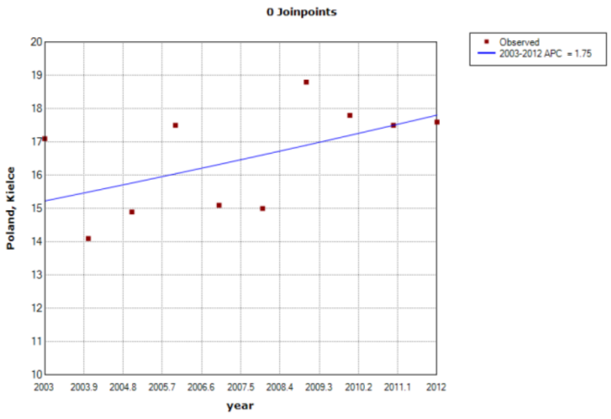

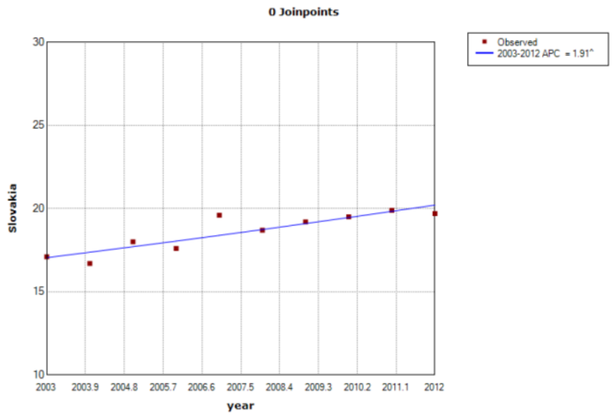

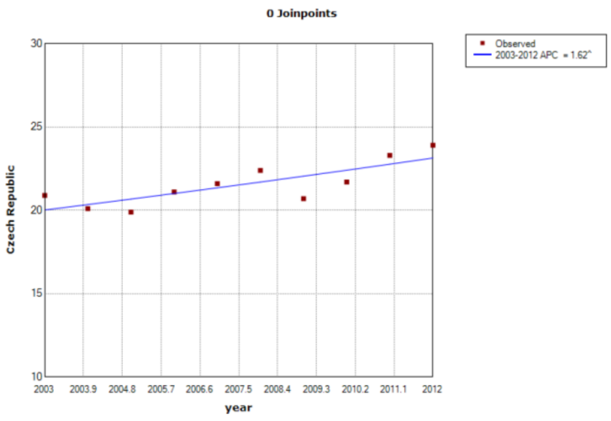

Latin America & the Caribbean

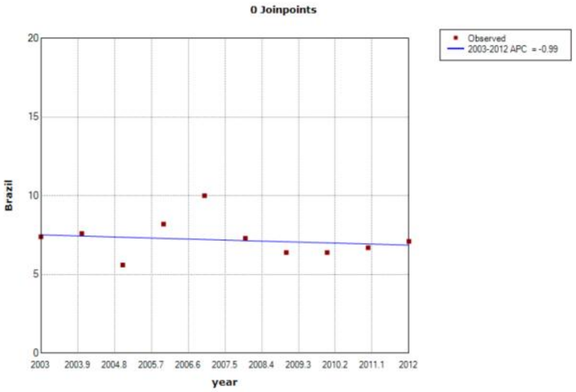

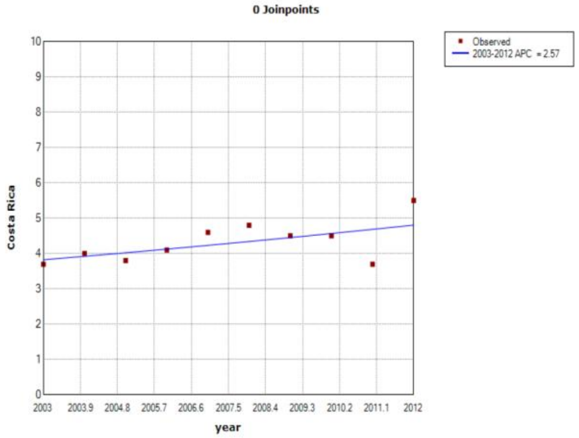

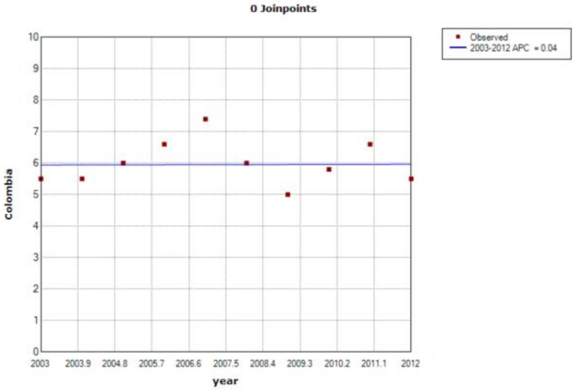

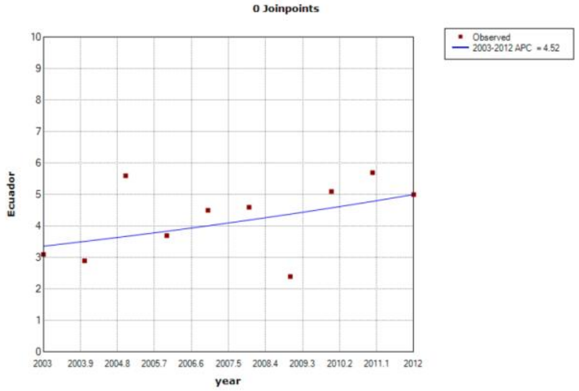

Northern America

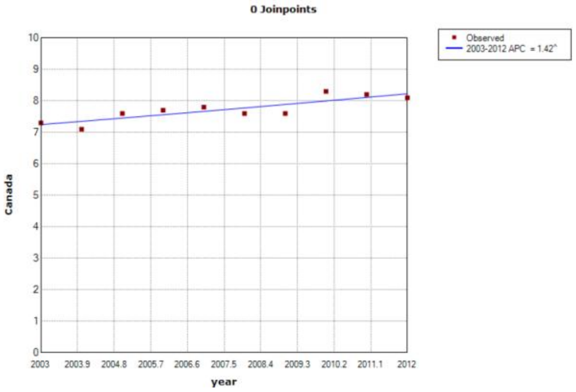

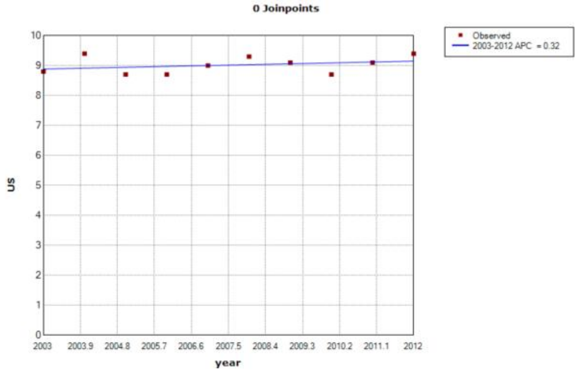

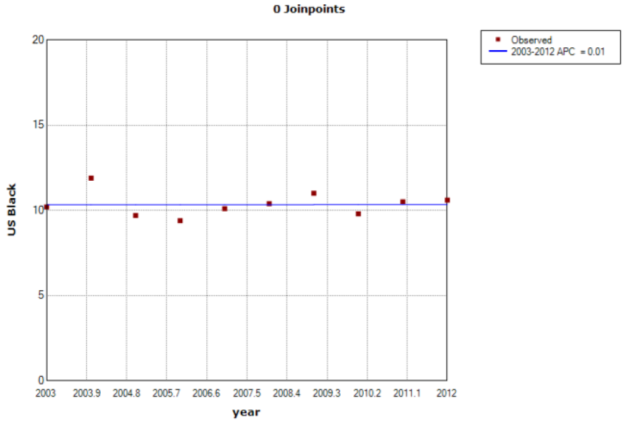
0 Joinpoints
Observed
2003-2012 APC = 0.01
US Black
year
2003
2003.9
2004.8
2005.7
2006.6
2007.5
2008.4
2009.3
2010.2
2011.1
2012

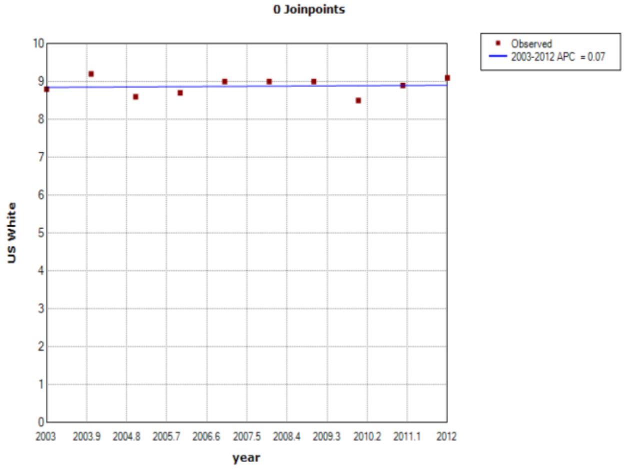
0 Joinpoints
Observed
2003-2012 APC = 0.07
US White
year

Asia

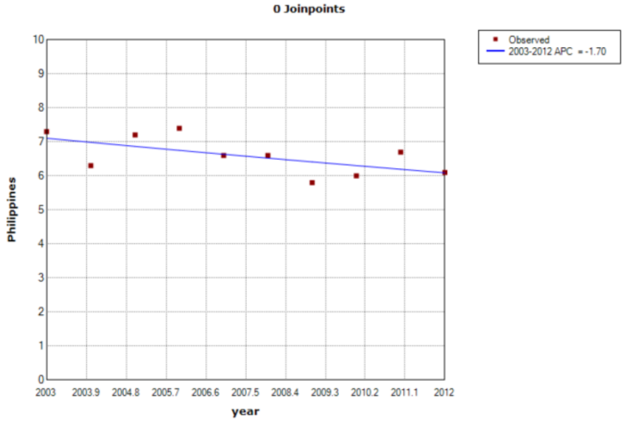
0 Joinpoints
Observed
2003-2012 APC = -1.70
Philippines
year

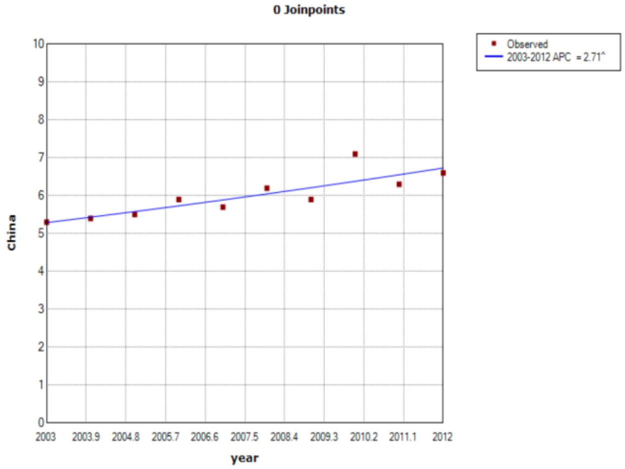
0 Joinpoints
Observed
2003-2012 APC = 2.71^
China
year

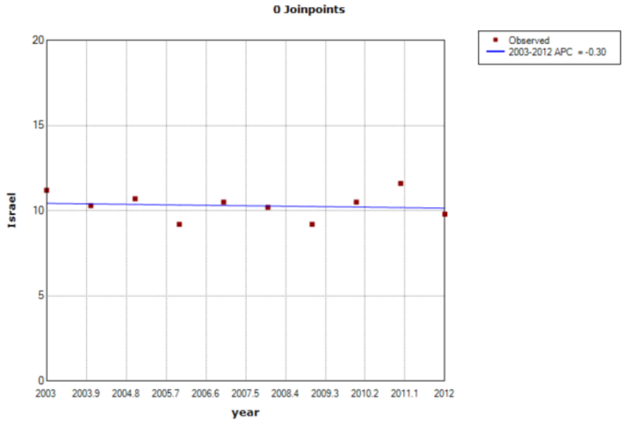
0 Joinpoints
Observed
2003-2012 APC = -0.30
Israel
year

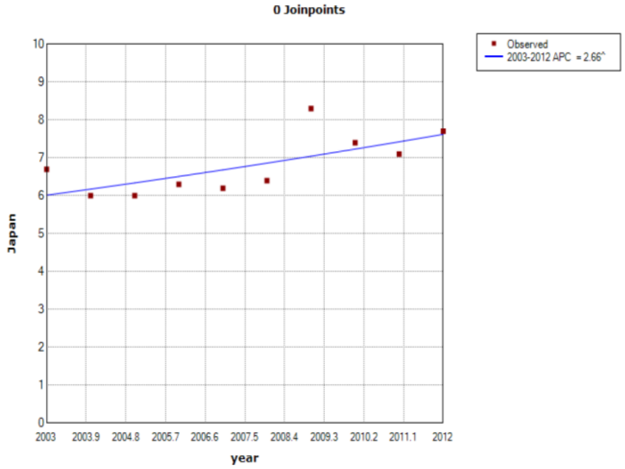
0 Joinpoints
Observed
2003-2012 APC = 2.66^
Japan
year

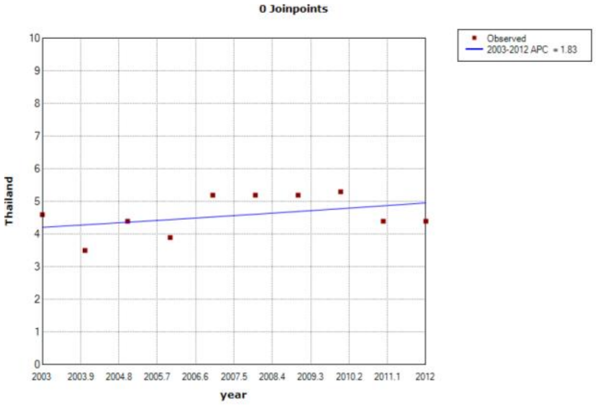

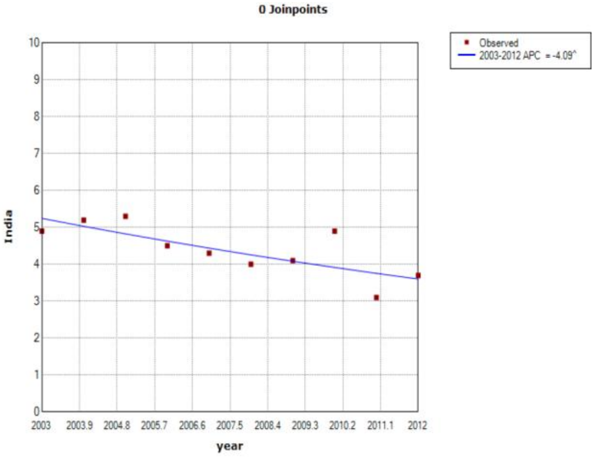

Oceania

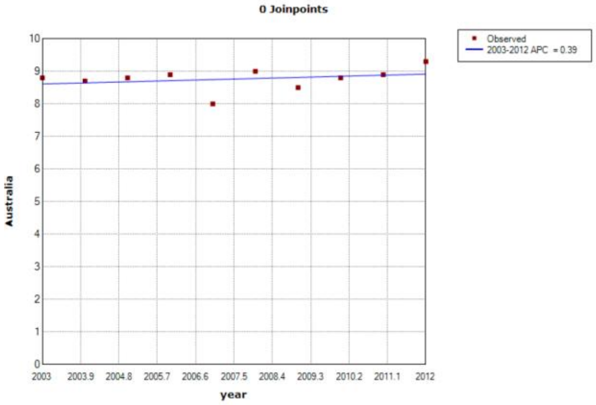

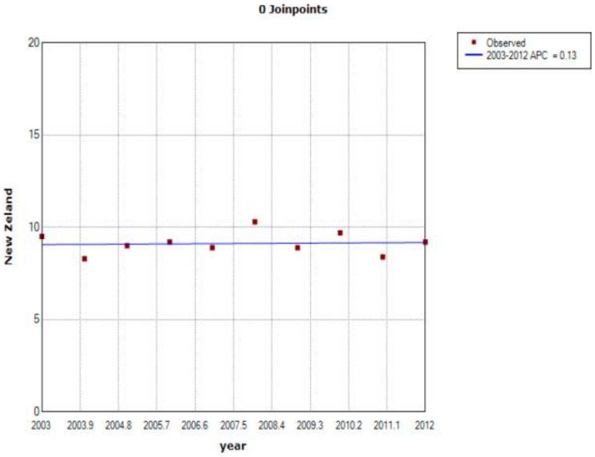

Northern Europe

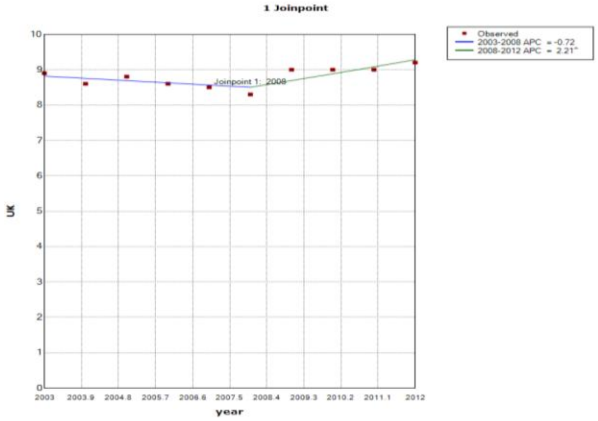

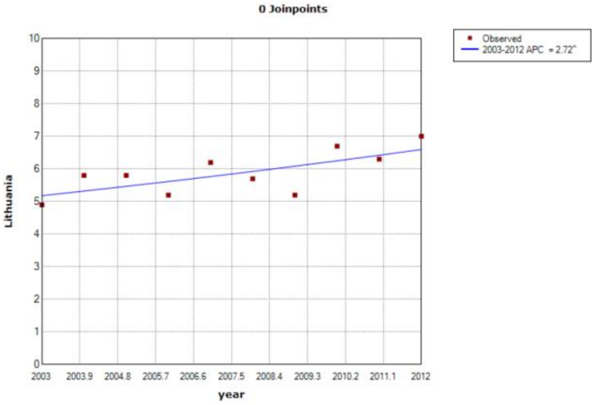

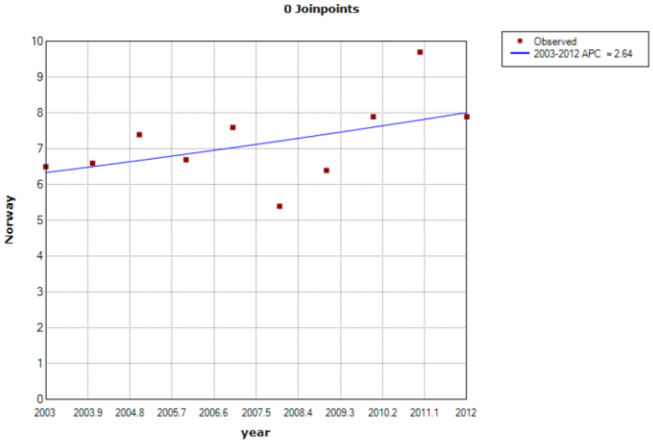
0 Joinpoints
Observed
2003-2012 APC = 2.64
Norway
year

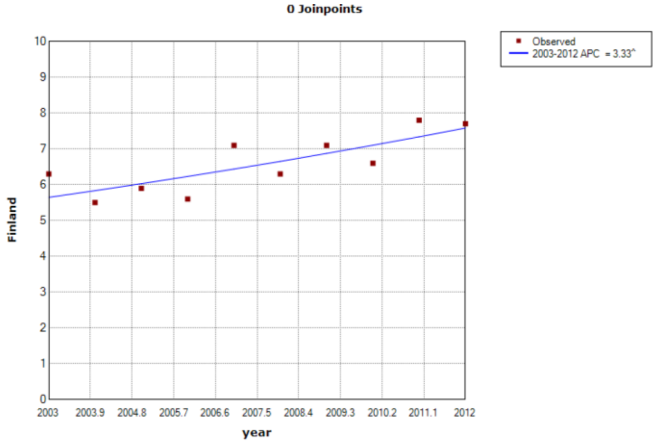
0 Joinpoints
Observed
2003-2012 APC = 3.33^
Finland
year

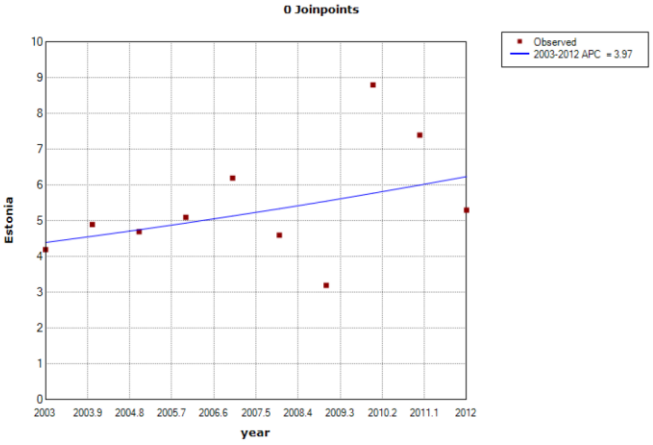
0 Joinpoints
Observed
2003-2012 APC = 3.97
Estonia
year

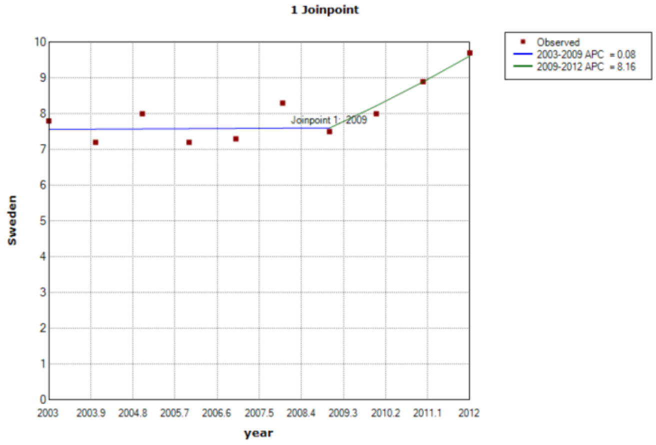
1 Joinpoint
Observed
2003-2009 APC = 0.08
2009-2012 APC = 8.16
Joinpoint 1 - 2009
Sweden
year

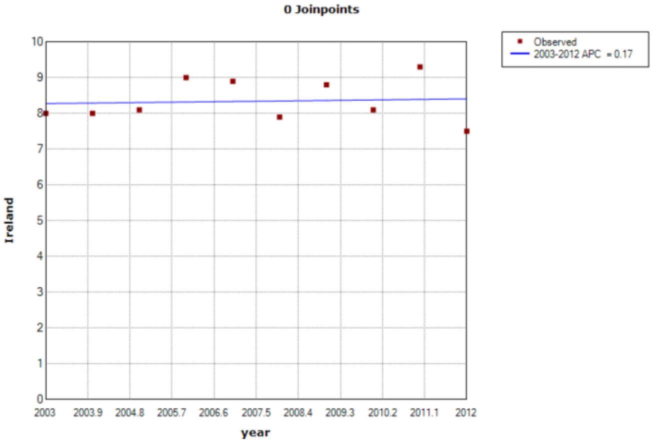
0 Joinpoints
Observed
2003-2012 APC = 0.17
Ireland
year

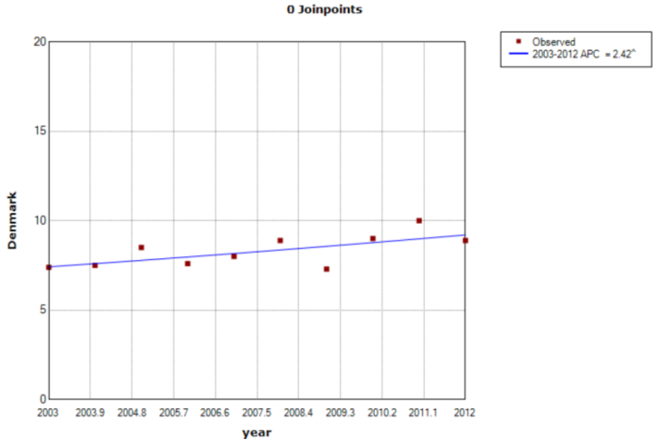
0 Joinpoints
Observed
2003-2012 APC = 2.42^
Denmark
year

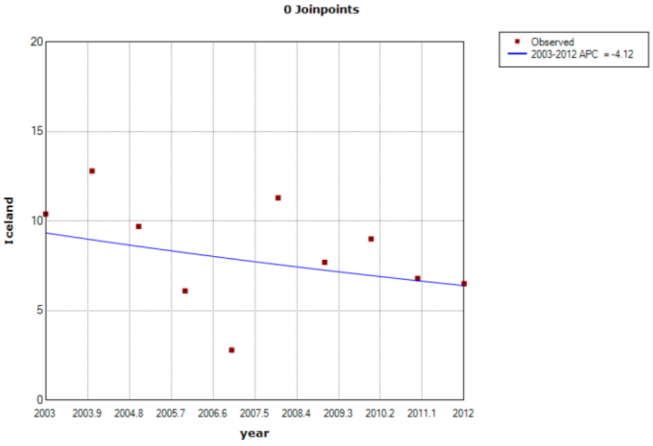

Western Europe

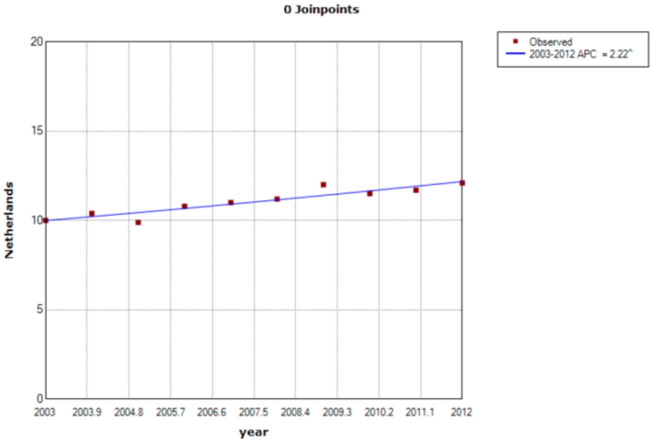

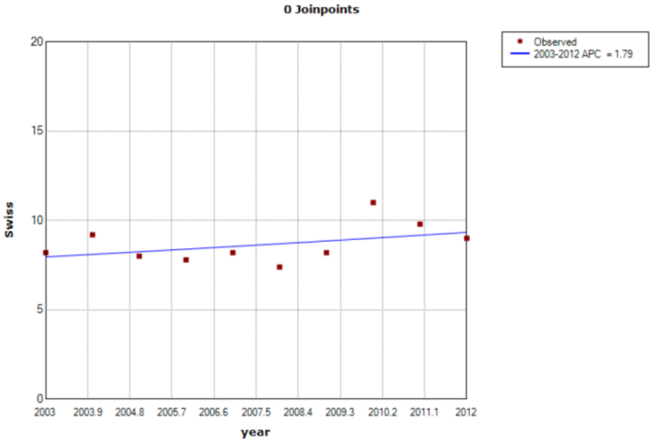

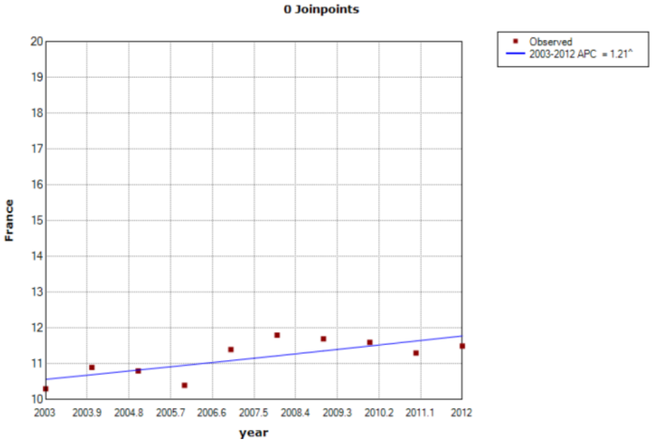

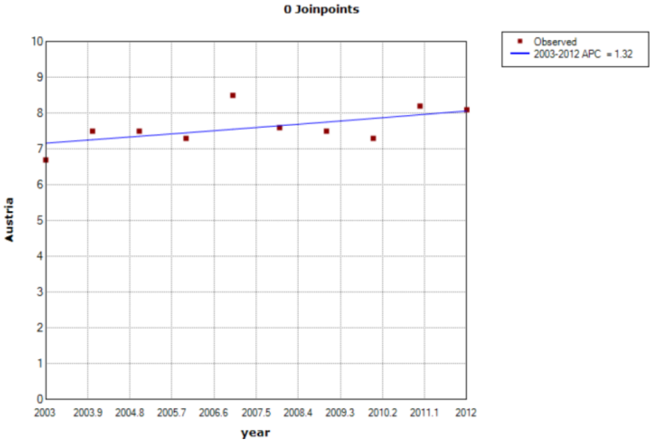

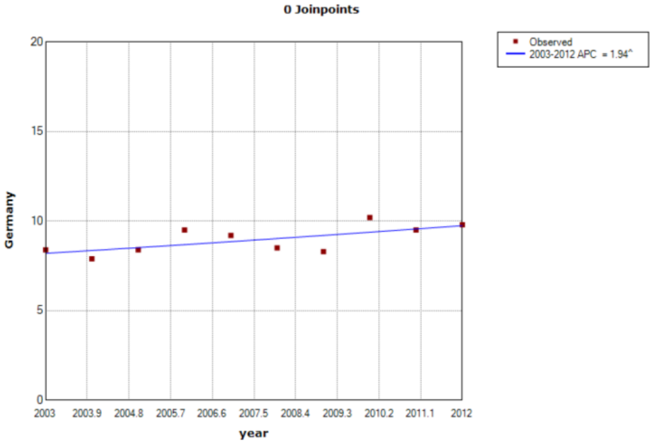

Southern Europe

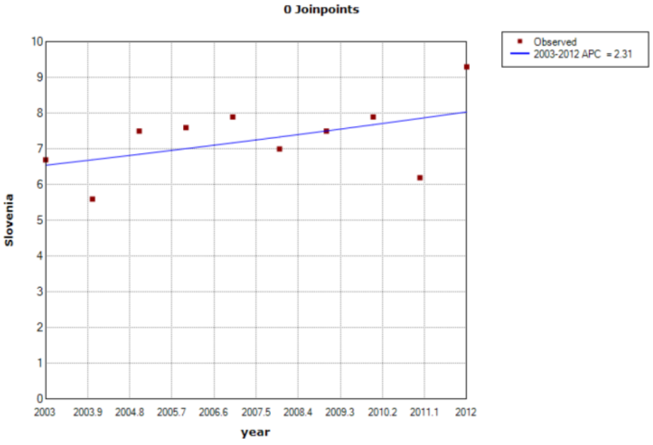

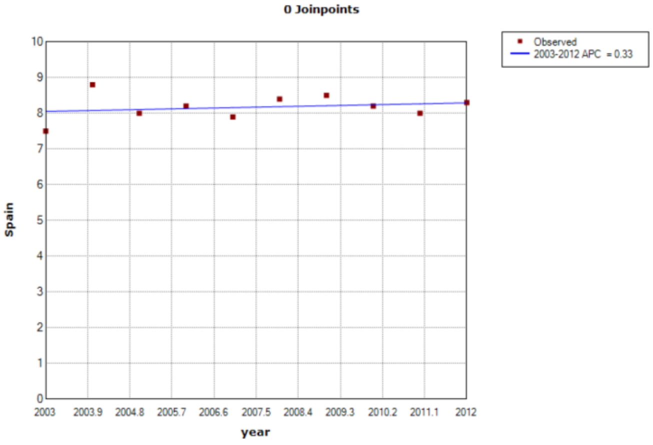

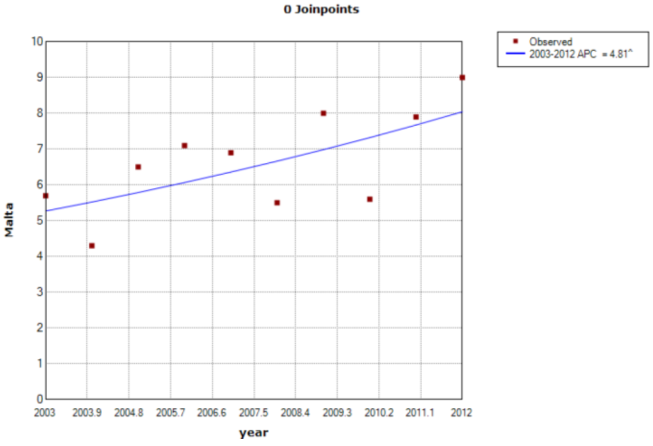

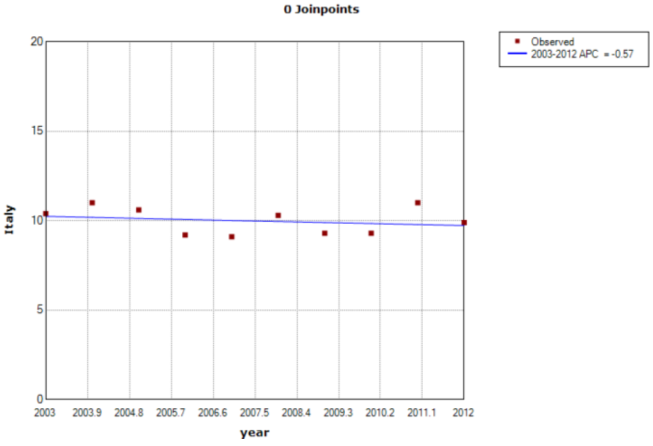

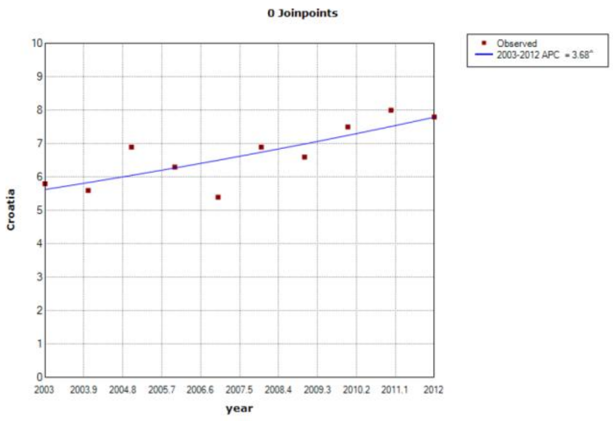

Eastern Europe

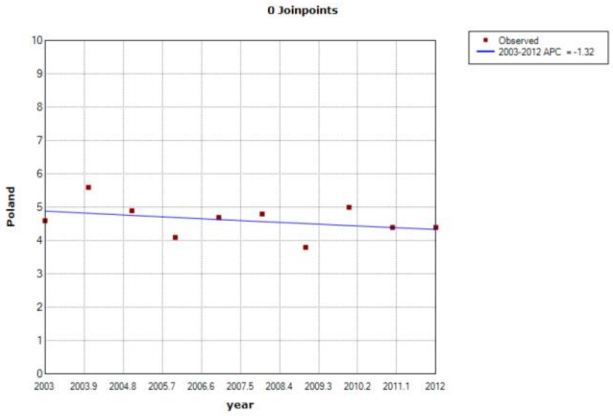

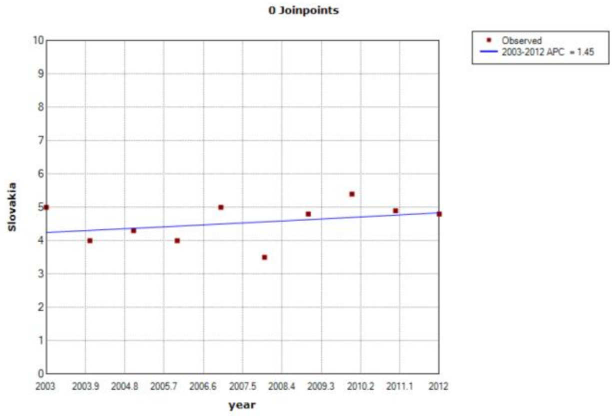

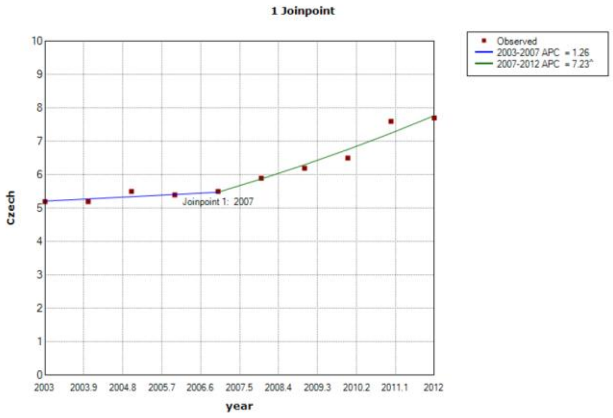

Latin America & the Caribbean

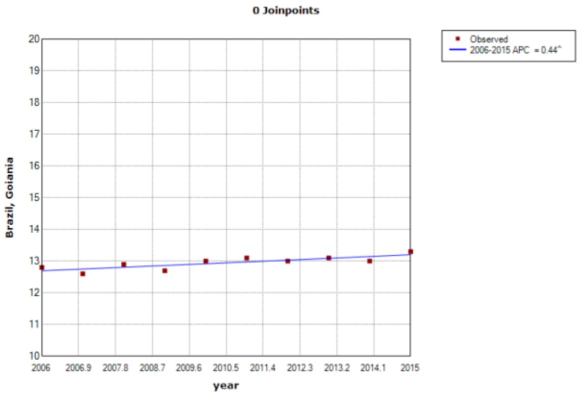

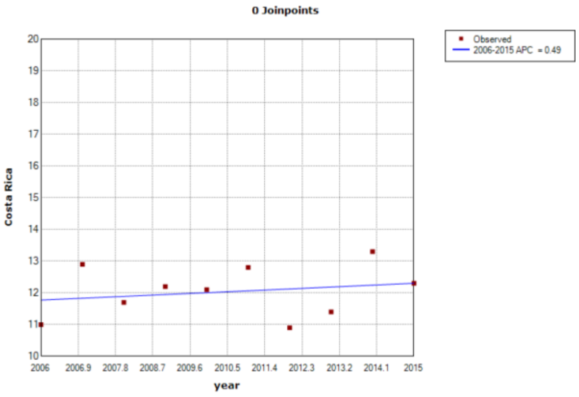

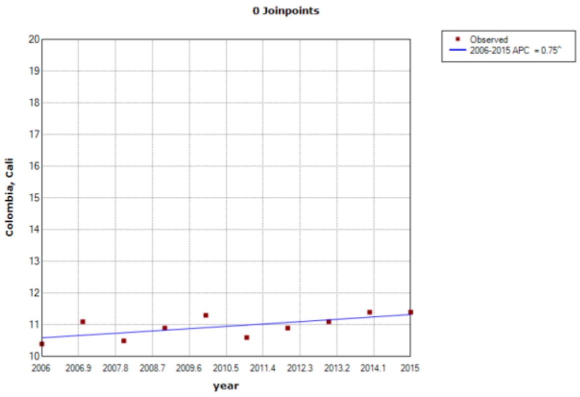

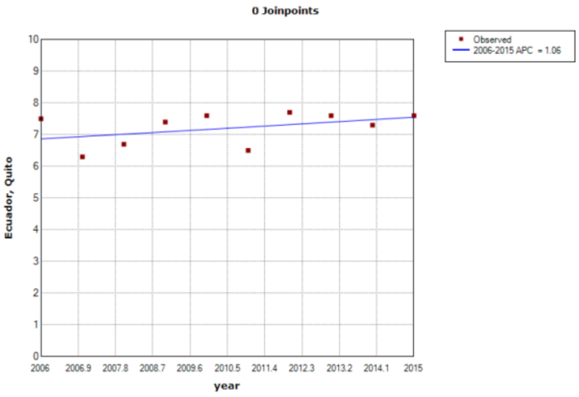

Northern America

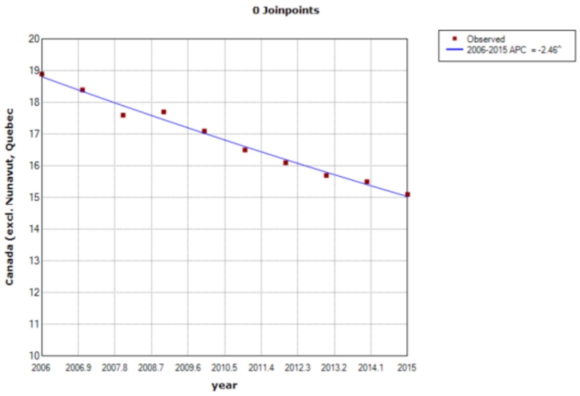

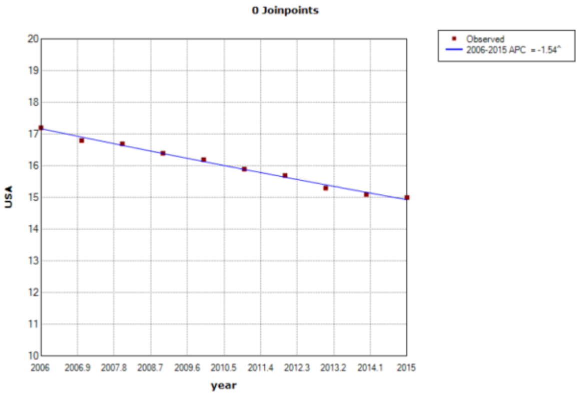

Asia

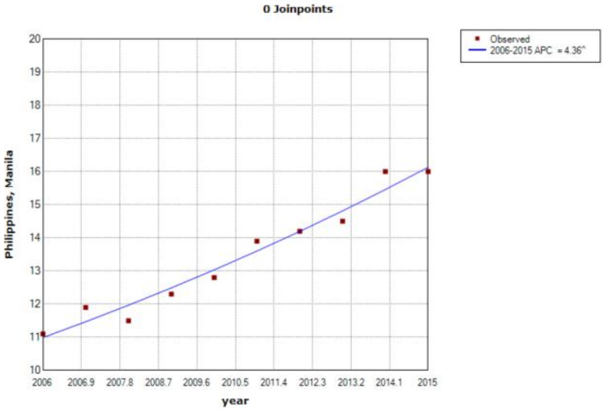

0 Joinpoints
Observed
2006-2015 APC = 4.36^
Philippines, Manila
year

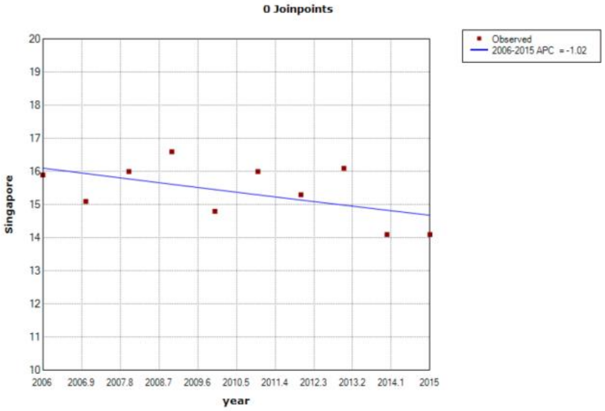

0 Joinpoints
Observed
2006-2015 APC = -1.02
Singapore
year

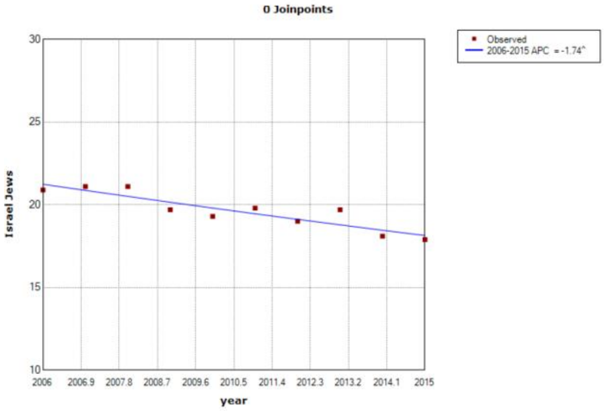

0 Joinpoints
Observed
2006-2015 APC = -1.74^
Israel Jews
year

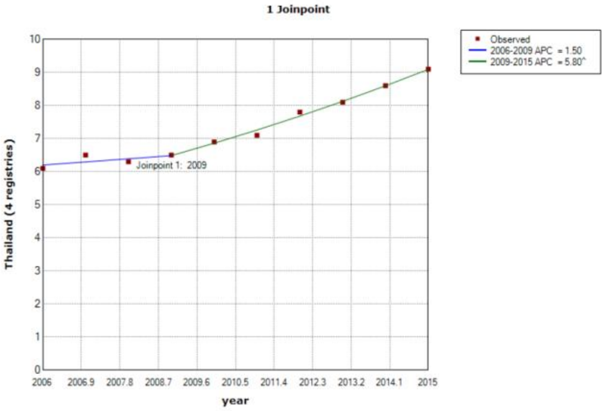

1 Joinpoint
Observed
2006-2009 APC = 1.50
2009-2015 APC = 5.80^
Joinpoint 1: 2009
Thailand (4 registries)
year

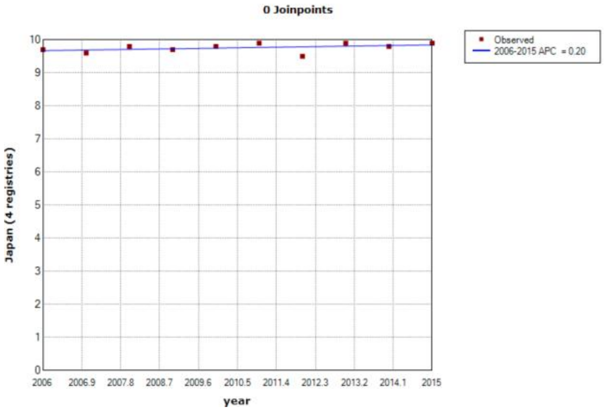

0 Joinpoints
Observed
2006-2015 APC = 0.20
Japan (4 registries)
year

Oceania

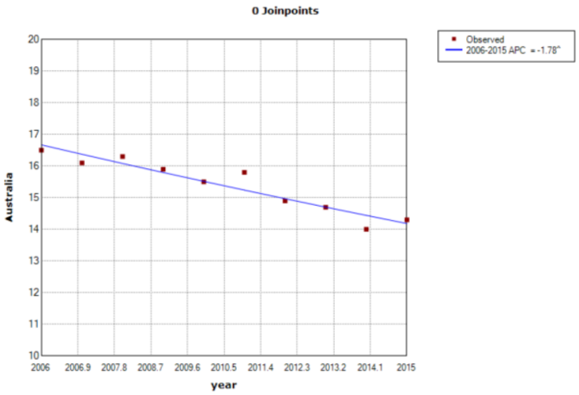

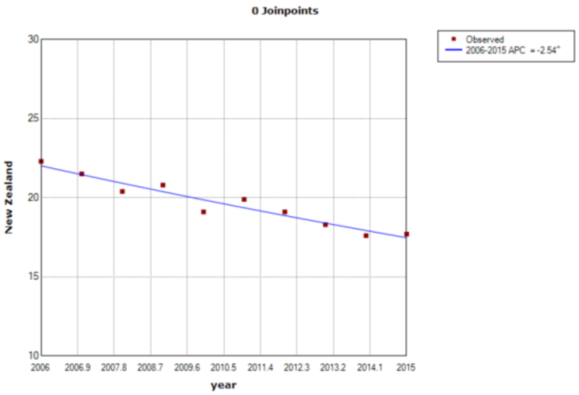

Northern Europe

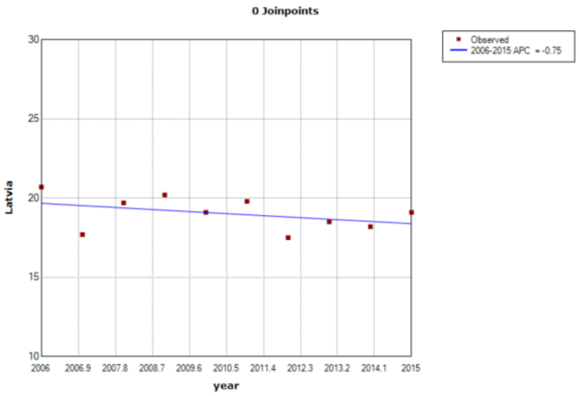

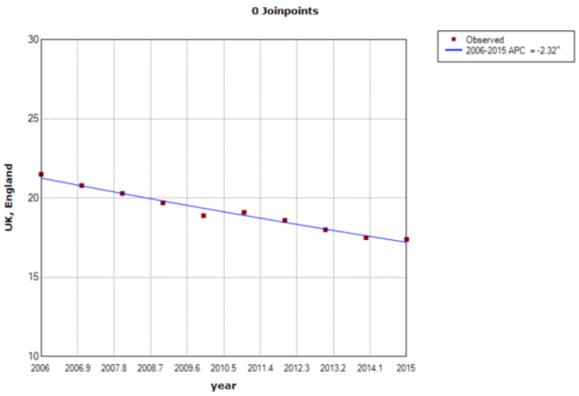

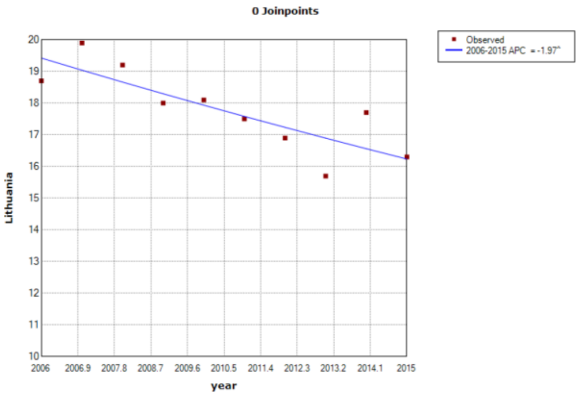

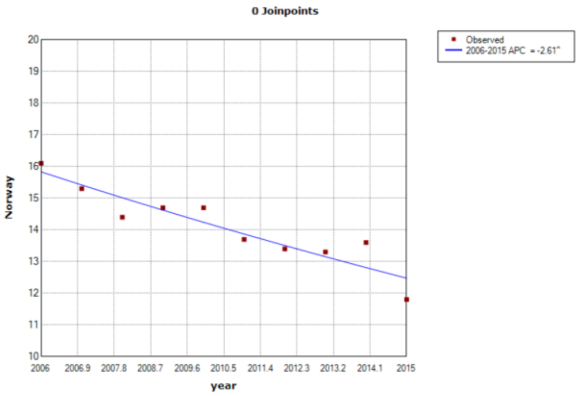

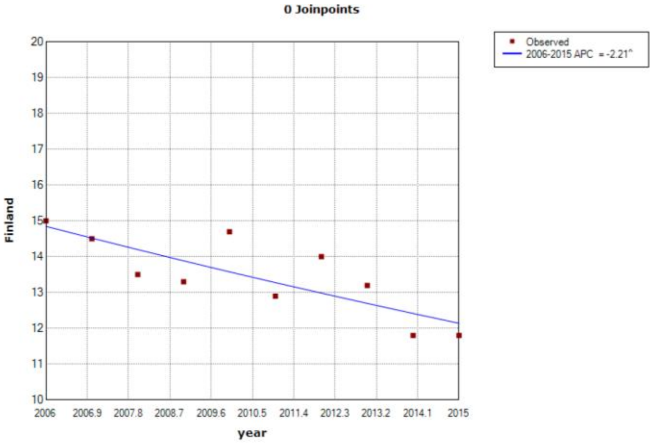
0 Joinpoints
Observed
2006-2015 APC = -2.21^
Finland
year

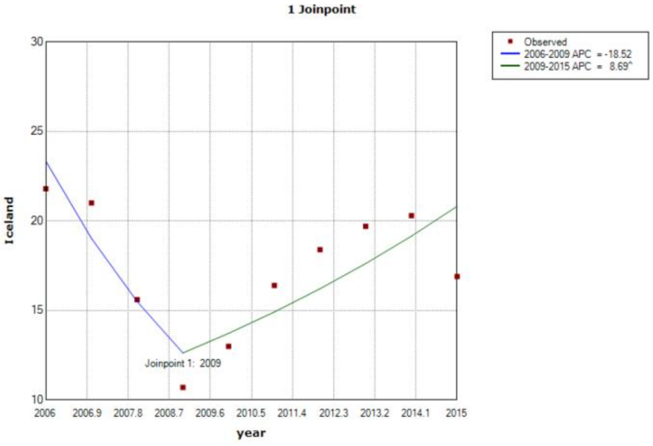
1 Joinpoint
Observed
2006-2009 APC = -18.52
2009-2015 APC = 8.69^
Joinpoint 1: 2009
Iceland
year

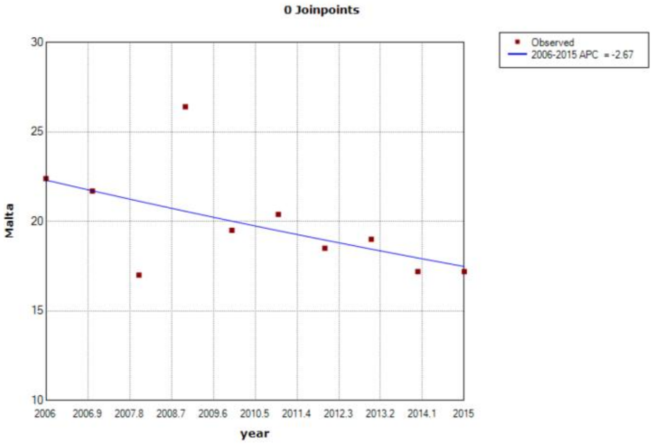
0 Joinpoints
Observed
2006-2015 APC = -2.67
Malta
year

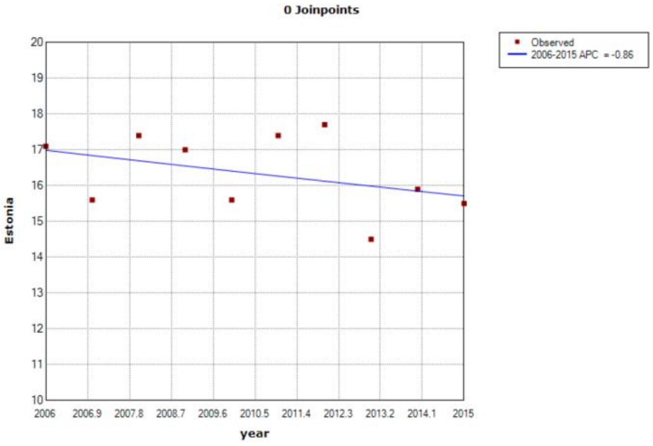
0 Joinpoints
Observed
2006-2015 APC = -0.86
Estonia
year

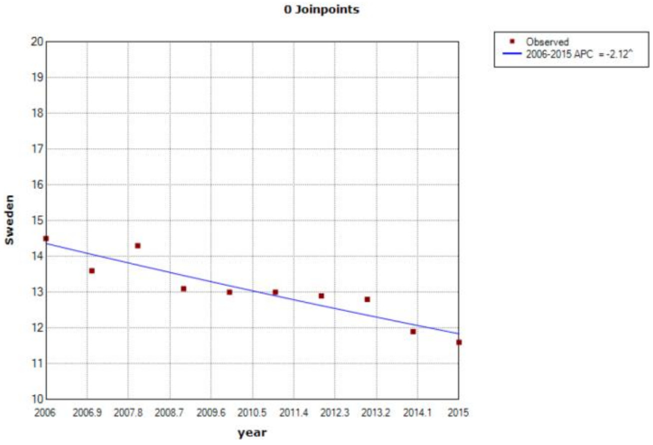
0 Joinpoints
Observed
2006-2015 APC = -2.12^
Sweden
year

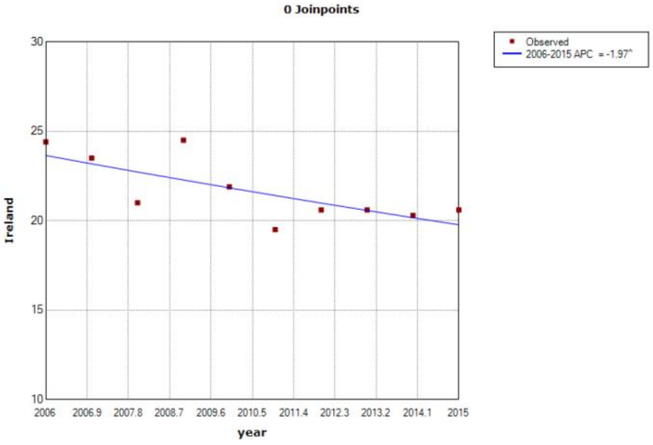
0 Joinpoints
Observed
2006-2015 APC = -1.97^
Ireland
year

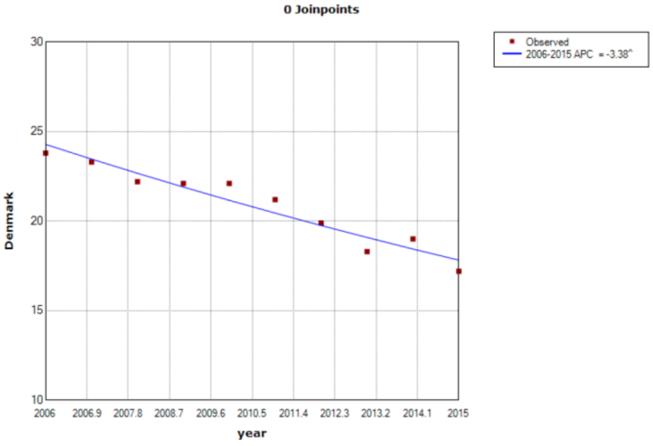

Western Europe

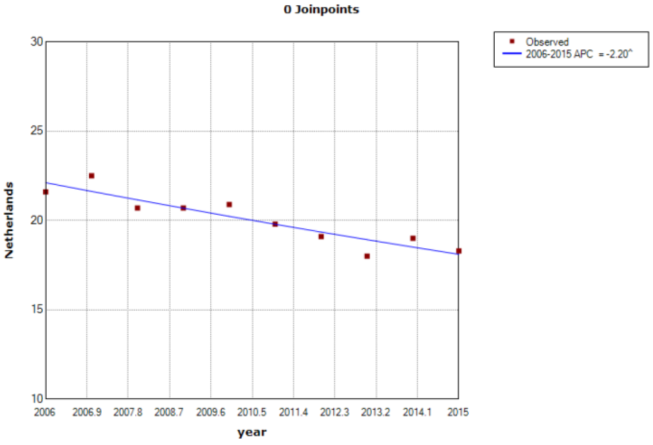

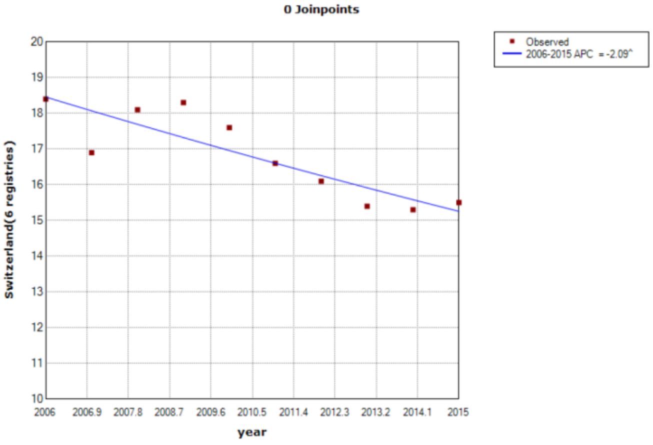

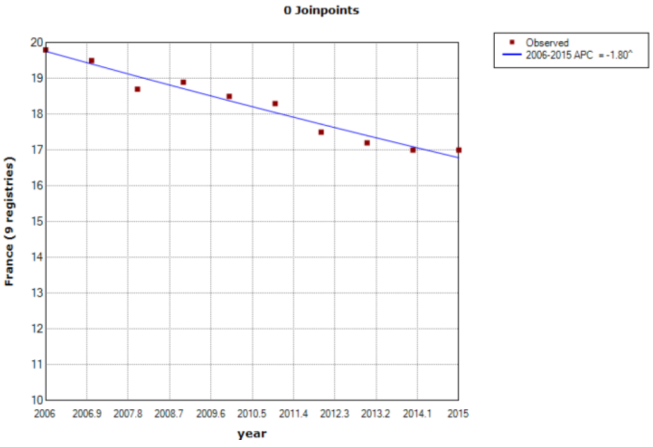

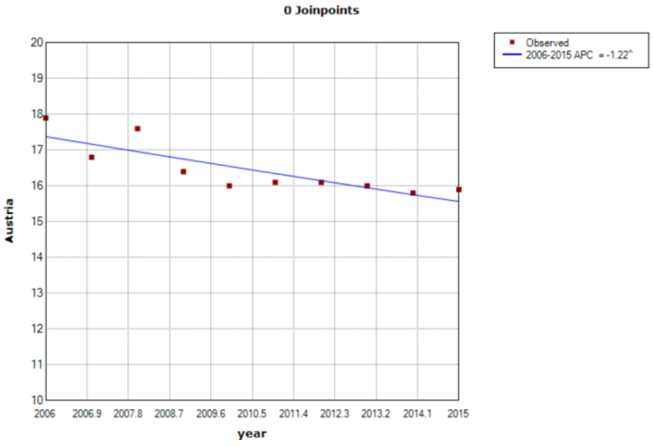

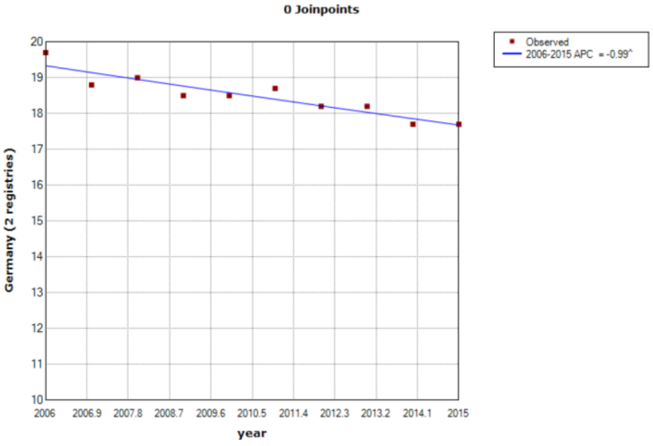

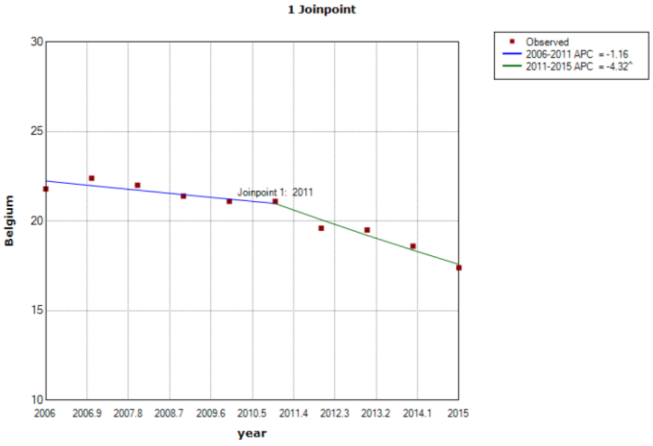

Southern Europe

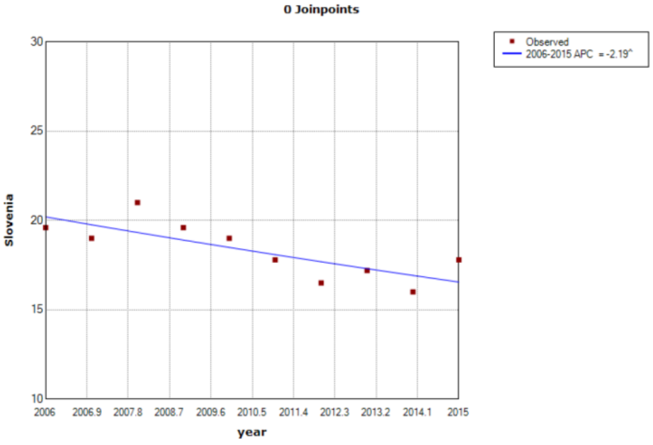

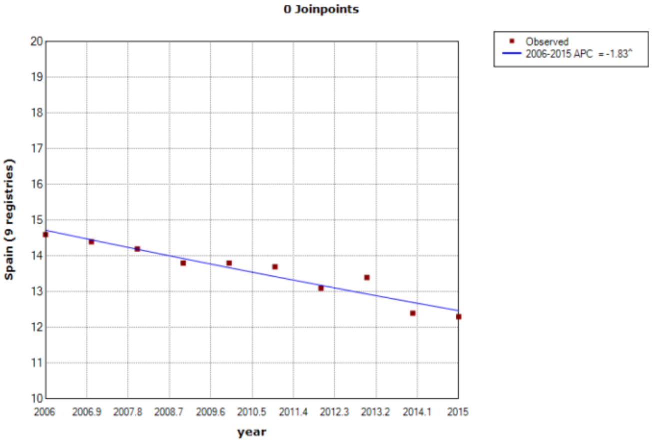

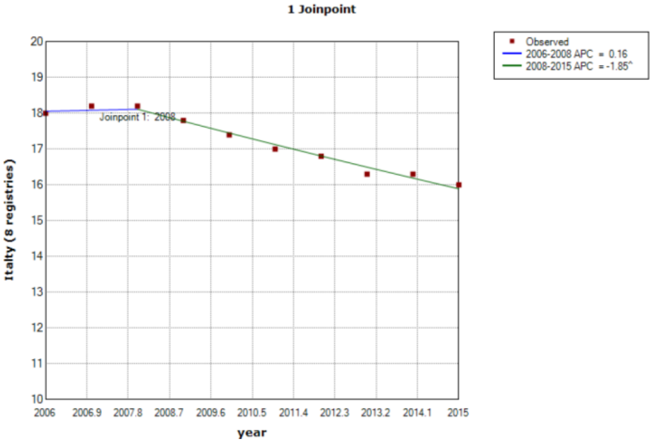

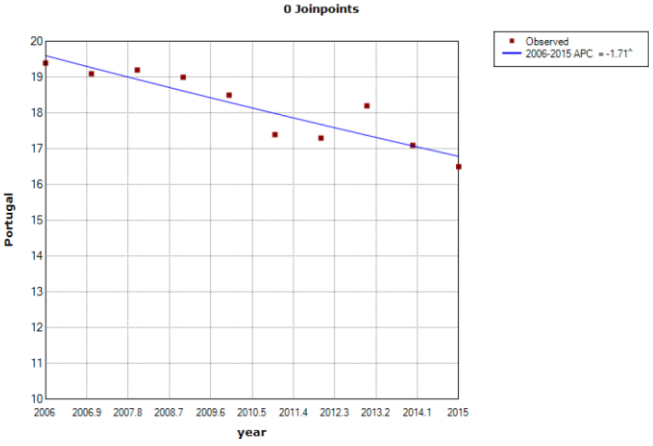

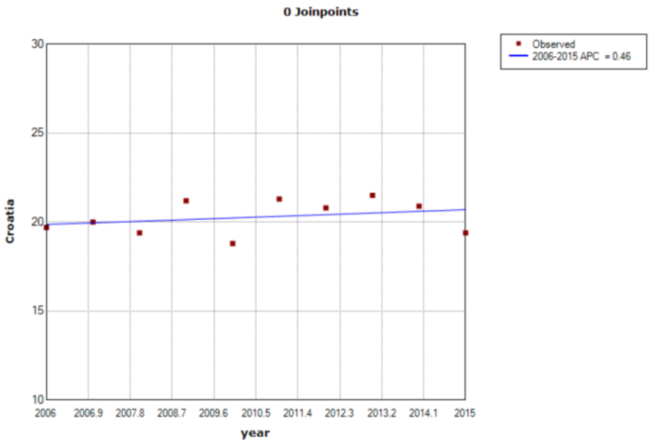

Eastern Europe

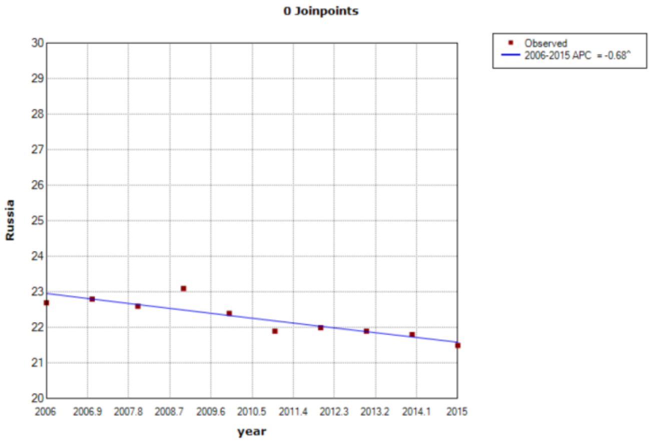

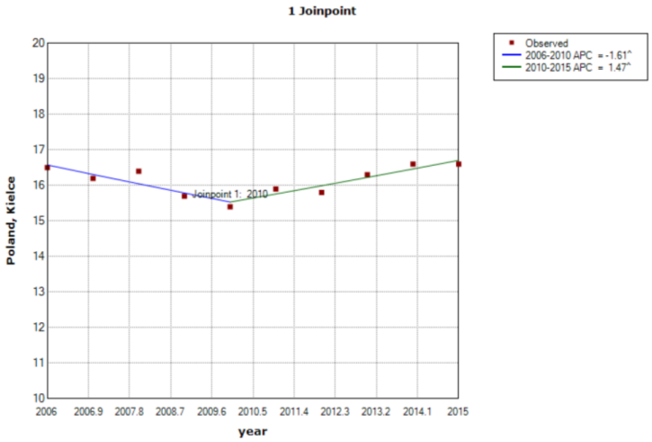

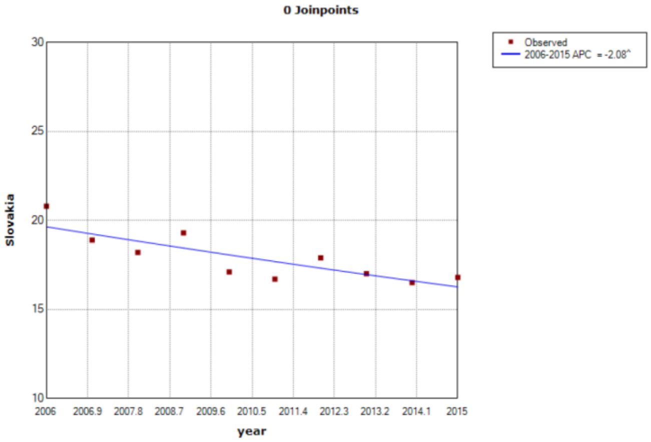

Supplementary Figure 2. The joinpoint regression analysis of breast cancer by country.
