# Supplementary material for: Global incidence and mortality of breast cancer: a trend analysis
Source: Aging (Albany NY). 2021 Feb 11;13(4):5748–803. doi: 10.18632/aging.202502 (PMC7950292; doi:10.18632/aging.202502)
Supplement: Supplementary Tables [file aging-13-202502-s002.pdf]

SUPPLEMENTARY TABLES

Supplementary Table 1. The incidence/mortality of breast cancer by region.

Region	Incidence		Deaths	
	New cases	Cum. risk	New cases	Cum. risk
Eastern Africa	40 310	3.15	20 165	1.62
Middle Africa	14 486	2.89	7 864	1.64
Northern Africa	53 917	5.06	20 058	1.96
Southern Africa	14 820	4.93	5 002	1.60
Western Africa	45 157	3.92	20 983	1.92
Caribbean	14 097	5.50	5 496	1.95
Central America	35 349	4.17	9 341	1.14
South America	150 288	6.19	37 721	1.45
North America	262 347	9.32	46 963	1.38
Eastern Asia	476 509	4.15	119 678	0.93
South-Eastern Asia	137 514	4.17	50 935	1.61
South-Central Asia	241 077	2.81	123 060	1.53
Western Asia	55 914	4.81	16 904	1.45
Central and Eastern Europe	149 024	6.10	49 951	1.80
Western Europe	169 640	9.90	41 629	1.65
Southern Europe	119 577	8.51	28 064	1.41
Northern Europe	84 272	9.63	18 063	1.46
Australia and New Zealand	22 062	10.16	3 631	1.37
Melanesia	2 116	5.30	1 046	2.73
Polynesia	252	7.46	78	2.46
Micronesia	121	4.44	47	1.71
Low HDI	105 620	3.40	52 846	1.78
Medium HDI	402 800	3.34	183 827	1.61
High HDI	666 731	4.29	184 014	1.12
Very high HDI	912 469	8.16	205 616	1.44
World	2 088 849	5.03	626 679	1.41

Supplementary Table 2. Data source of the age-standardized incidence/mortality.

	Incidence	Mortality
Austria	CI5 (1998-2012)	WHO (1980-2016)
Australia	CI5 (1993-2012)	WHO (1979-2015)
Belgium	n/a	WHO(1979-2015)
Brazil	CI5 (1993-2012)1	WHO (1979-2015)
Canada	CI5 (1983-2012)2	WHO (1979-2013)
China	CI5 (1998-2012)3	n/a
Colombia	CI5 (1983-2012)4	WHO (1984-2015)
Costa Rica	CI5 (1982-2011)	WHO (1980-2014)
Croatia	CI5 (1988-2012)	WHO (1985-2016)
Czech Republic	CI5 (1983-2012)	WHO (1986-2016)
Denmark	CI5 (1953-2012)	WHO (1994-2015)
Ecuador	CI5 (1985-2012)5	WHO (1979-2015)
Estonia	CI5 (1983-2012)	WHO (1981-2015)
Finland	NORDCAN (1953-2015)	NORDCAN (1953-2015)
France	CI5 (1998-2011)6	WHO (1979-2014)
Germany	CI5 (1998-2012)7	WHO (1990-2015)
Iceland	CI5 (1958-2012)	WHO (1981-2016)
India	CI5(1983-2012)8	n/a
Ireland	CI5 (1994-2012)	WHO (1979-2014)
Israel	CI5 (1963-2012)9	WHO (1979-2015)
Italy	CI5 (1998-2010)10	WHO (1979-2015)
Japan	CI5 (1998-2010)11	WHO (1979-2015)
Latvia	n/a	WHO (1980-2015)
Lithuania	CI5 (1988-2012)	WHO (1981-2016)
Malta	CI5 (1993-2012)	WHO(1979-2015)
Netherlands	CI5 (1989-2012)	WHO (1979-2016)
New Zealand	CI5 (1983-2012)	WHO (1979-2013)
Norway	CI5 (1953-2012)	WHO (1986-2015)
Philippines	CI5 (1983-2012)12	WHO (1992-2011)
Poland	CI5 (1998-2012)13	WHO (1980-2015)
Portugal	n/a	WHO (1980-2014)
Russian Federation	n/a	WHO (1980-2015)
Singapore	n/a	WHO (1979-2015)
Slovakia	CI5(1971-2010)	WHO (1992-2014)
Slovenia	CI5(1983-2012)	WHO (1985-2015)
Spain	CI5 (1993-2010)14	WHO (1980-2015)
Sweden	NORDCAN (1960-2015)	NORDCAN (1952-2015)
Switzerland	CI5 (1998-2012)15	WHO (1995-2015)
Thailand	CI5 (1993-2012)16	WHO (1979-2016)
United Kingdom	CI5 (1994-2012)17	WHO (1979-2015)
USA	SEER(1975-2015)	SEER(1975-2015)
USA Black	SEER(1975-2015)	SEER(1975-2015)
USA White	SEER(1975-2015)	SEER(1975-2015)

n/a" not available; CI5: Cancer Incidence in Five Continents V; NORDCAN: Nordic Cancer Registries' SEER: USA: National Institutes of Health (NIH); WHO: World Health Organization.

1. Brazil, Goiania

2. Canada (excl. Nunavut, Quebec and Yukon)

3. China (5 registries)

4. Colombia, Cali

5. Ecuador, Quito

6. France (9 registries)

7. Germany (2 registries)

8. India, Chennai

9. Israel: Jews

10. Italy (8 registries)

11. Japan (4 registries)

12. Philippines, Manila

13. Poland, Kielce

14. Spain (9 registries)

15. Switzerland (6 registries)

16. Thailand (4 registries)

17. UK, England

REFERENCE

1. SEER: http://seer.cancer.gov/data/seerstat/

2. NORDCAN: http://www-dep.iarc.fr/NORDCAN/english/frame.asp

3. WHO: http://apps.who.int/healthinfo/statistics/mortality/whodpms/

4. CI5: http://ci5.iarc.fr/CI5plus/Pages/table1_sel.aspx
